# Synthesis of five- and six-membered cyclic organic peroxides: Key transformations into peroxide ring-retaining products

**DOI:** 10.3762/bjoc.10.6

**Published:** 2014-01-08

**Authors:** Alexander O Terent'ev, Dmitry A Borisov, Vera A Vil’, Valery M Dembitsky

**Affiliations:** 1 N. D. Zelinsky Institute of Organic Chemistry, Russian Academy of Sciences, Leninsky Prospect 47, Moscow, 119991, Russia; 2 Institute for Drug Research, P.O. Box 12065, Hebrew University, Jerusalem 91120, Israel

**Keywords:** cyclic peroxides, 1,2-dioxanes, 1,2-dioxenes, 1,2-dioxolanes, ozonides, 1,2,4,5-tetraoxanes, 1,2,4-trioxanes, 1,2,4-trioxolanes

## Abstract

The present review describes the current status of synthetic five and six-membered cyclic peroxides such as 1,2-dioxolanes, 1,2,4-trioxolanes (ozonides), 1,2-dioxanes, 1,2-dioxenes, 1,2,4-trioxanes, and 1,2,4,5-tetraoxanes. The literature from 2000 onwards is surveyed to provide an update on synthesis of cyclic peroxides. The indicated period of time is, on the whole, characterized by the development of new efficient and scale-up methods for the preparation of these cyclic compounds. It was shown that cyclic peroxides remain unchanged throughout the course of a wide range of fundamental organic reactions. Due to these properties, the molecular structures can be greatly modified to give peroxide ring-retaining products. The chemistry of cyclic peroxides has attracted considerable attention, because these compounds are used in medicine for the design of antimalarial, antihelminthic, and antitumor agents.

## Introduction

**Figure 1:**
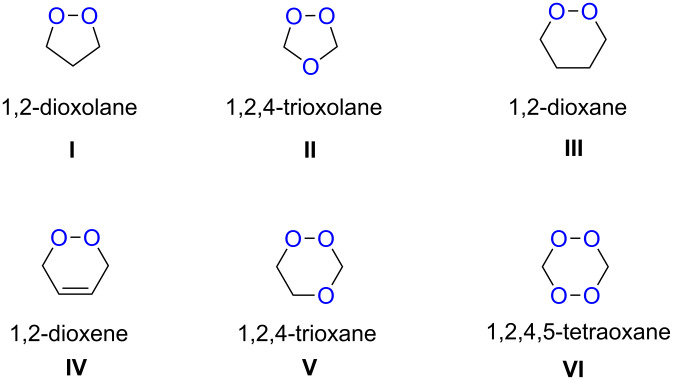
Five and six-membered cyclic peroxides.

Approaches to the synthesis of five and six-membered cyclic peroxides, such as 1,2-dioxolanes **I**, 1,2,4-trioxolanes (ozonides) **II**, 1,2-dioxanes **III**, 1,2-dioxenes **IV**, 1,2,4-trioxanes **V**, and 1,2,4,5-tetraoxanes **VI**, published from 2000 to present are reviewed. These compounds are widely used in synthetic and medicinal chemistry (Figure 1).

In the last decade, two reviews on this rapidly progressing field were published by McCullough and Nojima [1] and Korshin and Bachi [2] covering earlier studies. There are several review articles on medicinal chemistry of peroxides, where the problems of their synthesis are briefly considered. In addition to these reviews other publications dealing with this subject appeared: Tang et al. [3], O’Neill, Posner and colleagues [4–5], Masuyama et al. [6], Van Ornum et al. [7], Jefford [8–9], Dembitsky et al. [10–15], Opsenica and Šolaja [16], Muraleedharan and Avery [17], and other [18–27] including dissertations [28–32].

Reviews published earlier on the chemistry of ozone [33–36] and on the chemistry and biological activity of natural peroxides, and cyclic peroxides [37–46] are closely related to this review. Generally speaking, state-of-the-art approaches to the synthesis of cyclic peroxides are based on three key reagents: oxygen, ozone, and hydrogen peroxide. These reagents and their derivatives are used in the main methods for the introduction of the peroxide group, such as the singlet-oxygen ene reaction with alkenes, the [4 + 2]-cycloaddition of singlet oxygen to dienes, the Mukaiyama–Isayama peroxysilylation of unsaturated compounds, the Kobayashi cyclization, the nucleophilic addition of hydrogen peroxide to carbonyl compounds, the ozonolysis, and reactions with the involvement of peroxycarbenium ions.

Each part of the review deals with a particular class of the above-mentioned peroxides in accordance with an increase in the number of oxygen atoms and the ring size. In the individual sections, the data are arranged mainly according to the common key step in the synthesis of the cyclic peroxides. Examples of the synthesis of peroxide derivatives via modifications of functional groups, with the peroxide bond remaining unbroken, are given in the end of each chapter. In most cases, the syntheses of compounds having high biological activity are considered.

Currently, the rapid progress in chemistry of organic peroxides is to a large degree determined by their high biological activity. In medicinal chemistry of peroxides, particular emphasis is given to the design of compounds having activity against causative agents of malaria and helminth infections. The World Health Organization (WHO) considers malaria as one of the most dangerous social diseases. Worldwide, 300–500 million cases of malaria occur each year, and 2 million people die from it [47–48].

**Figure 2:**
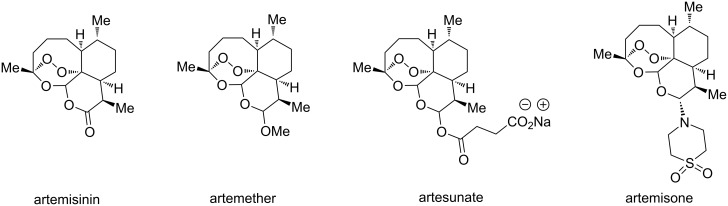
Artemisinin and semi-synthetic derivatives.

Due to a high degree of resistance in malaria to traditional drugs as quinine, chloroquine, and mefloquine, an active search for other classes of new drugs is performed. In this respect, organic peroxides play a considerable role. In medicinal chemistry of peroxides, artemisinin a natural peroxide exhibiting high antimalarial activity, is the most important drug in use for approximately 30 years. Artemisinin was isolated in 1971 from leaves of annual wormwood (*Artemesia annua*) [49–51]; the 1,2,4-trioxane ring **V** is the key pharmacophore of these drugs. A series of semi-synthetic derivatives of artemisinin were synthesized: artesunate, artemether, and artemisone (Figure 2). Currently, drugs based on these compounds are considered as the most efficacious for the treatment of malaria [52–76].

The discovery of arterolane, a synthetic 1,2,4-trioxolane, is a considerable success in the search for easily available synthetic peroxides capable of replacing artemisinin and its derivatives in medical practice. Currently, this compound is currently in phase III clinical trials [77–81].

The mechanism of antimalarial action of peroxides is unusual for pharmaceutical chemistry. According to the commonly accepted mechanism, peroxides diffuse into *Plasmodium*-infected erythrocytes, and the heme iron ion of the latter reduces the peroxide bond to form a separated oxygen-centered radical anion, which rearranges to the C-centered radical having a toxic effect on *Plasmodium* [82–87].

In the course of the large-scale search for synthetically accessible and cheap antimalarial peroxides (compared with natural and semi-synthetic structures), it was found that structures containing 1,2-dioxolane [88–90], 1,2,4-trioxolane [91–101], 1,2-dioxane [102–112], 1,2-dioxene [113–119], 1,2,4-trioxane [120–127] or 1,2,4,5-tetraoxane rings [128–146] exhibit pronounced activity, and in some cases, even superior to that of artemisinin.

Another important field of medicinal chemistry of organic peroxides includes the search for antihelminthic drugs. For example, compounds containing 1,2-dioxolane [147], 1,2,4-trioxolane [148–152], 1,2,4-trioxane [153–158] or bridged 1,2,4,5-tetraoxane [159] moieties show activity against *Schistosoma*. Schistosomiasis is one of the most widespread helminthic diseases; 800 million people are at risk of acquiring this infection [160–174].

Additionally, based on synthetic peroxides, several compounds exhibiting antitumor activity were synthesized. These compounds contain 1,2-dioxolane [10–15,175–178], 1,2-dioxane [10–15,112,178–181], 1,2-dioxene [114,182–185] or 1,2,4-trioxane [10–15,175–176] rings. More than 300 peroxides are known to have a toxic effect on cancer cells [10–15,73,186–206].

Synthetic peroxides exhibit also other activities. For example, compounds containing the 1,2,4-trioxane ring are active against *Trichomonas* [207], compounds with the 1,2-dioxane ring show antitrypanosomal and antileishmanial activities [208–212], and compounds containing the 1,2-dioxene ring possess fungicidal [210,213–224] and antimycobacterial activities [128–131,225–228]. The present review covers literature relating to 5- and 6-membered cyclic peroxide chemistry published between 2000 and 2013.

## Review

### 1. Synthesis of 1,2-dioxolanes

The modern approaches to the synthesis of 1,2-dioxolanes are based on the use of oxygen and ozone for the formation of the peroxide moiety, the Isayama–Mukaiyama peroxysilylation, and reactions involving peroxycarbenium ions. Syntheses employing hydrogen peroxide and the intramolecular Kobayashi cyclization are less frequently used.

#### 1.1. Use of oxygen for the peroxide ring formation

**Scheme 1:**
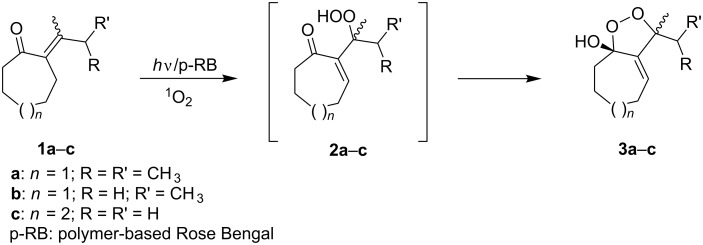
Synthesis of 3-hydroxy-1,2-dioxolanes **3a**–**c**.

The singlet-oxygen ene reaction with alkenes provides an efficient tool for introducing the hydroperoxide function. The reaction starts with the coordination of oxygen to the double bond followed by the formation of hydroperoxides presumably by a stepwise or concerted mechanism [229–230]. The oxidation of α,β-unsaturated ketones **1a–c** by singlet oxygen affords 3-hydroxy-1,2-dioxolanes **3a–c** via the formation of β-hydroperoxy ketones **2a–c** (Scheme 1) [231].

**Scheme 2:**
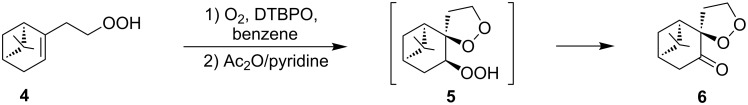
Synthesis of dioxolane **6**.

Dioxolane **6** was synthesized in 36% yield by the reaction of oxygen with hydroperoxide **4** in the presence of di-*tert*-butyl peroxalate (DTBPO) followed by the treatment of the reaction mixture with acetic anhydride and pyridine at room temperature (Scheme 2).

It should be emphasized that a mixture of dioxolanes **5** and **6** in a ratio of 7:3 is formed already in the first step [232].

**Scheme 3:**
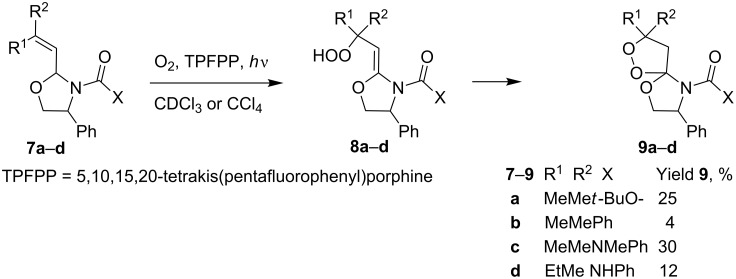
Photooxygenation of oxazolidines **7a**–**d** with formation of spiro-fused oxazolidine-containing dioxolanes **9a**–**d**.

The photooxygenation of oxazolidines **7a**–**d** through the formation of hydroperoxides **8a–d** gives spiro-fused oxazolidine-containing dioxolanes **9a–d** in low yields (12–30%) (Scheme 3) [233].

The reaction was performed in a temperature range from −10 to −5 °С. The conversion of oxazolidines **7** and the yields of dioxolanes **9** were determined by ^1^H NMR spectroscopy.

**Scheme 4:**
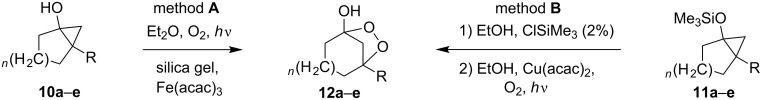
Oxidation of cyclopropanes **10a–e** and **11a–e** with preparation of 1,2-dioxolanes **12a**–**e**.

An efficient method for the synthesis of 1,2-dioxolanes is based on the oxidation of cyclopropanes by oxygen in the presence of transition-metal salts as the catalysts. The reactions of bicycloalkanols **10a**–**e** with singlet oxygen in the presence of catalytic amounts of Fe(III) acetylacetonate produce peroxides **12a**–**e**, which can also be synthesized starting from silylated bicycloalkanols **11a**–**e** with the use of Cu(II) acetylacetonate (Scheme 4, Table 1) [234].

**Table 1:** Structures and yields of dioxolanes **12a**–**e**.

Bicycloalkanol **10a–e**, silylated bicycloalkanol **11a**–**e**			1,2-Dioxolane **12a**–**e**			

			Method A^a^		Method B^b^	
	R	*n*	Reaction time, h	Yield, %	Reaction time, h	Yield, %

**a**	CH_3_	1	3	35	5	54
**b**	C_4_H_9_	1	3	55	3.5	84
**c**	C_6_H_13_	1	3	68	–	–
**d**	CH_2_Ph	1	3	50	5	78
**e**	CH_3_	2	36	54	6	80

^a^Et_2_O, O_2_, *h*ν, silica gel, Fe(acac)_3_ (4 mol %). ^b^EtOH, O_2_, *h*ν, Cu(acac)_2_ (4 mol %).

**Scheme 5:**
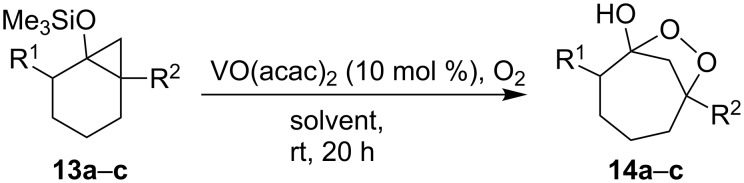
VO(acac)_2_-catalyzed oxidation of silylated bicycloalkanols **13a**–**c**.

Similarly, the reactions of silylated bicycloalkanols **13a**–**c** with oxygen in the presence of the catalyst VO(acac)_2_ yielded dioxolanes **14a**–**c**, which made it possible to perform the oxidation without irradiation (Scheme 5, Table 2) [235].

**Table 2:** Structures and yields of dioxolanes **14a**–**c**.

Silylated bicycloalkanol **13a**–**c**				

	R^1^	R^2^	Solvent	Yield **14a**–**c**, %

**a**	H	Me	EtOH	45
			CF_3_CH_2_OH	86
**b**	H	Bn	CF_3_CH_2_OH	43
**c**	Me	Me	CF_3_CH_2_OH	43

This reaction gives β-hydroxyketones as by-products that are formed as a result of the decomposition of dioxolanes **14**.

**Scheme 6:**
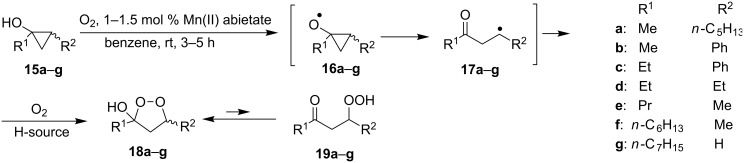
Mn(II)-catalyzed oxidation of cyclopropanols **15a**–**g**.

Cyclopropanols **15a**–**g** are readily oxidized by molecular oxygen in the presence of Mn(II) abietate or acetylacetonate (Scheme 6) [236].

Presumably, the reaction proceeds via the intermediate formation of О- and С-centered radicals **16a**–**g** and **17a**–**g**, respectively. According to this method, dioxolanes **18a**–**g** (exist in equilibrium with the open form **19a**–**g**) were synthesized in 60–80% yields.

**Scheme 7:**
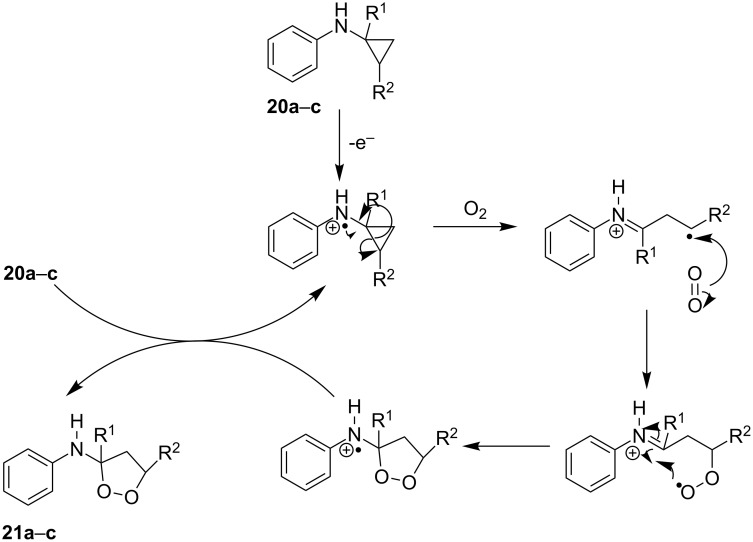
Oxidation of aminocyclopropanes **20a–c**.

Like hydroxycyclopropanes, aminocyclopropanes are transformed into 1,2-dioxolanes. For example, *N*-cyclopropyl-*N*-phenylamines **20a–c** form dioxolanes **21a–c** in the presence of atmospheric oxygen (Table 3). It was found that the reaction rate substantially increases in the presence of catalytic amounts of [(phen)_3_Fe(III)(PF_6_)_3_] or equimolar amounts of benzoyl peroxide or di-*tert*-butyl peroxide. The possible mechanism of the oxidation is shown in Scheme 7 [237].

**Table 3:** Peroxidation of *N*-cyclopropyl-*N*-phenylamines **20a–c** to form 3-(1,2-dioxalanyl)-*N*-phenylamines **21a–c**.

Dioxolane **21a–c**			

	R^1^	R^2^	Reaction conditions

**a**	H	H	1. (BzO)_2_ (1 mol/1 mol **20a**), CHCl_3_, dark, −20 °C, 3 days. 2. (*t-*BuO)_2_ (1 mol/1 mol **20a**), CHCl_3_, UV (254 nm), ambient temperature, aerobic, 2 h. 3. [(phen)_3_Fe(III)(PF_6_)_3_] (0.6 % mol), CHCl_3_, ambient temperature, aerobic, 1 h.
**b**	Me	H	1. (*t-*BuO)_2_ (1 mol/1 mol **20b**), CHCl_3_, UV (254 nm), ambient temperature, aerobic, 2 h.
**c**	H	Me	1. (*t-*BuO)_2_ (1 mol/1 mol **20c**), CHCl_3_, UV (254 nm), ambient temperature, aerobic, 2 h. 2. [(phen)_3_Fe(III)(PF_6_)_3_] (0.6 % mol), CHCl_3_, ambient temperature, aerobic, 1 h.

According to the ^1^H NMR data, dioxolanes **21a–c** are formed under the above-mentioned conditions in almost quantitative yields; the yields based on the isolated product were not higher than 80% [237].

**Scheme 8:**
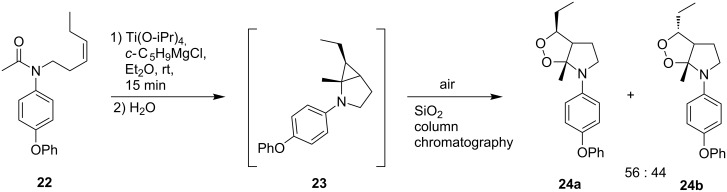
Synthesis of aminodioxolanes **24**.

Structurally similar 3-ethyl-6a-methyl-6-(4-phenoxyphenyl)hexahydro[1,2]dioxolo[3,4-*b*]pyrroles **24a** and **24b** were synthesized from (*Z*)-*N*-(hex-3-enyl)-*N*-(4-phenoxyphenyl)acetamide (**22**). It was suggested that aminocyclopropane **23** is formed in situ, which is subsequently oxidized in air on silica gel (Scheme 8) [238]. The total yield of both isomers **24** was 31%.

**Figure 3:**
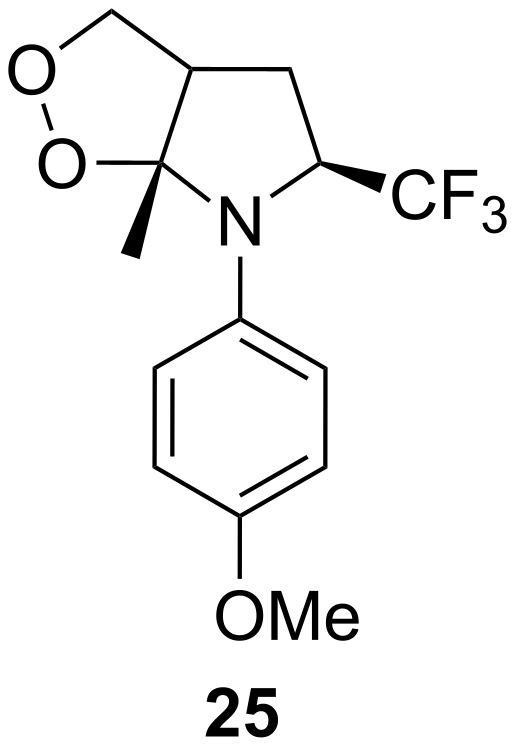
Trifluoromethyl-containing dioxolane **25**.

Trifluoromethyl-containing dioxolane **25** (Figure 3) was synthesized according to this method in 40% yield [239].

**Scheme 9:**
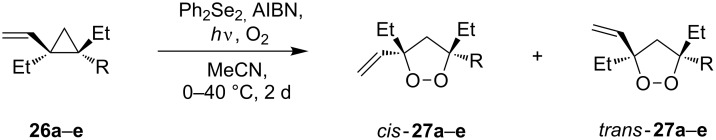
Synthesis of 1,2-dioxolanes **27a**–**e** by the oxidation of cyclopropanes **26a**–**e**.

A series of 1,2-dioxolanes **27a–e** containing various functional groups R were prepared by the oxidation of cyclopropanes **26a–e** (Scheme 9, Table 4).

**Table 4:** Structures and yields of dioxolanes **27a**–**e**.

Dioxolane **27a–e**	R	Yield (*cis* + *trans*), %	Ratio (*cis*/*trans*)

**a**	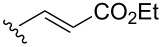	88	1/7
**b**	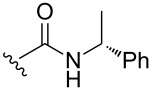	100	*trans* isomer
**c**	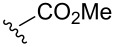	75	1/22
**d**	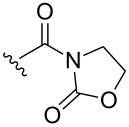	100	1/13
**e**	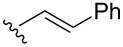	82	1/2.8

The reaction was performed in the presence of Ph_2_Se_2_ (10 mol %) and azobisisobutyronitrile (AIBN, 8 mol %) in air under irradiation for two days. The product was purified by flash chromatography to obtain a mixture of *cis* and *trans* isomers, whose ratio depends primarily on the nature of the substituent in cyclopropanes **26a**–**e** [240].

**Scheme 10:**
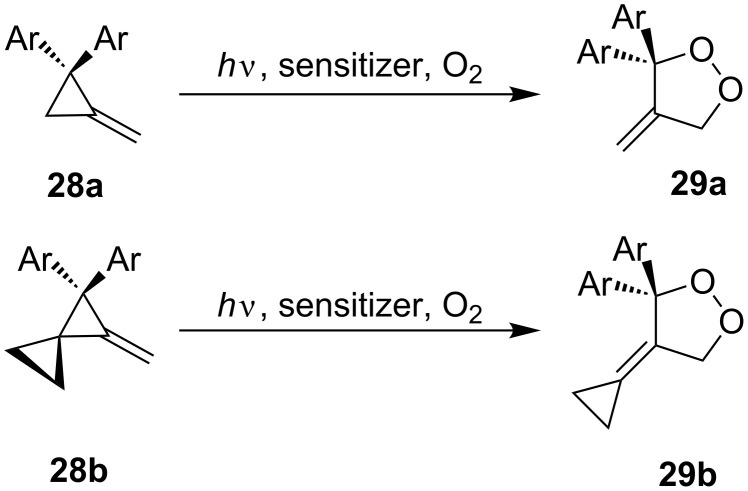
Photoinduced oxidation of methylenecyclopropanes **28**.

The oxidation of methylenecyclopropanes **28a** and **28b** under photoinduced electron-transfer conditions is described by a similar scheme (Scheme 10).

The reaction was performed in acetonitrile or in a mixture of toluene and acetonitrile with the use of 9,10-dicyanoanthracene (DCA), 1,2,4,5-tetracyanobenzene (TCNB), or *N*-methyl-quinolinium tetrafluoroborate (NMQ^+^BF_4_^−^) as sensitizers. Under these conditions, dioxolane **29a** was obtained in quantitative yield (^1^H NMR data), the yield of **29b** was not reported [241].

**Scheme 11:**
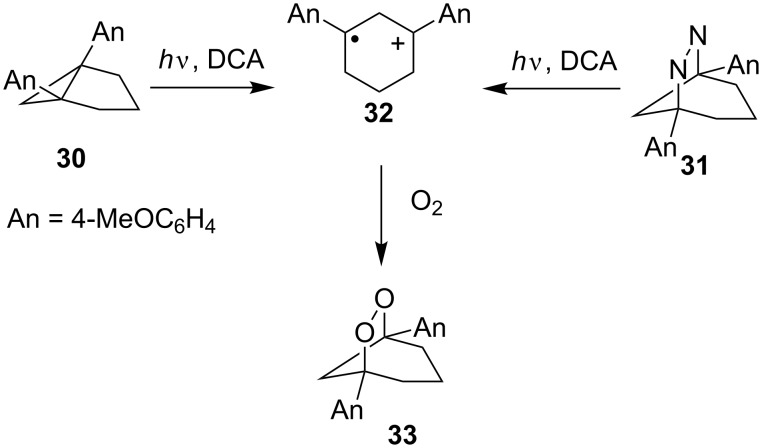
Irradiation-mediated oxidation.

Under irradiation in the presence of oxygen, 1,5-bis(4-methoxyphenyl)bicyclo[3.1.0]hexane (**30**) and 1,5-bis(4-methoxyphenyl)-6,7-diazabicyclo[3.2.1]oct-6-ene (**31**) were transformed into bicyclic dioxolane **33**. It was suggested that both reactions proceed via the formation of 1,3-radical cation **32** (Scheme 11).

Dioxolane **33** was synthesized in the highest yields (91% from **30** and 100% from **31**) in acetonitrile with the use of 9,10-dicyanoanthracene (DCA) as the sensitizer [242].

**Scheme 12:**
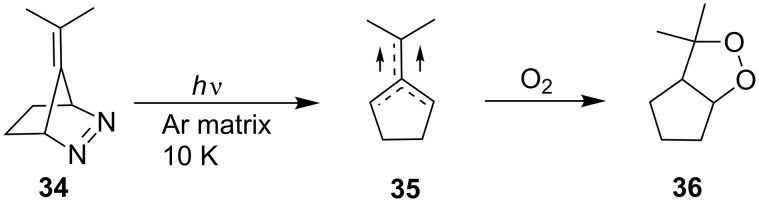
Application of diazene **34** for dioxolane synthesis.

After irradiation of diazene **34** in an argon matrix at 10 K, biradical **35** was detected by IR spectroscopy and the reaction of the latter with oxygen at 10 K proceeded regioselectively to give dioxolane **36** (Scheme 12) [243].

Bicyclic peroxide 2-heptyl-3,4-dioxabicyclo[3.3.0]oct-1(8)-ene was prepared by a similar process [244].

**Scheme 13:**
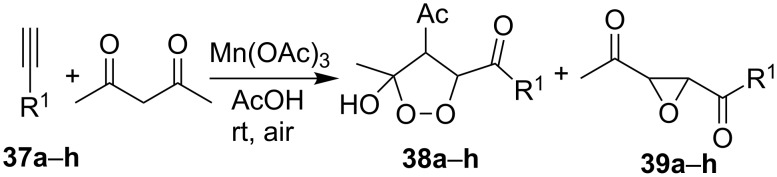
Mn(OAc)_3_-catalyzed cooxidation of arylacetylenes **37a**–**h** and acetylacetone with atmospheric oxygen.

The oxidation of arylacetylenes **37a**–**h** with atmospheric oxygen in the presence of catalytic amounts of Mn(OAc)_3_ in an excess of acetylacetone afforded dioxolanes **38a**–**h** in moderate yields (34–64%) (Scheme 13, Table 5) [245].

**Table 5:** Structures and yields of dioxolanes **38a–h** and epoxides **39a–h**.

**37a–h**	R^1^	Yield **38a–h**, %	Yield **39a–h**, %

**a**	Ph	45	5
**b**	4-MeC_6_H_4_	52	7
**c**	4-MeOC_6_H_4_	64	2
**d**	4-ClC_6_H_4_	38	2
**e**	4-FC_6_H_4_	41	6
**F**	1-naphthyl	54	6
**g**	2-naphthyl	52	8
**h**	3,4-(MeO)_2_C_6_H_3_	34	11

The reaction was performed at 23 °С in glacial acetic acid in air; the **37/**acetylacetone/Mn(OAc)_3_ molar ratio was 1/10/10. The reaction gave oxiranes **39** as by-products, which can also be synthesized in quantitative yields by the treatment of dioxolanes **38** with silica gel in methanol [245].

#### 1.2. Peroxidation of alkenes with the Co(II)/Et_3_SiH/O_2_ system (Isayama–Mukaiyama reaction)

Peroxysilylation of alkenes with molecular oxygen in the presence of triethylsilane catalyzed by cobalt(II) diketonates was described for the first time by S. Isayama and T. Mukaiyama in 1989 [246–247]. Currently, this approach is one of the main methods for the preparation of peroxides from alkenes.

**Scheme 14:**
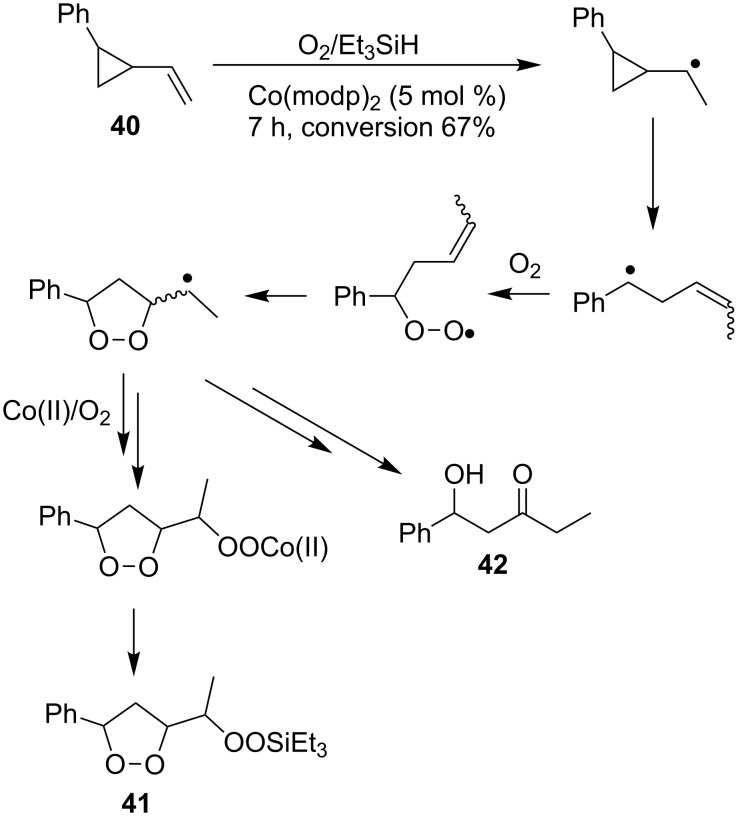
Peroxidation of (2-vinylcyclopropyl)benzene (**40**).

Compounds (oxidized by the Isayama–Mukaiyama reaction) containing a reaction center that can be subjected to the attack by a peroxide radical, are able to undergo intramolecular cyclization to form the 1,2-dioxolane ring. For example, the Co(modp)_2_-catalyzed peroxysilylation (modp = 1-morpholino-5,5-dimethyl-1,2,4-hexanetrionate) of (2-vinylcyclopropyl)benzene (**40**) affords triethyl(1-(5-phenyl-1,2-dioxolan-3-yl)ethylperoxy)silane (**41**) in 37% yield (Scheme 14).

The reaction was carried out in 1,2-dichloroethane at room temperature, and the reaction products were separated by column chromatography. 1-Hydroxy-1-phenylpentan-3-one (**42**) was isolated as a by-product in 16% yield [248].

**Scheme 15:**
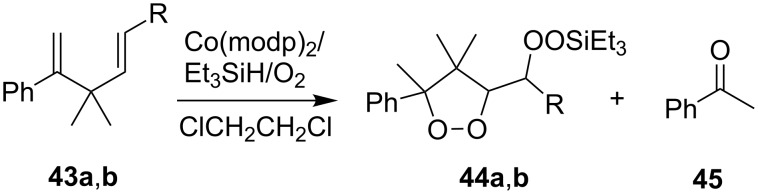
Peroxidation of 1,4-dienes **43a**,**b**.

The peroxidation of 1,4-dienes **43a**,**b** with the Co(modp)_2_/Et_3_SiH/O_2_ system according to a similar reaction scheme gave dioxolanes **44a**,**b**. Acetophenone (**45**) was obtained as the by-product (Scheme 15, Table 6) [249].

**Table 6:** Synthesis of dioxolanes **44a**,**b**.

1,4-Diene **43**	R	Reaction time, h	Conversion, %	Yield, %	

				**44**	**45**

**a**	H	4.5	47	27	49
**b**	COOEt	2	44	56	22

**Scheme 16:**
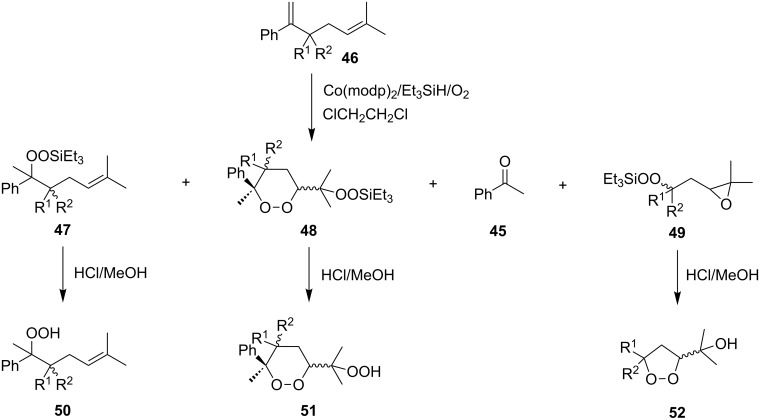
Peroxidation of 1,5-dienes **46**.

The desilylation of the initially formed silicon peroxide followed by cyclization of the hydroperoxide accompanied by the attack on the electrophilic center is another example of the use of the Isayama–Mukaiyama reaction for the synthesis of cyclic peroxides. In some cases, the reaction with 1,5-dienes **46a–d** produces, along with 1,2-dioxanes **51** (desilylation products of the corresponding 1,2-dioxanes **48**), 1,2-dioxolanes (**52b**,**d**) as a result of cyclization of the corresponding peroxysilyl epoxides **49**. In these reactions, unsaturated triethylsilyl peroxides **47** are formed as by-products, which are desilylated during hydrolysis to give the unsaturated hydroperoxides **50** (Scheme 16, Table 7) [249].

**Table 7:** Peroxidation of 1,5-dienes **46**.

Diene **46**	R^1^	R^2^	Reaction time, h	Conversion, %	Yield^a^, %				
					**45**	**49**	**50**	**51**	**52**

**a**	H	H	6	82	4	–	13	31	–
**b**	H	Me	2.5	83	36	–	12	13	33
**c**	H	Ph	3.5	75	57	38	7	27	–
**d**	Me	Me	3	84	51	–	–	31	26

^a^The yields are given based on the converted dienes **46a–d**.

**Scheme 17:**
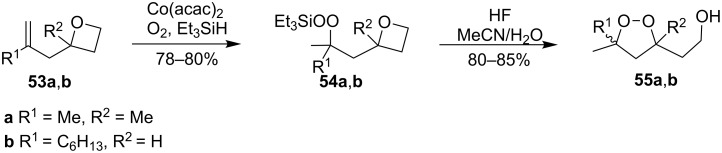
Peroxidation of oxetanes **53a**,**b**.

1,2-Dioxolanes can be produced from oxetanes **53a,b** containing a double bond in the side chain according to a similar scheme. The first step afforded peroxysilanes **54a**,**b**, which upon treatment with aqueous HF gave the target dioxolanes **55a**,**b** (Scheme 17) [250].

A similar way to 1,2-dioxolanes used an oxirane cycle for the stages of ring opening followed by 1,2-dioxolane ring closing [251].

**Scheme 18:**
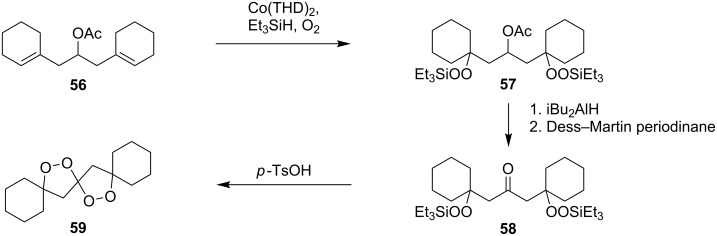
Peroxidation of 1,6-diene **56**.

The synthesis of spirodioxolane **59** involved the peroxysilylation of 1,3-dicyclohexenylpropan-2-yl acetate (**56**) catalyzed by cobalt complexed with 2,2,6,6-tetramethylheptane-3,5-dione (Co(THD)_2_) as the first step giving 1,3-bis(1-(triethylsilylperoxy)cyclohexyl)propan-2-yl acetate (**57**) that was subsequently transformed into the carbonyl-containing diperoxide (1,3-bis(1-(triethylsilylperoxy)cyclohexyl)propan-2-one) (**58**) in two steps. The latter was treated with *p*-TsOH to give the target peroxide **59** (Scheme 18) [252].

#### 1.3. The use of ozone. Peroxycarbenium ions in the 1,2-dioxolanes synthesis

The ozonolysis of unsaturated compounds is a reliable and facile method for the introduction of the peroxide functional group. As in the above-considered studies, the intramolecular cyclization of ozonolysis products can be performed with the use of the hydroperoxide group provided that there is an appropriate electrophilic center.

**Scheme 19:**
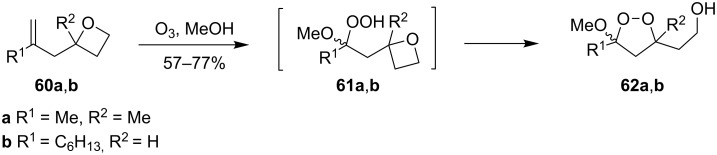
Synthesis of 3-alkoxy-1,2-dioxolanes **62a**,**b**.

The reaction of oxetanes **60a**,**b** with ozone in methanol produced 3-alkoxy-1,2-dioxolanes **62a,b**. The analysis of the reaction mixture (TLC, NMR) confirmed that cyclic peroxides are formed immediately in the reaction mixture rather than in the course of the treatment or purification of the reaction products. It was suggested that the reaction proceeds via the formation of hydroperoxy acetals **61a**,**b** (Scheme 19) [250].

**Scheme 20:**
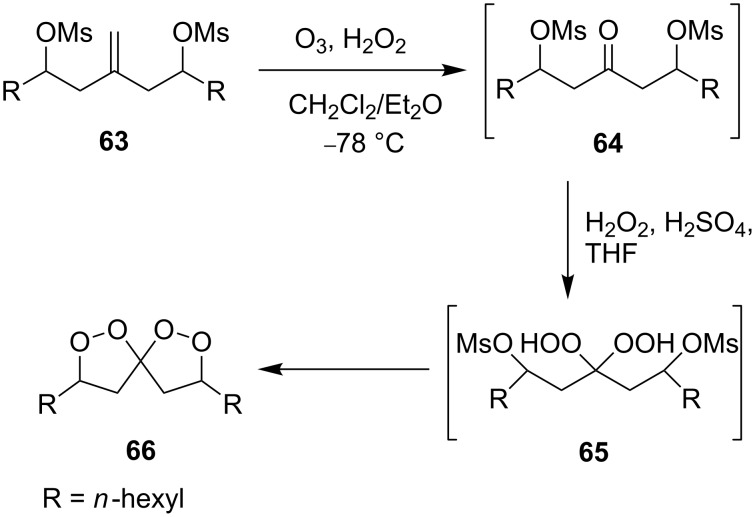
Synthesis of spiro-bis(1,2-dioxolane) **66**.

The ozonolysis of 9-methyleneheptadecane-7,11-diyl-bis(methanesulfonate) (**63**) gave 9-oxoheptadecane-7,11-diyl-bis(methanesulfonate) (**64**). The latter reacted with H_2_O_2_ in the presence of sulfuric acid (or iodine) as the catalyst to form 9,9-dihydroperoxyheptadecane-7,11-diyl-bis(methanesulfonate) **65**, and the replacement of the mesyl groups in the latter compound afforded 3,8-dihexyl-1,2,6,7-tetraoxaspiro[4.4]nonane (**66**, Scheme 20). The yield of dioxolane **66** was 36% based on **63** [252].

**Scheme 21:**
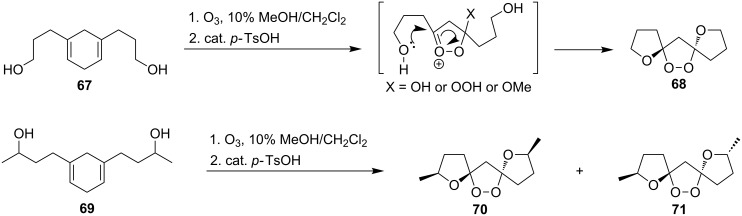
Synthesis of dispiro-1,2-dioxolanes **68**, **70**, **71**.

The treatment of 3,3'-(cyclohexa-3,6-diene-1,3-diyl)dipropan-1-ol (**67**) and 4,4'-(cyclohexa-3,6-diene-1,3-diyl)dibutan-2-ol (**69**) with ozone in MeOH/CH_2_Cl_2_ followed by the addition of a catalytic amount of *p-*TsOH lead to the intramolecular peroxyketalization that proceeds through the formation of the peroxycarbenium ion (shown in Scheme 21 for the ozonolysis of **67** as an example) to give finally dispiro-1,2-dioxolanes: 1,8,12,13-tetra-oxadispiro-[4.1.4.2]tridecane **68** (yield 67%) and two isomers of 2,9-dimethyl-1,8,12,13-tetraoxadispiro[4.1.4.2]tridecane **70** and **71** (combined yield 72%) (Scheme 21) [253].

**Scheme 22:**
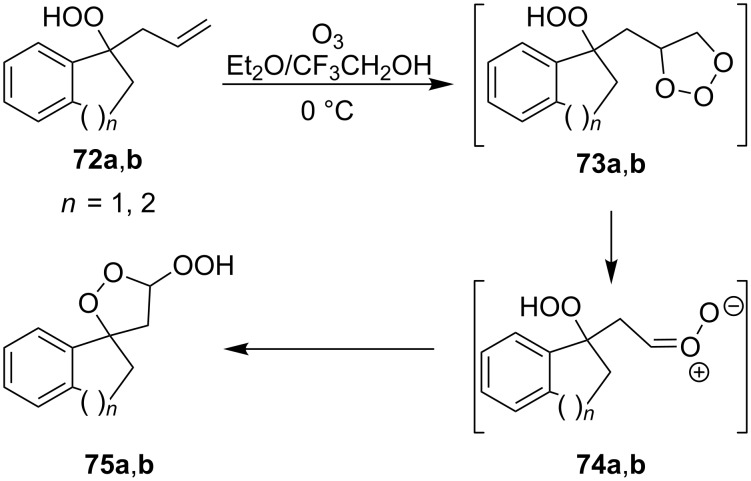
Synthesis of spirohydroperoxydioxolanes **75a,b**.

The spirohydroperoxydioxolanes, 5-hydroperoxy-2',3'-dihydrospiro[[1,2]dioxolane-3,1'-indene] (**75a**) and 5-hydroperoxy-3',4'-dihydro-2'*H*-spiro[[1,2]dioxolane-3,1'-naphthalene] (**75b**), were synthesized by the ozonolysis of 1-allyl-1-hydroperoxy-2,3-dihydro-1*H*-indene (**72a**) and 1-allyl-1-hydroperoxy-1,2,3,4-tetrahydronaphthalene (**72b**), respectively, in an Et_2_O/CF_3_CH_2_OH system (2:1). The reaction proceeds via the formation of ozonide **73** followed by elimination of formaldehyde to give peroxycarbenium ion **74** that undergoes cyclization via the attack of the hydroperoxide group on the carbon center of peroxycarbenium ion **74** (Scheme 22) [254].

Spirohydroperoxydioxolane **75a** (*n* = 1) was obtained in 71% yield (the diastereoisomeric ratio was 1:1); the yield of **75b** (*n* = 2) was 21% (the diastereoisomeric ratio was 1:1).

**Scheme 23:**
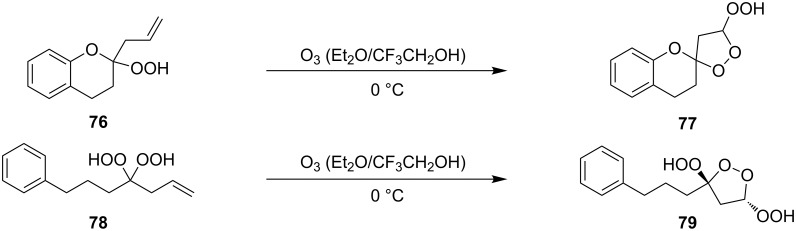
Synthesis of spirohydroperoxydioxolane **77** and dihydroperoxydioxolane **79**.

5'-Hydroperoxyspiro[chromane-2,3'-[1,2]dioxolane] (**77**, yield 18%) and (3*S*,5*S*)-3,5-dihydroperoxy-3-(3-phenylpropyl)-1,2-dioxolane (**79**, yield 22%) (Scheme 23) were synthesized in a similar way starting from 2-allyl-2-hydroperoxychromane (**76**) and (4,4-dihydroperoxyhept-6-enyl)benzene (**78**), respectively [254].

**Scheme 24:**
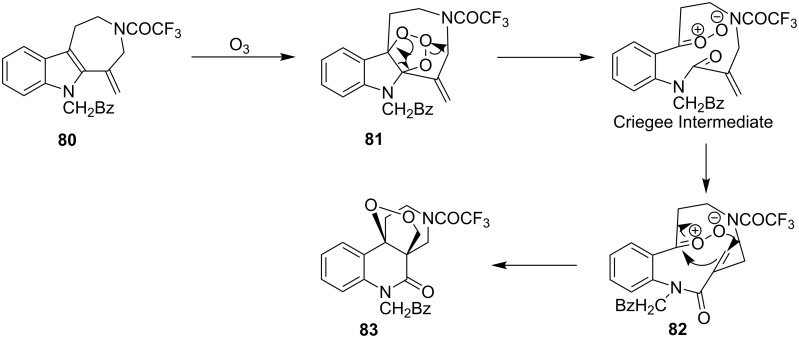
Ozonolysis of azepino[4,5-*b*]indole **80**.

An oxidative rearrangement takes place in the reaction of azepino[4,5-*b*]indole **80** with ozone. The addition of ozone to the endocyclic double bond (molozonide **81**) and the formation of the Criegee intermediate are followed by a 1,3-dipolar interaction of the peroxycarbenium ion with the double bond (**82**) to form dioxolane **83**. The yield was not lower than 48% but no exact yield was reported (Scheme 24) [255].

**Scheme 25:**
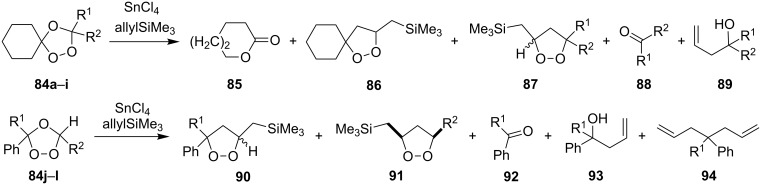
SnCl_4_-mediated fragmentation of ozonides **84a**–**l** in the presence of allyltrimethylsilane.

The peroxycarbenium ions produced by the decomposition of 1,2,4-trioxolanes can be trapped with allyltrimethylsilane. For example, the SnCl_4_-mediated fragmentation of ozonides **84a**–**l** in the presence of allyltrimethylsilane in dichloromethane gives a complex mixture of products **85**–**94**, including dioxolanes **86a–i**, **87i**, **90j–l**, and **91j** (Scheme 25, Table 8) [256].

**Table 8:** The SnCl_4_-mediated fragmentation of ozonides **84a**–**l** in the presence of allyltrimethylsilane.

				Yield, %				
	
Ozonide **84**	R^1^	R^2^	*T*, °С	Lactone **85**	Dioxolane **86**	Dioxolane **87**	Ketone **88**	Alcohol **89**

**a**	-(CH_2_)_4_-		−78 to 0	11	50	–	**88a** (traces)	–
**b**	-(CH_2_)_5_-		−78 to 0	17	57	–	**88b** (traces)	–
**c**	-(CH_2_)_6_-		−78 to 0	39	24	–	**88c** (traces)	–
**d**	CH_3_	Ph	−78 to 0	25	61	–	**88d** (93%)	–
**e**	C_4_H_9_	C_4_H_9_	−78 to 0	40	14	–	**88e** (70%)	75
**f**	H	C_8_H_17_	−78	–	56	–	–	50
**g**	H	Ph	−78	–	79	–	–	13
**h**	H	H	−78	–	10	–	–	–
**i**	CH_3_	C(CH_3_)_3_	−78 to 0	31	21	9 (*cis*)	–	–

Ozonide **84**	R^1^	R^2^	*T*, °С	Dioxolane **90** (*cis*:*trans*)	Dioxolane **91**	Carbonyl compound **92**	Alcohol **93**	Alkene **94**

**j**	H	C_3_H_7_	−78	15 (1:1)	7	39	**93j** (20%)	–
**k**	H	H	−78	15 (1:1)	–	22	**93j** (24%)	–
**l**	CH_3_	H	−78	9 (1:1)	–	43	–	2.5

**Scheme 26:**
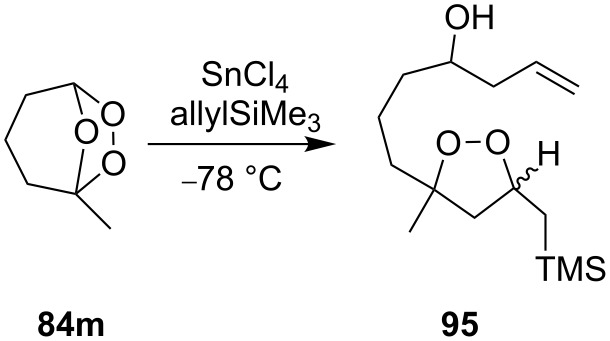
SnCl_4_-mediated fragmentation of bicyclic ozonide **84m** in the presence of allyltrimethylsilane.

Treatment of the bicyclic ozonide 1-methyl-6,7,8-trioxabicyclo[3.2.1]octane **84m**, with SnCl_4_ in the presence of allyltrimethylsilane produces a mixture of two *cis* diastereomers and two *trans* diastereomers (in a ratio of 35:35:15:15) of 7-(3-methyl-5-((trimethylsilyl)methyl)-1,2-dioxolan-3-yl)hept-1-en-4-ol **95** in a total yield of 48% (Scheme 26) [256].

These syntheses of dioxolanes involve the formation of the peroxycarbenium ion as the key step. The reaction of the latter with allyltrimethylsilane followed by the intramolecular cyclization finally leads to the dioxolane ring.

**Scheme 27:**
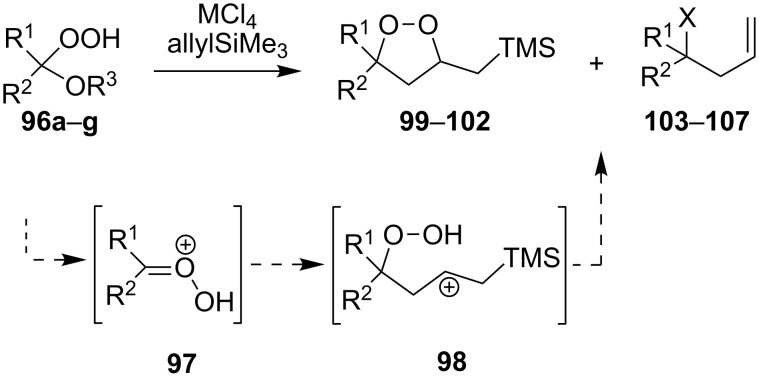
MCl_4_-mediated fragmentation of alkoxyhydroperoxides **96** in the presence of allyltrimethylsilane.

Dioxolanes **99–102** are produced from alkoxyhydroperoxides **96a–g** (ozonolysis products of alkenes) in a similar way. The first step results in the formation of peroxycarbenium ions **97**, which are trapped with allyltrimethylsilane under the formation of intermediate hydroperoxides **98**. Then either cyclic dioxolanes **99–102** or unsaturated compounds **103–107** are formed as the major reaction products depending on the nature of the substituents and the Lewis acid (Scheme 27, Table 9) [257].

**Table 9:** Synthesis of 1,2-dioxolanes **99–102**.

Hydroperoxide **96**	R^1^	R^2^	R^3^	M	Dioxolane **99–102** (yield, %)	Alkene **103–107** (X, yield, %)

**a**	Me	Me	Me	Ti	**99** (31)	–
**b**	Me	Me	(CH_2_)_2_OMe	Sn	**99** (56)	–
**b**	Me	Me	(CH_2_)_2_OMe	Ti	**99** (12)	**103** (–OOH, 23)
**с**	4-*tert-*butyl- cyclohexylidene		Me	Ti	–	**104** (–O_2_–, 31)
**c**	4-*tert-*butyl- cyclohexylidene		Me	Sn	**100** (42)	–
**d**	4-*tert-*butyl- cyclohexylidene		(CH_2_)_2_OMe	Sn	**100** (59)	–
**e**	Me	BnOCH_2_	Me	Ti	**101** (12)	**105** (=O, 62)
**f**	Bu	H	Me	Ti	**102** (7)	**106** (OMe, 63)
**g**	Bu	H	(CH_2_)_2_OMe	Ti	**102** (15)	**107** (O(CH_2_)_2_OMe, the yield was not determined)

**Scheme 28:**
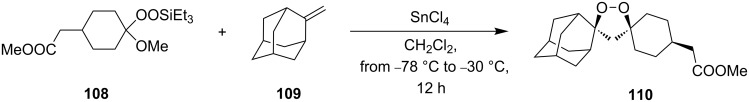
SnCl_4_-catalyzed reaction of monotriethylsilylperoxyacetal **108** with alkene **109**.

The reaction of trialkylsilylperoxyacetals with alkenes in the presence of Lewis acids also proceeds through the formation of peroxycarbenium ions. For example, the reaction of methyl 2-(4-methoxy-4-(triethylsilylperoxy)cyclohexyl)acetate (**108**) with 2-methyleneadamantane (**109**) produced adamantane-2-spiro-3’,8’-methoxycarbonylmethyl-1’,2’-dioxaspiro[4.5]decane (**110**) in 40% yield (Scheme 28) [258].

**Scheme 29:**
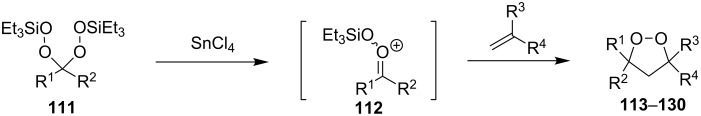
SnCl_4_-catalyzed reaction of triethylsilylperoxyacetals **111** with alkenes.

The use of easily accessible triethylsilylperoxyacetals **111** as the starting materials for the generation of silylperoxycarbenium ions **112** enabled the synthesis of 1,2-dioxolanes containing various functional groups **113–130** in good yields by the reactions with alkenes (Scheme 29, Table 10) [88,90,259].

**Table 10:** Structures and yields of 1,2-dioxolanes **113–130**.

Product	Structure	Yield**^a^**, %	Product	Structure	Yield**^a^**, %

**113**	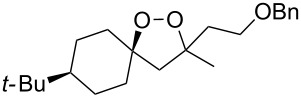	80	**122**	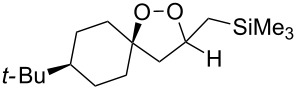	92
**114**	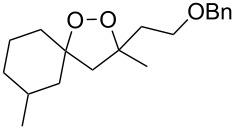	72	**123**	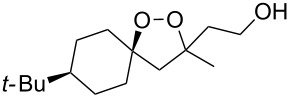	47
**115**	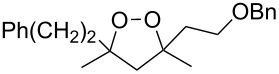	57	**124**	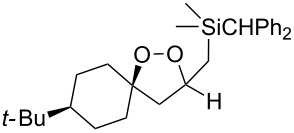	72
**116**	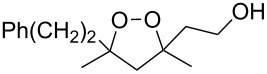	67	**125**	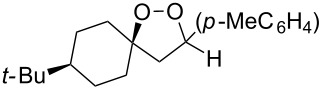	28
**117**	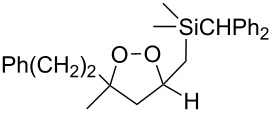	57	**126**	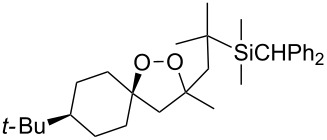	48
**118**	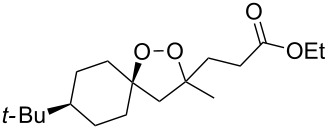	34	**127**	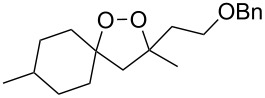	59
**119**	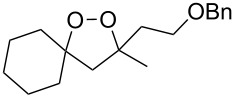	46	**128**	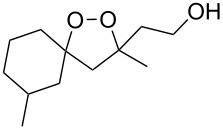	51
**120**	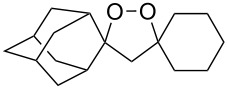	74	**129**	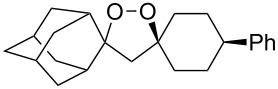	68
**121**	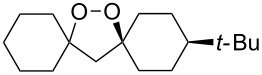	94	**130**	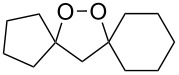	90

^a^Reagents and conditions*:* SnCl_4_ (1.0–2.0 equiv), alkene (1.0–3.0 equiv), CH_2_Cl_2_, from −78 °С to 25 °С, 2–24 h.

#### 1.4. Methods for the synthesis of 1,2-dioxolanes from hydrogen peroxide and hydroperoxides

This section deals with reactions, in which hydrogen peroxide or hydroperoxides are used for the construction of the five-membered peroxide ring. In all syntheses, the final (key) step involves the intramolecular cyclization of hydroperoxide with the attack on the electrophilic center (an activated double bond or a carbon atom of a keto or ester group).

**Scheme 30:**
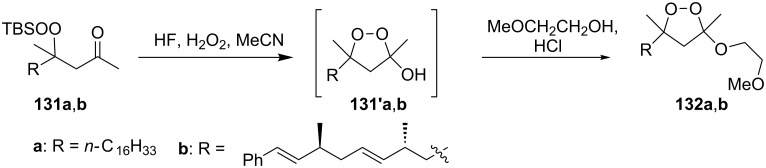
Desilylation of *tert*-butyldimethylsilylperoxy ketones **131a**,**b** followed by cyclization.

The desilylation of *tert*-butyldimethylsilylperoxy ketones **131a**,**b** with HF followed by cyclization and subsequent reaction with monomethylethylene glycol afforded dioxolanes **132a**,**b** in 75 and 88% yield, respectively. The intermediate hydroxydioxolanes **131'a**,**b** were used in the second step without isolation (Scheme 30) [260]. A series of analogues of plakinic acids were synthesized by the modification of the peroxyketal moiety of dioxolanes **132a** and **132b** [260].

**Scheme 31:**
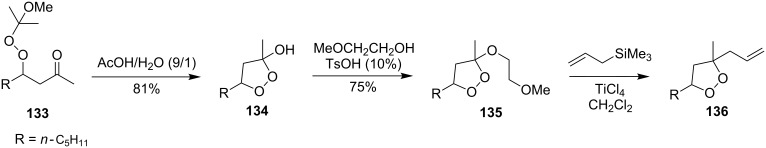
Deprotection of peroxide **133** followed by cyclization.

The monoperoxy ketal moiety of 4-(2-methoxypropan-2-ylperoxy)nonan-2-one (**133**) was used for the generation of the hydroperoxide group. The intramolecular cyclization afforded 3-methyl-5-pentyl-1,2-dioxolan-3-ol (**134**), which could be easily reacted with monomethylethylene glycol to form 3-(2-methoxyethoxy)-3-methyl-5-pentyl-1,2-dioxolane (**135**). Allylation of the latter produced 3-allyl-3-methyl-5-pentyl-1,2-dioxolane (**136**) in 47% yield (Scheme 31) [261].

**Scheme 32:**
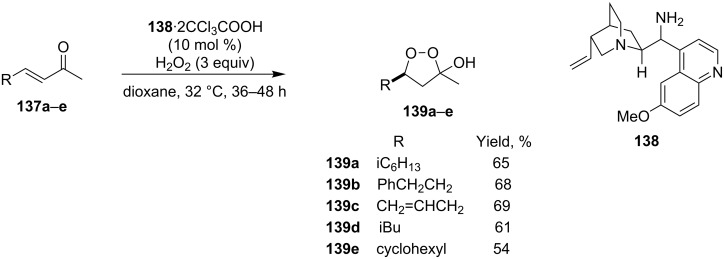
Asymmetric peroxidation of methyl vinyl ketones **137a–e**.

The asymmetric peroxidation of methyl vinyl ketones **137a–e** with 9-amino-9-deoxyepiquinine **138** and CCl_3_COOH afforded hydroxydioxolanes **139a–e** with high enantiomeric excess (ee 94–95%) (Scheme 32) [262].

**Scheme 33:**
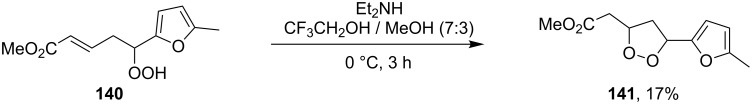
Et_2_NH-catalyzed intramolecular cyclization.

The Kobayashi synthesis of 1,2-dioxolanes represents an intramolecular version of the Michael reaction, in which the hydroperoxide group acts as the nucleophile. Generally, the reaction is performed in fluorinated alcohols (CF_3_CH_2_OH or (CF_3_)_2_CHOH) in the presence of diethylamine or, in some cases, of cesium hydroxide. Initially, the method was proposed for the synthesis of the 1,2-dioxane moiety (examples are considered in the corresponding section) [263]. However, it was shown that this method is also applicable to the preparation of structurally complex 1,2-dioxolanes, such as methyl 2-(5-(5-methylfuran-2-yl)-1,2-dioxolan-3-yl)acetate (**141**) from the furan derivative (*E*)-methyl 5-hydroperoxy-5-(5-methylfuran-2-yl)pent-2-enoate (**140**) (Scheme 33) [264].

**Scheme 34:**
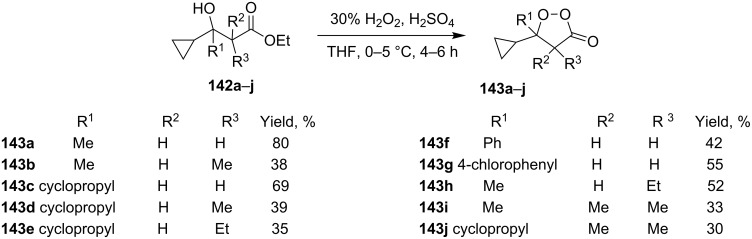
Synthesis of oxodioxolanes **143a–j**.

A simple method was developed for the synthesis of cyclopropane-containing oxodioxolanes **143a–j** and is based on the hydroperoxidation of tertiary alcohols **142a–j** in an acidic medium followed by cyclization of the intermediate hydroperoxides through the ester group (Scheme 34) [265].

This method allows for the use of a nonhazardous 30% hydrogen peroxide solution. However, the authors mentioned that structurally similar tertiary alcohols, without a cyclopropane substituent, are inert under the reported conditions.

**Scheme 35:**
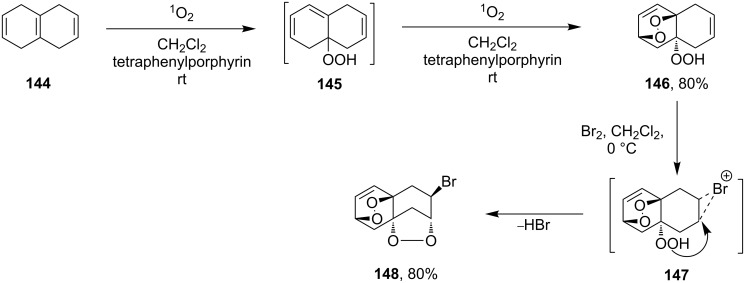
Haloperoxidation accompanied by intramolecular ring closure.

Haloperoxidation reaction that is accompanied by intramolecular ring closure represents another version of the cyclization reaction. For example, the reaction of bromine with unsaturated hydroperoxide **146** (produced by reaction of 1,4,5,8-tetrahydronaphthalene (**144**) with singlet oxygen via the formation of 4a-hydroperoxy-1,4,4a,5-tetrahydronapthalene (**145**) gives hydroperoxide-containing bromonium cation **147** as the intermediate, which undergoes cyclization to form 1,2-dioxolane-containing 7-bromo-4,5,10,11-tetraoxatetracyclo[7.2.2.1^3,6^.0^3,9^]tetradec-12-ene (**148**) (Scheme 35).

The cyclization occurs selectively because the hydroperoxide group in intermediate **147** attacks only one of two possible electrophilic carbon centers [266].

#### 1.5. 1,2-Dioxolane ring formation through oxidation of the allylic position

**Scheme 36:**
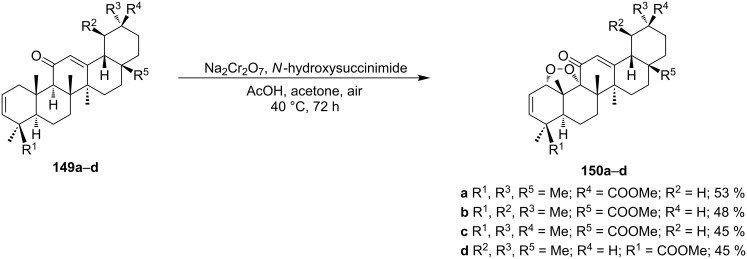
Oxidation of triterpenes **149a–d** with Na_2_Cr_2_O_7_/*N*-hydroxysuccinimide.

1,2-Dioxolane-containing compounds **150a–d** were synthesized by the oxidation of triterpenes **149a–d** with Na_2_Cr_2_O_7_/*N*-hydroxysuccinimide (Scheme 36). The resulting compounds exhibit antitumor activity comparable with that of betulinic acid [175–177].

#### 1.6. Structural modifications, in which the 1,2-dioxolane ring remains intact

**Scheme 37:**
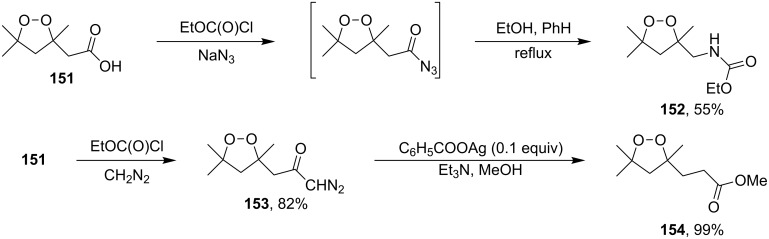
Curtius and Wolff rearrangements to form 1,2-dioxolane ring-retaining products.

The possibility of performing the Curtius and Wolff rearrangements to form 1,2-dioxolane ring-retaining products was exemplified by the synthesis of ethyl (3,5,5-trimethyl-1,2-dioxolan-3-yl)methylcarbamate (**152**) and methyl 3-(3,5,5-trimethyl-1,2-dioxolan-3-yl)propanoate (**154**) (through formation of stable diazodioxolane **153**) from 2-(3,5,5-trimethyl-1,2-dioxolan-3-yl)acetic acid (**151**) (Scheme 37) [267].

**Scheme 38:**
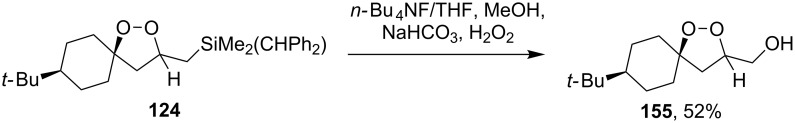
Oxidative desilylation of peroxide **124**.

Dioxolane **155** that contains a free hydroxy group was synthesized by the oxidative desilylation of silicon-containing peroxide **124** with *n*-Bu_4_NF and H_2_O_2_ (Scheme 38) [259].

**Scheme 39:**
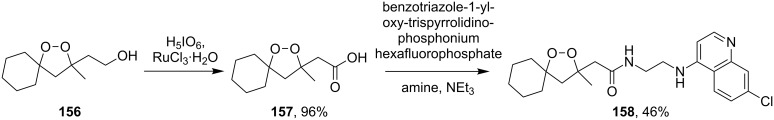
Synthesis of dioxolane **158**, a compound containing the aminoquinoline antimalarial pharmacophore.

Dioxolane **158** with the aminoquinoline antimalarial pharmacophore was synthesized in two steps by the oxidation of alcohol **156** with H_5_IO_6_/RuCl_3_ followed by amidation of the acid **157** (Scheme 39) [88]. It was shown that compound **158** exhibits antimalarial activity comparable with that of artemisinin [88].

**Scheme 40:**
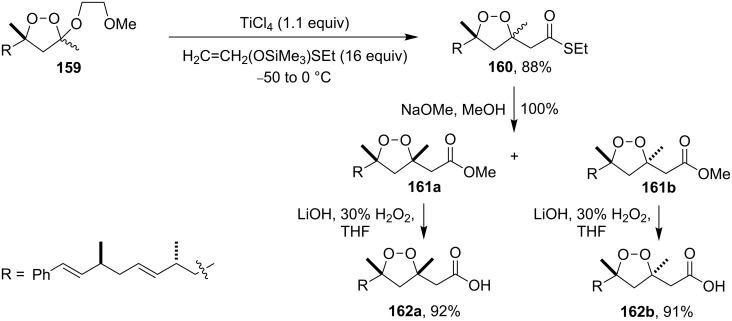
Diastereomers of plakinic acid A, **162a** and **162b**.

Plakinic acids belong to a large family of natural products, which were shown to be highly cytotoxic toward cancer cells and fungi. Diastereomers of plakinic acid A, **162a** and **162b** were synthesized starting from dioxolane ((*R*)-3-((2*R*,3*E*,6*S*,7*E*)-2,6-dimethyl-8-phenylocta-3,7-dienyl)-5-(2-methoxyethoxy)-3,5-dimethyl-1,2-dioxolane) (**159**) [260]. In the first step, dioxolane **159** was treated with (1-(ethylthio)vinyloxy)-trimethylsilane in the presence of TiCl_4_ to obtain *S*-ethyl 2-((*R*)-5-((2*R*,3*E*,6*S*,7*E*)-2,6-dimethyl-8-phenylocta-3,7-dienyl)-3,5-dimethyl-1,2-dioxolan-3-yl)-ethanethioate (**160**). The subsequent reaction with sodium methoxide in methanol produced the corresponding esters **161a** and **161b**, which were hydrolyzed to prepare the target plakinic acids (Scheme 40).

### 2. Synthesis of 1,2,4-trioxolanes (ozonides)

The currently most widely used methods for the synthesis of 1,2,4-trioxolanes are based on reactions of ozone with unsaturated compounds, such as the ozonolysis of alkenes, the cross-ozonolysis of alkenes with carbonyl compounds, and the cross-ozonolysis of О-alkylated oximes in the presence of carbonyl compounds (Griesbaum coozonolysis).

#### 2.1. Ozonolysis of alkenes

**Scheme 41:**
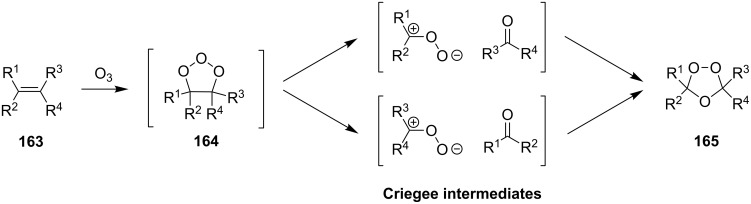
Ozonolysis of alkenes.

According to the mechanism proposed by R. Criegee [268–269] the ozonolysis of alkenes **163** involves several steps: the 1,3-dipolar cycloaddition of ozone to the double bond to form unstable 1,2,3-trioxolane **164** (so-called molozonide) that is followed by its decomposition to a peroxycarbenium ion and a carbonyl compound (Criegee intermediates). The 1,3-dipolar cycloaddition of the intermediates with each other form the 1,2,4-trioxolane **165** (Scheme 41, Table 11). Generally, the ozonolysis is performed in aprotic solvents at low temperatures and in some cases, on polymeric substrates. Since various compounds containing a С=С group are easily available, a wide range of functionalized 1,2,4-trioxolanes can be synthesized in moderate to high yields.

**Table 11:** Examples of 1,2,4-trioxolanes produced by the ozonolysis of alkenes.

Alkene **163**	Ozonolysis conditions	1,2,4-Trioxolane **165**	Yield, %	Reference

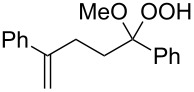	Et_2_O, −70 °C	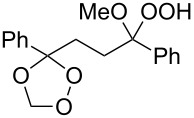	24	[270]
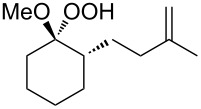	Et_2_O, −70 °C	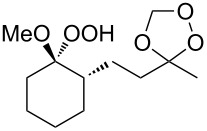	27	[270]
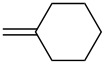	hexane, −78 °C	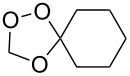	78	[256]
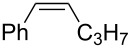	hexane, −78 °C	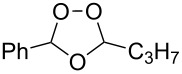	73	[256]
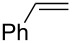	hexane, −78 °C	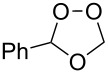	77	[256]
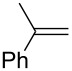	hexane, −78 °C	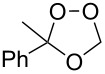	61	[256]
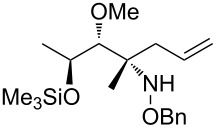	isooctane/CCl_4_, −78 °C, 1 h	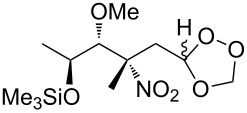	>82	[271]
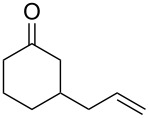	CH_2_Cl_2_, −78 °C	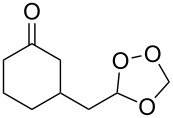	95	[272]
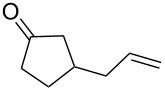	CH_2_Cl_2_, −78 °C	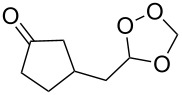	90	[272]
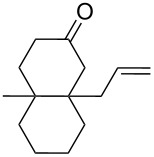	CH_2_Cl_2_, −78 °C	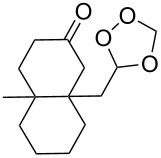	92	[272]
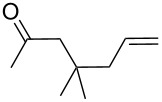	CH_2_Cl_2_, −78 °C	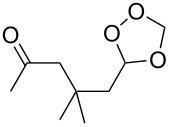	93	[272]
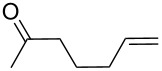	CH_2_Cl_2_, −78 °C	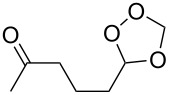	93	[272]
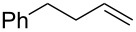	CH_2_Cl_2_, −78 °C	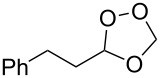	94	[272]
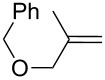	pentane, −78 °C	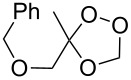	63	[272]
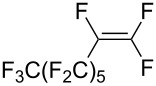	freon-113, 15–20 °C, 2 h	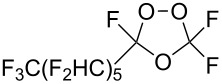	The yield was not determined	[273–274]
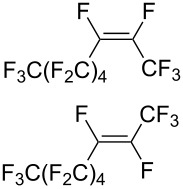	freon-113, 15–20 °C, 2 h	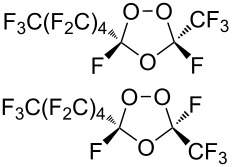	The yield was not determined	[273]
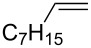	CH_2_Cl_2_, −78 °C	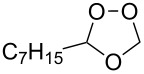	96	[275]
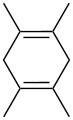	polymer-based, −78 °C, 8 h	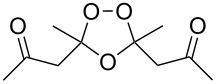	23	[276]
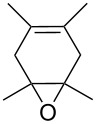	polymer-based, −78 °C, 3 h	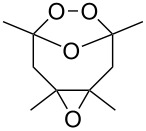	38	[276]
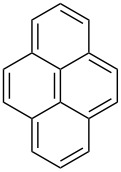	CH_2_Cl_2_, −70 °C	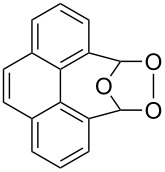	48	[277]
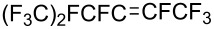	without solvent, −133 to −43 °C	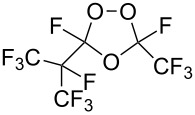	100	[278]
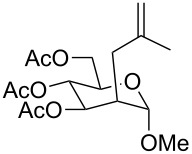	1) CH_2_Cl_2_, −78 °C, 15 min. 2) Me_2_S, rt, 6 h	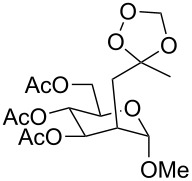	71	[279]
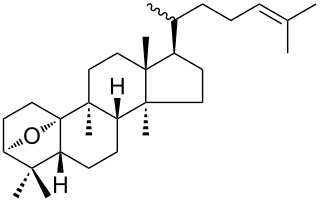	hexane, −78 °C, 30 min	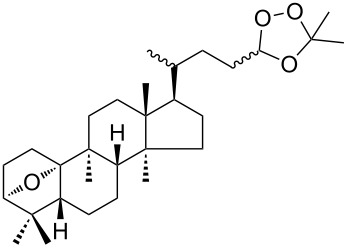	6	[280]
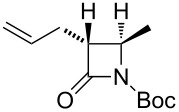	CH_2_Cl_2_, −78 °C, 20 min	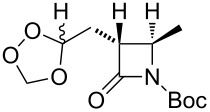	The yield was not determined	[281]
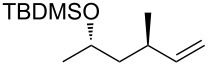	CH_2_Cl_2_, −78 °C, 2 h	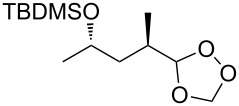	>97	[282]
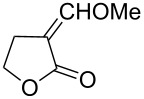	CDCl_3_, −65 °C	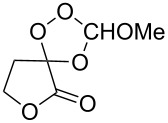	88	[283]
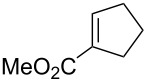	CFCl_3_, −70 °C	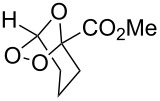	100	[283]
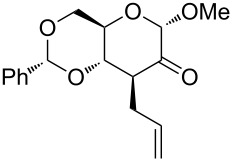	CH_2_Cl_2_, −78 °C, 1 h	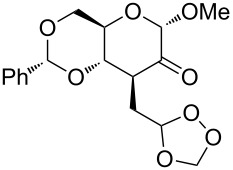	85	[284]
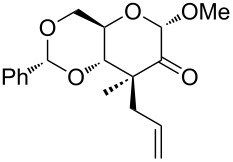	CH_2_Cl_2_, −78 °C, 1 h	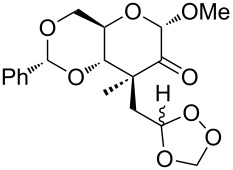	70	[285]
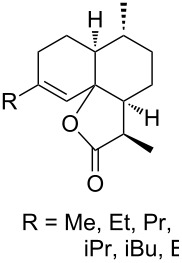	pentane, −78 °C	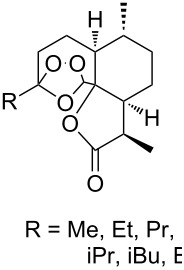	10-30	[148–152]
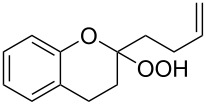	Et_2_O/CH_3_OH, −78 °C	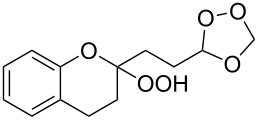	12	[254]
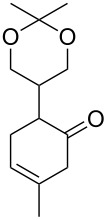	CH_2_Cl_2_, −78 °C	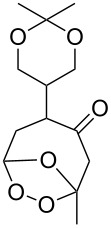	92	[286]
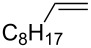	CH_2_Cl_2_, 0 °C	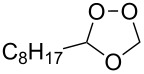	95	[287–288]
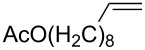	H_2_O/CH_2_Cl_2_, 0 °C	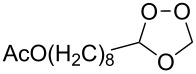	72	[289]
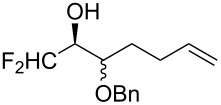	1) SiO_2_, −78 °C; 2) Me_2_S, MeOH	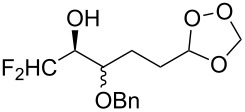	30	[290]

#### 2.2. Cross-ozonolysis of alkenes with carbonyl compounds

**Scheme 42:**
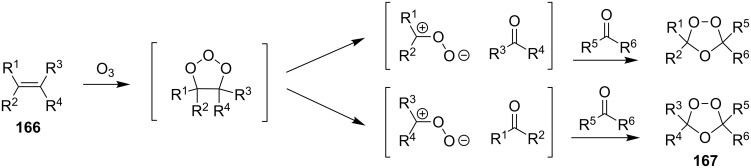
Cross-ozonolysis of alkenes **166** with carbonyl compounds.

The peroxycarbenium ion produced by the decomposition of 1,2,3-trioxolane (molozonide) can react with externally introduced carbonyl compounds to form the corresponding 1,2,4-trioxolanes. The pathway of decomposition of 1,2,3-trioxolanes is determined by the structure of the starting alkene **166**. In some cases, a high selectivity of the formation of cross-ozonolysis products 1,2,4-trioxolanes (ozonides) **167**, can be achieved (Scheme 42, Table 12).

**Table 12:** Examples of 1,2,4-trioxolanes synthesized by the cross-ozonolysis of alkenes in the presence of carbonyl compounds.

Alkene **166**	Carbonyl compound	Ozonolysis conditions	1,2,4-Trioxolane **167**	Yield, %	Reference

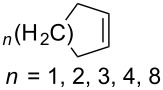	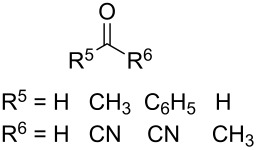	CH_2_Cl_2_, −78 °C	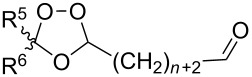	17–74	[291]
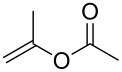	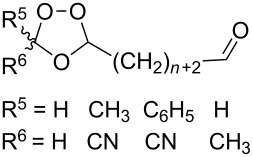	CH_2_Cl_2_, −78 °C	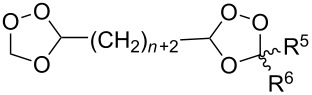	9–57	[291]
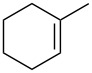	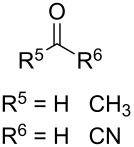	CH_2_Cl_2_, −78 °C	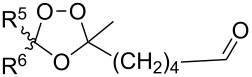	50 42	[291]
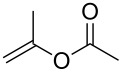	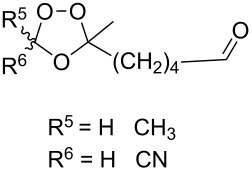	CH_2_Cl_2_, −78 °C	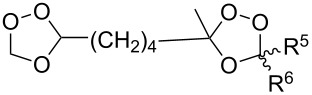	38 32	[291]
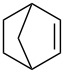	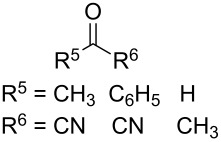	CH_2_Cl_2_, −78 °C	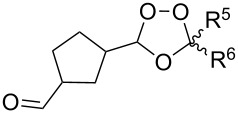	18–48	[291]
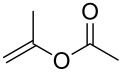	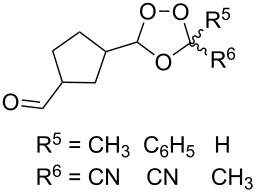	CH_2_Cl_2_, −78 °C	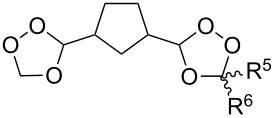	25–37	[291]
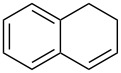	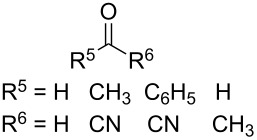	CH_2_Cl_2_, −78 °C	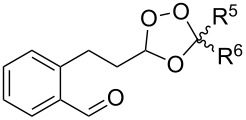	63–80	[291]
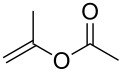	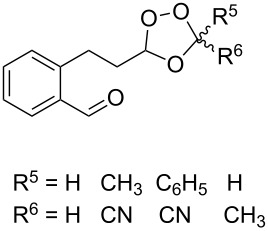	CH_2_Cl_2_, −78 °C	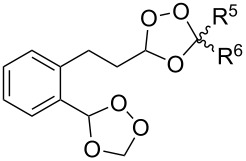	55–77	[291]
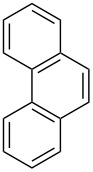	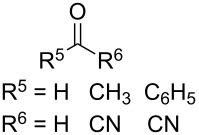	CH_2_Cl_2_, −78 °C	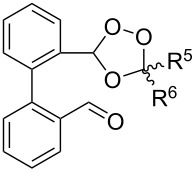	49–74	[291]
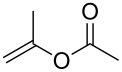	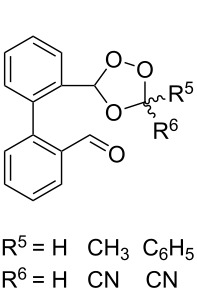	CH_2_Cl_2_, −78 °C	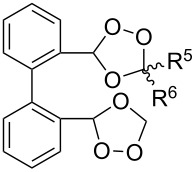	63–66	[291]
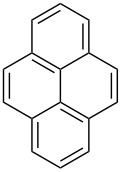	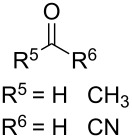	CH_2_Cl_2_, −78 °C	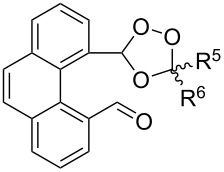	52 62	[291]
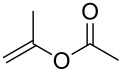	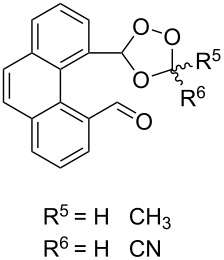	CH_2_Cl_2_, −78 °C	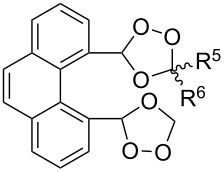	41 46	[291]
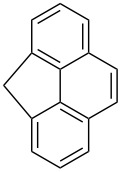	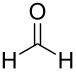	CH_2_Cl_2_, −78 °C	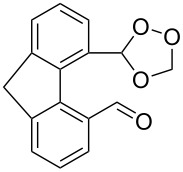	82	[291]
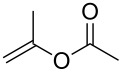	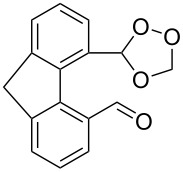	CH_2_Cl_2_, −78 °C	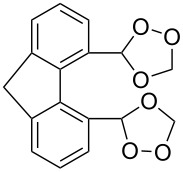	26	[291]
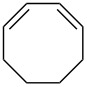	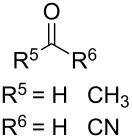	CH_2_Cl_2_, −78 °C	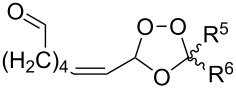	58 53	[292]
			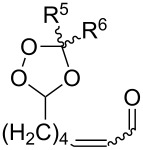	21 21	[292]
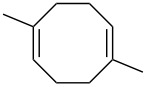	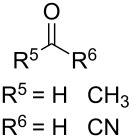	CH_2_Cl_2_, −78 °C	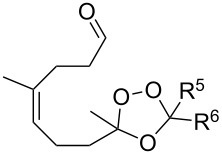	23 25	[292]
			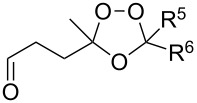	68 60	[292]
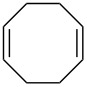	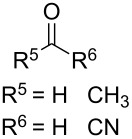	CH_2_Cl_2_, −78 °C	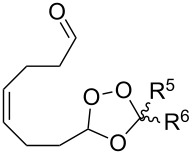	30 25	[292]
			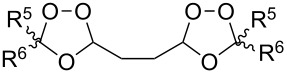	15 14	[292]
			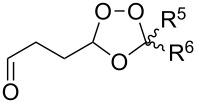	25 27	[292]
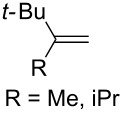	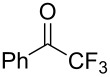	Et_2_O, −70 °C	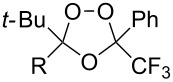	60 56	[293]
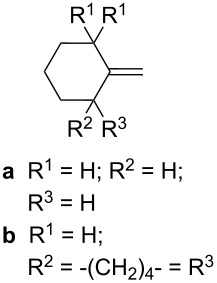	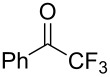	Et_2_O, −70 °C	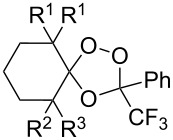	59 65	[293]
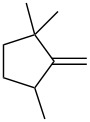	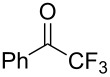	Et_2_O, −70 °C	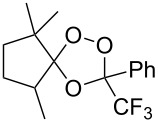	59	[293]

**Scheme 43:**
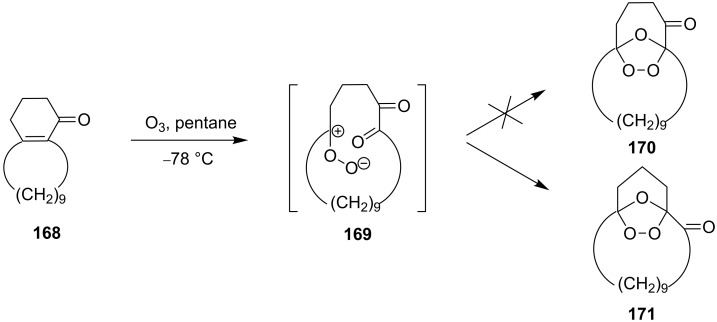
Ozonolysis of the bicyclic cyclohexenone **168**.

For the ozonolysis of the bicyclic cyclohexenone, 2,3,4,5,6,7,8,9,10,11,12,13,14,15-tetradecahydro-1*H*-benzo[13]annulen-1-one (**168**), two reaction pathways can be proposed through intermediate **169** to form ozonides **170** and **171**. It appeared that the reaction gave only 16,17,18-trioxatricyclo[10.3.2.11,12]octadecan-2-one **171** as two isomers, with the *anti* isomer in 60% and the *syn* isomer in 10% yield (Scheme 43) [294]. The structures of these compounds were established by X-ray diffraction [294].

**Scheme 44:**
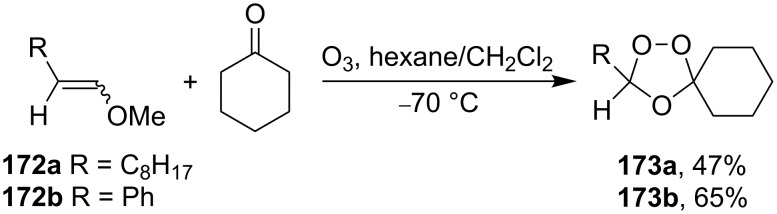
Cross-ozonolysis of enol ethers **172a**,**b** with cyclohexanone.

The cross-ozonolysis of enol ethers **172a**,**b** with cyclohexanone enabled the synthesis of 1,2,4-trioxolanes **173a**,**b** containing the easily oxidizable C–H fragment in the third position (Scheme 44) [256].

#### 2.3. Cross-ozonolysis of O-alkyl oximes in the presence of carbonyl compounds (Griesbaum co-ozonolysis)

**Scheme 45:**
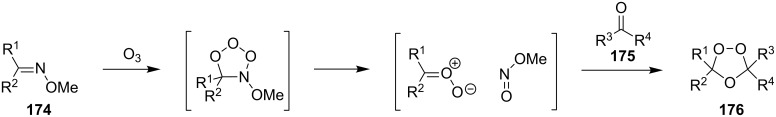
Griesbaum co-ozonolysis.

In 1995, K. Griesbaum and co-workers reported a new type of cross-ozonolysis [295]. This method enables the synthesis of tetrasubstituted ozonides **176** by an ozone-mediated reaction of О-alkyl oximes **174** with ketones **175** (Scheme 45, Table 13). The selective synthesis of ozonides has attracted great interest because it allows the preparation of compounds exhibiting high antiparasitic activity.

**Table 13:** Examples of ozonides (1,2,4-trioxolanes) synthesized by the Griesbaum method.

Oxime **174**	Ketone **175**	Ozonolysis conditions	1,2,4-Trioxolane **176**	Yield, %	Ref.

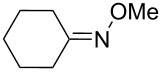	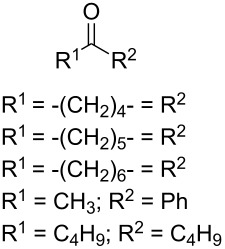	hexane, −78 °C	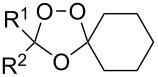	47–67	[256]
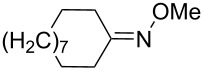	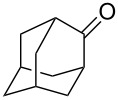	pentane, CH_2_Cl_2_, 0 °C	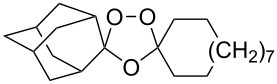	54	[91]
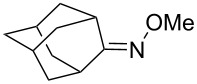	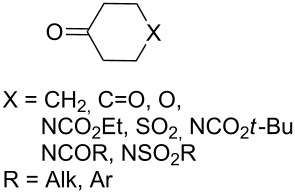	pentane, CH_2_Cl_2_, 0 °C	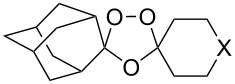	10–75	[91,94] [95,296]
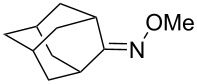	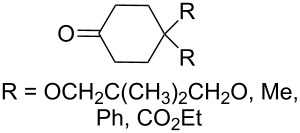	pentane, CH_2_Cl_2_, 0 °C	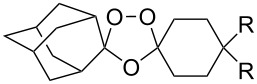	23–50	[91–93]
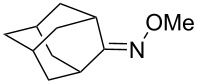	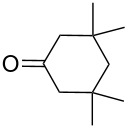	pentane, 0 °C	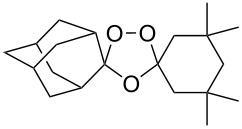	48	[92–93]
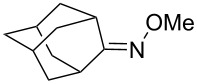	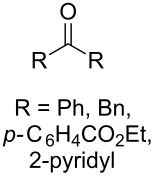	pentane, CH_2_Cl_2_, 0 °C	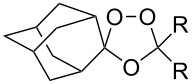	32–58	[91–93]
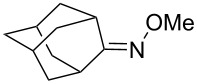	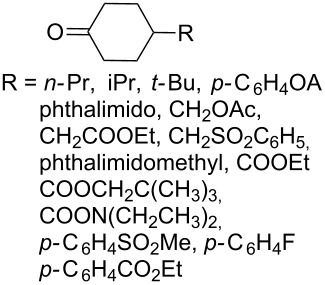	pentane, CH_2_Cl_2_, 0 °C	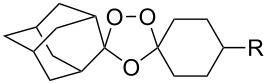	20–70	[91–93,96] [97,297]
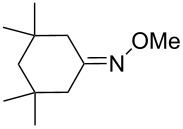	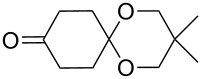	pentane, 0 °C	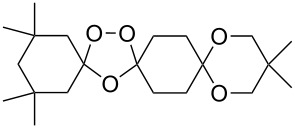	38	[91]
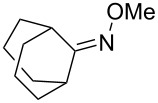	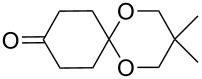	pentane, 0 °C	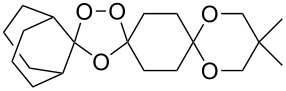	41	[91]
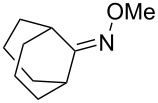	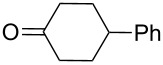	pentane, CH_2_Cl_2_, 0 °C	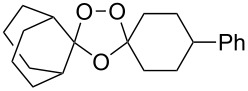	33	[91]
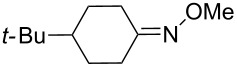	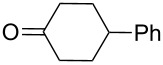	hexane, CH_2_Cl_2_, 0 °C	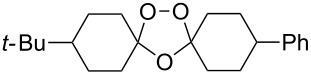	17	[91]
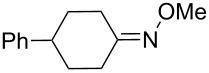	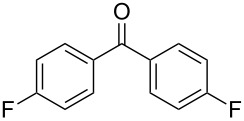	pentane, CH_2_Cl_2_, 0 °C	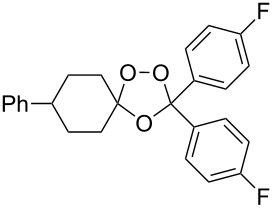	27	[91]
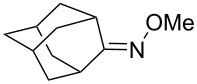	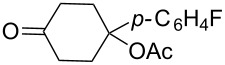	pentane, CH_2_Cl_2_, 0 °C	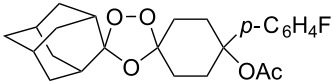	53	[92–93]
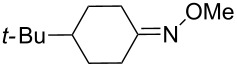	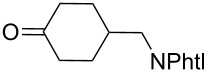	pentane, CH_2_Cl_2_, 0 °C	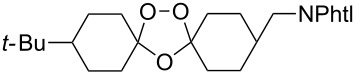	n.d.^a^	[96–97]
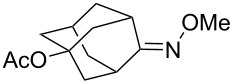	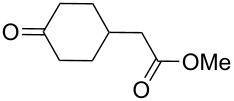	cyclohexane CH_2_Cl_2_, 0 °C	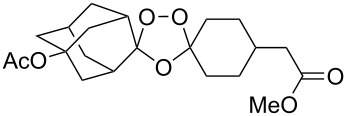	30	[298]
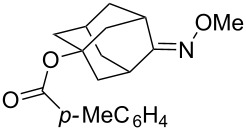	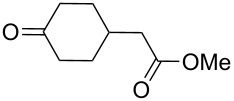	cyclohexane CH_2_Cl_2_, 0 °C	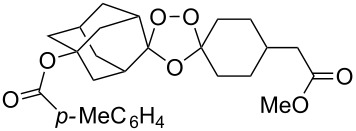	54	[298]
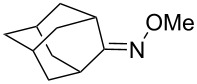	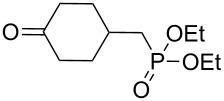	cyclohexane, CH_2_Cl_2_, 0 °C	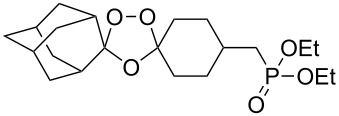	78	[258]

^a^Yield was not determined

The Griesbaum method is widely applicable and allows the selective synthesis of symmetrical and unsymmetrical 1,2,4-trioxolanes, which are not accessible by direct ozonolysis of alkenes or the cross-ozonolysis of alkenes or enol ethers in the presence of carbonyl compounds. In addition, this method does not need tetrasubstituted alkenes or enol ethers as starting materials, which are difficult to prepare. Taking into account a wide range of commercially available ketones, it can be concluded that this is the most universal method for the synthesis of 1,2,4-trioxolanes in terms of selectivity and structural diversity of the final products.

#### 2.4. Other methods for the synthesis of 1,2,4-trioxolanes

**Scheme 46:**
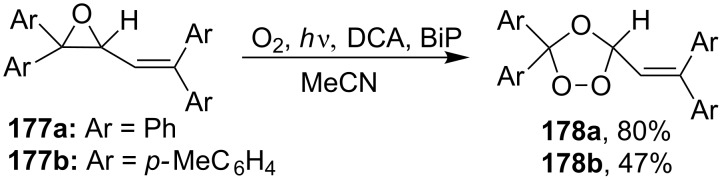
Reactions of aryloxiranes **177a**,**b** with oxygen.

The reactions of aryloxiranes **177a**,**b** with oxygen in the presence of 9,10-dicyanoanthracene (DCA) and biphenyl (BiP) under irradiation produced 1,2,4-trioxolanes **178a** and **178b** (Scheme 46). It should be noted that the oxirane moiety is oxidized rather than the double bond in these reactions [299].

**Scheme 47:**
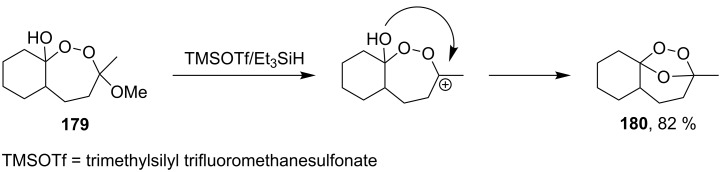
Intramolecular formation of 1,2,4-trioxolane **180**.

This unusual result was obtained upon treatment of the hydroxydioxepane, 3-methoxy-3-methyloctahydro-3*H*-benzo[*c*][1,2]dioxepin-9a-ol (**179**) with TMSOTf/Et_3_SiH. Thus, the peroxide moiety was not reduced with Et_3_SiH, and the reaction produced the bicyclic peroxide, 1-methyl-10,11,12-trioxatricyclo[7.2.1.0^4,9^]dodecane (**180**) containing the 1,2,4-trioxolane moiety, as the major product (Scheme 47) [270].

**Scheme 48:**
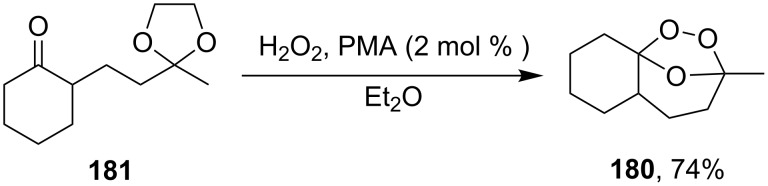
Formation of 1,2,4-trioxolane **180** by the reaction of 1,5-ketoacetal **181** with H_2_O_2_.

The same bicyclic peroxide **180** was synthesized in good yield by the reaction of 2-(2-(2-methyl-1,3-dioxolan-2-yl)ethyl)cyclohexanone (**181**) with hydrogen peroxide in the presence of phosphomolybdic acid (PMA) (Scheme 48) [300].

#### 2.5. Structural modifications, in which 1,2,4-trioxolane ring remains intact

**Scheme 49:**
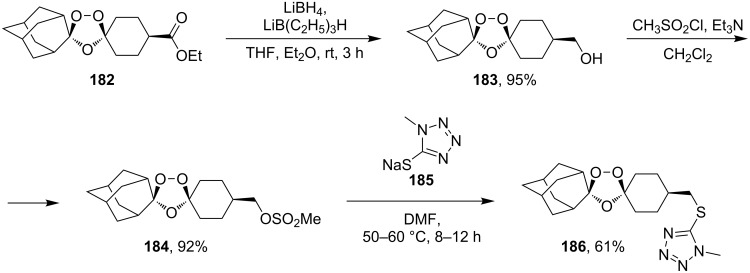
1,2,4-Trioxolane **186** with tetrazole fragment.

Scheme 49 shows possible modifications of substituents at the ozonide ring by the reduction of the ester group in *cis*-adamantane-2-spiro-3’-8’-ethoxycarbonyl-1’,2’,4’-trioxa-spiro[4.5]decane **182** to form the alcohol *cis*-adamantane-2-spiro-3’-8’-hydroxymethyl-1’,2’,4’-trioxaspiro[4.5]decane **183.** The latter was mesylated to **184** (*cis*-adamantane-2-spiro-3’-8’-methanesulfonylmethyl-1’,2’,4’-trioxa-spiro[4.5]decane), and used in the reaction with sodium 1-methyl-1*H*-tetrazole-5-thiolate **185** for the synthesis of *cis*-adamantane-2-spiro-3’-8’-[[(1’-methyl-1’*H*-tetrazol-5’-yl)thio]methyl]-1’,2’,4’-trioxaspiro[4.5]decane **186** through nucleophilic substitution of the mesyl group by the thio group of tetrazole **185** (Scheme 49) [297].

**Scheme 50:**
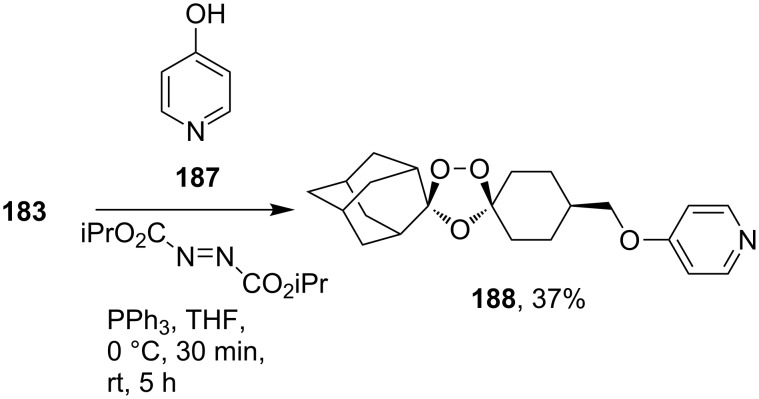
1,2,4-Trioxolane **188** with a pyridine fragment.

Ozonide **188** was synthesized by Mitsunobu reaction of alcohol **183** with pyridin-4-ol (**187**) (Scheme 50) [93]. It should be emphasized that this method can be applied in spite of the use of triphenylphosphine, which is a strong reducing agent for peroxides.

**Scheme 51:**
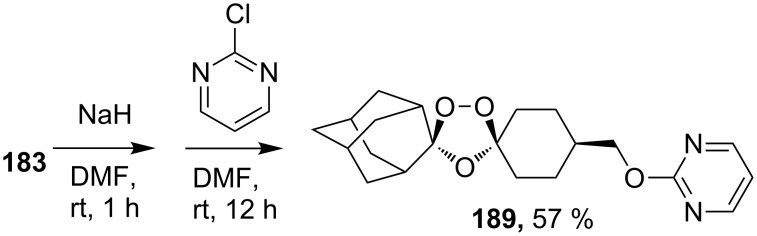
1,2,4-Trioxolane **189** with pyrimidine fragment.

The alkylation of the sodium salt of alcohol **183** with 2-chloropyrimidine in dimethylformamide gave ozonide **189** (Scheme 51). In this reaction, neither sodium hydride nor sodium salt **183** cleave the ozonide ring to a substantial degree. The resulting 1,2,4-trioxolanes **188** and **189** exhibit high in vitro antimalarial activity comparable with that of artemisinin and in vivo even higher activity than that of artemisinin [93].

**Scheme 52:**
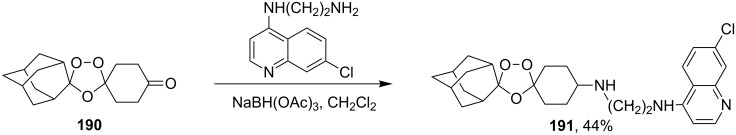
Synthesis of aminoquinoline-containing 1,2,4-trioxalane **191**.

Aminoquinoline-containing 1,2,4-trioxalane **191** was synthesized by reductive amination of adamantane-2-spiro-3’-8’-oxo-1’,2’,4’-trioxaspiro[4.5]-decane **190** (Scheme 52). Ozonide **191** is an example of a combination of two known antiparasitic pharmacophores, viz. a peroxide and an aminoquinoline moiety [296].

**Scheme 53:**
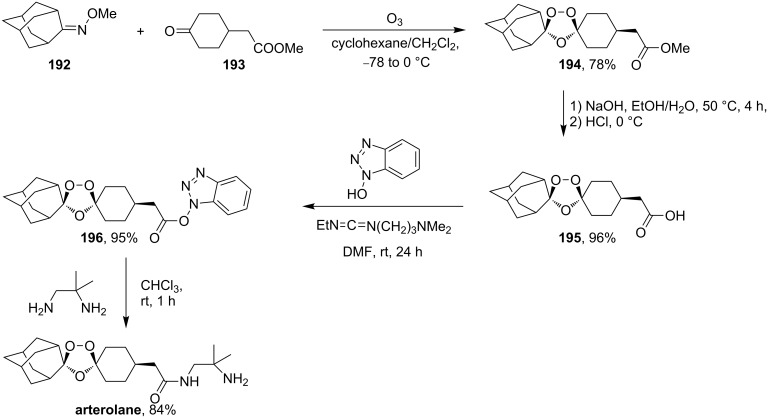
Synthesis of arterolane.

Arterolane is a fully synthetic 1,2,4-trioxalane, also known as OZ277. It has high antimalarial activity and is currently in the final stage of clinical trials. As drug, this compound is used in combination with piperaquine. The synthesis of arterolane is based on the Griesbaum coozonolysis of a mixture of adamantan-2-one *O*-methyloxime (**192**) and 4-carbomethoxycyclohexanone **193** to form *cis*-adamantane-2-spiro-3’-8’-methoxycarbonylmethyl-1’,2’,4’-trioxaspiro[4.5]decane **194**. The latter is hydrolyzed to *cis*-adamantane-2-spiro-3’-8’-carboxymethyl-1’,2’,4’-trioxaspiro[4.5]decane **195**, followed by mild amidation with the formation of the intermediate ozonide **196** that on treatment with 2-methylpropane-1,2-diamine finally gives the target compound (Scheme 53). The in vitro and in vivo studies showed that arterolane is more active against causative agents of malaria than artemisinin, chloroquine, and mefloquine [77–78,81].

#### 3. Synthesis of 1,2-dioxanes

Modern approaches to the synthesis of 1,2-dioxanes are based on reactions with singlet oxygen, the oxidative coupling of carbonyl compounds and alkenes in the presence of manganese and cerium salts, the co-oxidation of alkenes and thiols with oxygen, the Isayama–Mukaiyama peroxidation, the Kobayashi cyclization of hydroperoxides, the reaction of 1,4-diketones with hydrogen peroxide, the intramolecular nucleophilic substitution by the hydroperoxide group, the cyclization with participation of halogenonium ion donors, acid-mediated rearrangements of peroxides, the palladium-catalyzed cyclization of compounds with С=С and –О–О– groups, and reactions with the participation of peroxycarbenium ions.

#### 3.1. Methods for the synthesis of 1,2-dioxanes using singlet oxygen

**Scheme 54:**
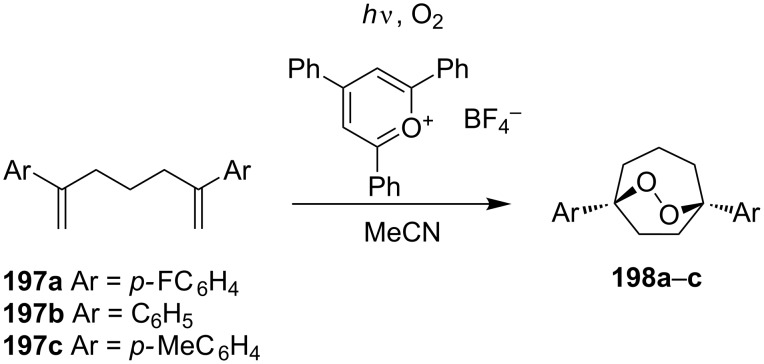
Oxidation of diarylheptadienes **197a–c** with singlet oxygen.

The oxidation of diarylheptadienes **197a–c** with singlet oxygen in acetonitrile afforded bicyclic peroxides **198a–c** in 33–58% yields. 2,4,6-Triphenylpyrylium tetrafluoroborate was used as the sensitizer for singlet oxygen generation (Scheme 54) [301].

It was found that tris(bipyrazyl)ruthenium(II) [(Ru(bpz)_3_(PF_6_)_2_] is an excellent photocatalyst for the synthesis of 1,2-dioxanes by aerobic photooxygenation of α,ω-dienes [302].

**Scheme 55:**
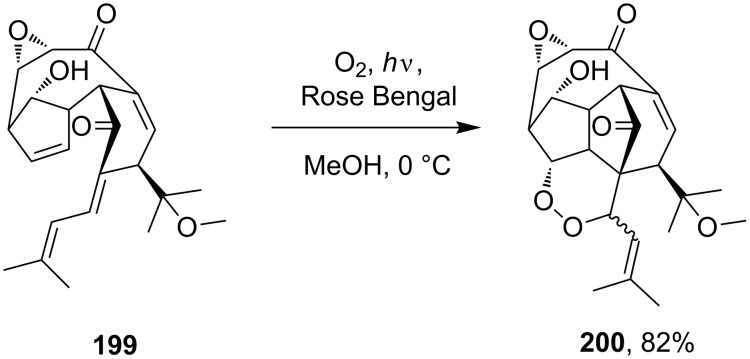
Synthesis of hexacyclinol peroxide **200**.

The addition of singlet oxygen to substrate **199** occurs in the last step of the synthesis of natural hexacyclinol peroxide **200** (Scheme 55) [303].

**Scheme 56:**
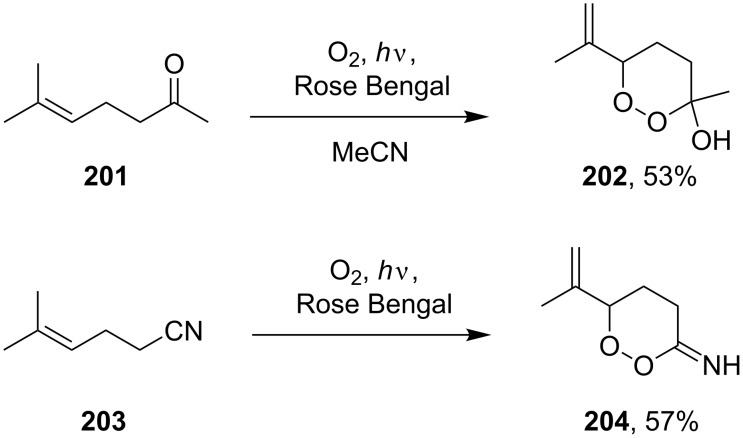
Oxidation of enone **201** and enenitrile **203** with singlet oxygen.

The reactions of 6-methylhept-5-en-2-one (**201**) and 5-methylhex-4-enenitrile (**203**) with singlet oxygen produced 1,2-dioxanes, 3-methyl-6-(prop-1-en-2-yl)-1,2-dioxan-3-ol (**202**) and 6-(prop-1-en-2-yl)-1,2-dioxane-3-imine (**204**), containing the hydroxy and imine groups, respectively (Scheme 56) [304].

#### 3.2. Oxidative coupling of carbonyl compounds and alkenes in the presence of manganese or cerium salts

**Scheme 57:**
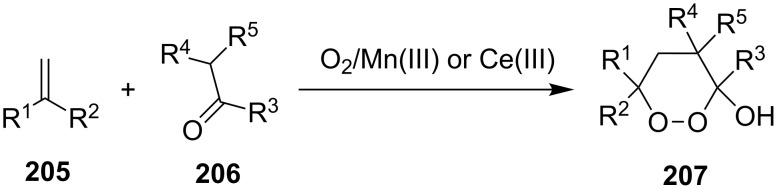
Synthesis of 1,2-dioxanes **207** by oxidative coupling of carbonyl compounds **206** and alkenes **205**.

The synthesis of 1,2-dioxanes **207** is based on the addition of alkene **205** and oxygen to carbonyl compound **206** via the intermediate formation of carbon-centered peroxide radicals. The reaction occurs in the presence of catalytic amounts of manganese or cerium salts, which are involved in a redox cycle. It is assumed that the oxidation of β-dicarbonyl compounds proceeds through a formation of an enol-containing complex with a metal ion (Scheme 57, Table 14).

**Table 14:** Examples of 1,2-dioxanes **207** synthesized by oxidative coupling of carbonyl compounds **206** and alkenes **205**.

Alkene **205**	Carbonyl compound **206**	Reaction conditions	1,2-Dioxane **207**	Yield, %	Ref.

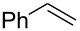	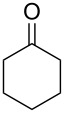	Mn(OAc)_2_, O_2_, AcOH, 80 °C, 10 h	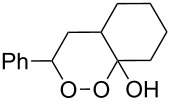	67	[305]
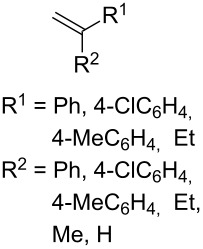	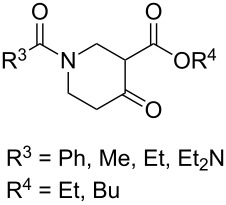	Mn(OAc)_3_, air, AcOH, 23 °C, 0.5–24 h	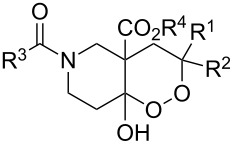	20–84	[306]
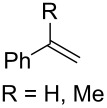	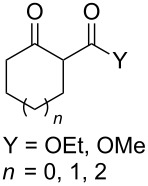	CeCl_3_×7H_2_O, air, iPrOH, rt, 16 h	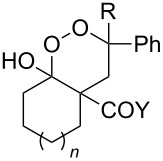	42–87	[307]
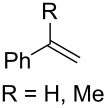	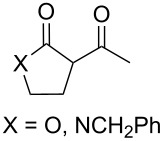	CeCl_3_×7H_2_O, air, iPrOH, rt, 16 h	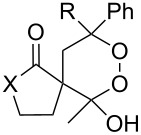	5–73	[307]
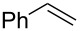	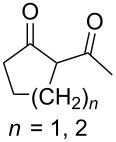	CeCl_3_×7H_2_O, air, iPrOH, rt, 14–16 h	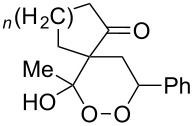	19 (*n* = 1), 33 (*n* = 2)	[308]
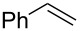	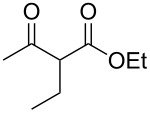	CeCl_3_×7H_2_O, air, iPrOH, rt, 14–16 h	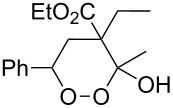	18	[308]
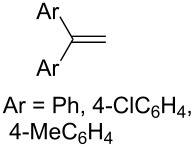	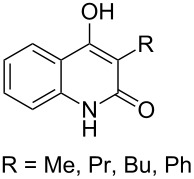	Mn(OAc)_3_, air, AcOH, 23 °C, 15–18 h	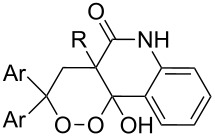	22–89	[309]
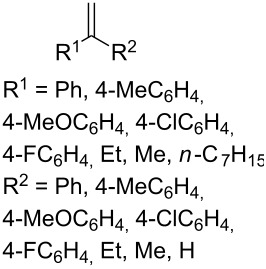	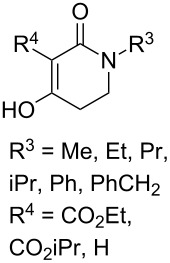	Mn(OAc)_3_, air, AcOH, 23 °C, 9–18 h	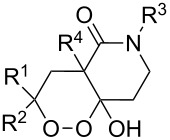	17–99	[310]
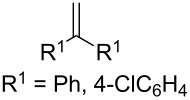	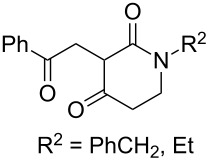	Mn(OAc)_3_, air, AcOH, reflux, 10 min	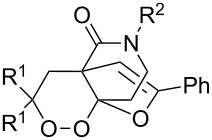	9–11	[311]
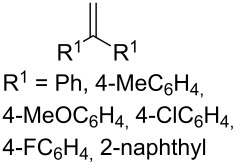	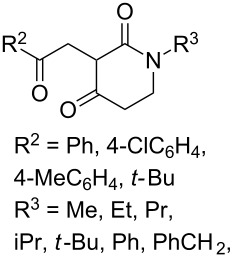	Mn(OAc)_3_, air, AcOH, rt, 3.5–7.5 h	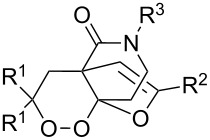	8–75	[312]

#### 3.3. Oxidation of 1,5-dienes in the presence of thiols

**Scheme 58:**
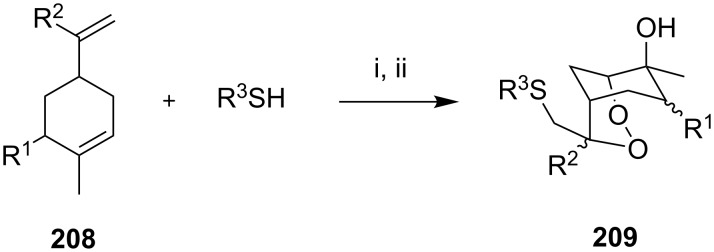
1,2-Dioxanes **209** synthesis by co-oxidation of 1,5-dienes **208** and thiols.

The co-oxidation of 1,4-dienes and thiols (thiol–olefin co-oxygenation, TOCO reaction) was described for the first time by Beckwith and Wagner as a method for the synthesis of sulfur-containing 1,2-dioxolanes [313–314]. More recently, it has been shown that under similar conditions, the oxidation of 1,5-dienes **208** affords the corresponding sulfur-containing 1,2-dioxanes **209**. The reaction proceeds under oxygen atmosphere in the presence of azobisisobutyronitrile (AIBN) or di*tert*-butyl peroxalate (DBPO) as radical initiators. The resulting unstable hydroperoxides are reduced with triphenylphosphine to hydroxy derivatives **209** (Scheme 58, Table 15).

**Table 15:** Examples of 1,2-dioxanes synthesized by co-oxidation of 1,5-dienes and thiols.

Diene **208**	Thiol	Reaction conditions	1,2-Dioxane **209**	Yield, %	Reference

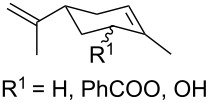	R^3^SH R^3^ = Ph, 4-FC_6_H_4_, *n*-Bu, *t*-Bu, MeO_2_CCH_2_, cyclohexyl, Ph_3_C	1) O_2_, DBPO, benzene/heptane, rt, 10 h or O_2_, AIBN, *h*ν, MeCN, 4 °C, 10 h 2) Ph_3_P, CH_2_Cl_2_, 0–5 °C, 2 h, rt, 1 h	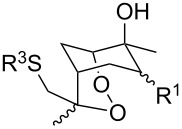	6–54	[107,179,315]
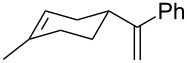	PhSH 4-ClC_6_H_4_SH	1) O_2_, AIBN, *h*ν, MeCN, 0 °C, 2 h 2) Ph_3_P, CH_2_Cl_2_, 0 °C to rt	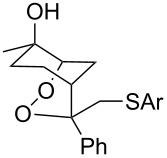	70 (Ar = Ph), 21 (Ar = 4-ClC_6_H_4_)	[102,316]

**Scheme 59:**
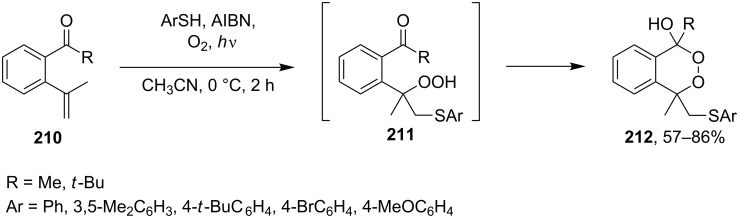
Synthesis of bicyclic 1,2-dioxanes **212** with aryl substituents.

The oxidation of acetophenones **210** produces bicyclic 1,2-dioxanes **212** (Scheme 59). It is hypothesized that the reaction gives hydroperoxide **211** as the intermediate, that undergoes rapid cyclization to form the target 1,2-dioxane **212** [317].

#### 3.4. Synthesis of 1,2-dioxanes by the Isayama–Mukaiyama method

**Scheme 60:**
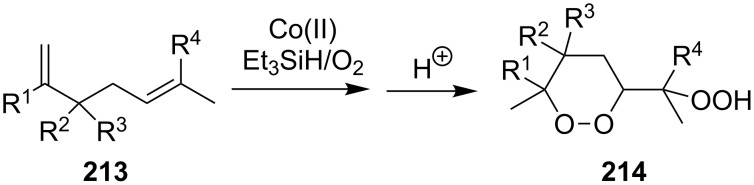
Isayama–Mukaiyama peroxysilylation of 1,5-dienes **213** followed by desilylation under acidic conditions.

The Isayama–Mukaiyama peroxysilylation of 1,5-dienes **213** followed by desilylation under acidic conditions gives hydroperoxide-containing 1,2-dioxanes **214** (Scheme 60, Table 16).

**Table 16:** Synthesis of 1,2-dioxanes by the Isayama–Mukaiyama method.

1,5-Diene **213**	Reaction conditions^a^	1,2-Dioxane **214**	Yield, %	Reference

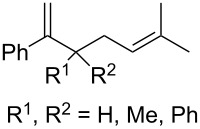	1) Co(modp)_2_, O_2_, Et_3_SiH, ClCH_2_CH_2_Cl, 2–6 h 2) HCl/MeOH	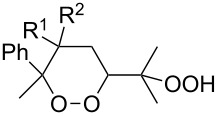	13–64	[249]
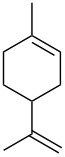	1) Co(modp)_2_, O_2_, Et_3_SiH, ClCH_2_CH_2_Cl, 1 h 2) HCl/MeOH	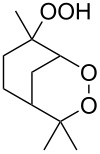	22	[318–319]

^a^modp = 1-morpholino-5,5-dimethyl-1,2,4-hexanetrionate.

**Scheme 61:**
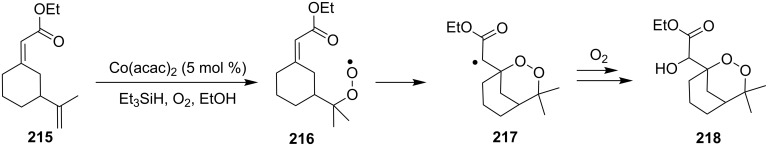
Synthesis of bicycle **218** with an 1,2-dioxane ring.

The oxidation of (*Z*)-ethyl 2-(3-(prop-1-en-2-yl)cyclohexylidene)acetate (**215**) gives ethyl 2-(4,4-dimethyl-2,3-dioxabicyclo[3.3.1]nonan-1-yl)-2-hydroxyacetate (**218**) in 29% yield. The oxidative reaction proceeds presumably with formation of an O-centered radical **216**, then a C-centered radical **217** and the latter adds oxygen and is reduced to the hydroxy derivative of 1,2-dioxane **218** (Scheme 61) [318].

**Scheme 62:**
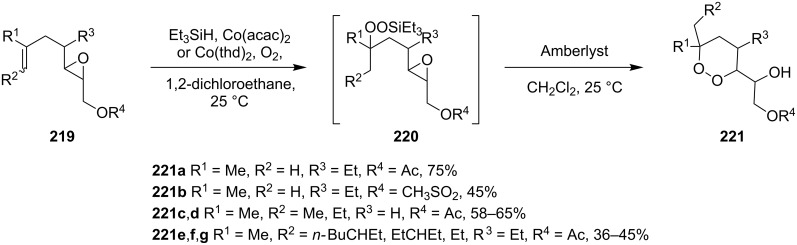
Intramolecular cyclization with an oxirane-ring opening.

**Scheme 63:**
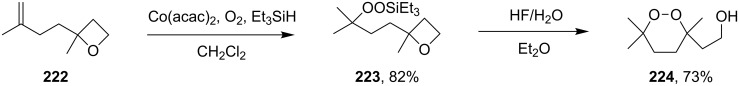
Inramolecular cyclization with the oxetane-ring opening.

An alternative synthesis of a 1,2-dioxane by the Isayama–Mukaiyama method includes the following sequence of reactions: peroxysilylation, desilylation, and recyclization accompanied by a ring opening of oxirane or oxetane (Scheme 62 and Scheme 63).

Cobalt(II) acetylacetonate (acac) or bis-2,2,6,6-tetramethylheptane-3,5-dienoate (thd) were used as the catalyst for the peroxidation of **219**. The cyclization of the intermediate peroxide **220** was performed with Amberlyst-15 ion-exchange resin. This approach was used in the multistep synthesis of the natural endoperoxide 9,10-dihydroplakortin, which exhibits antimalarial and anticancer activities as do its structural analogues [320–321].

2-(3,6,6-Trimethyl-1,2-dioxan-3-yl)ethanol (**224**) was synthesized in a similar way starting with the peroxidation of 2-methyl-2-(3-methylbut-3-enyl)oxetane (**222**), followed by oxetane-ring opening in triethyl(2-methyl-4-(2-methyloxetan-2-yl)butan-2-ylperoxy)silane (**223**) (Scheme 63) [250].

**Scheme 64:**
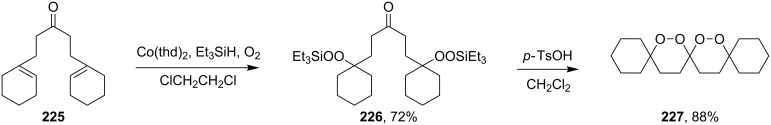
Intramolecular cyclization with the attack on a keto group.

Dioxanes can also be synthesized by inramolecular cyclizations with the attack on a keto group. The peroxysilylation of the unsaturated ketone 1,5-dicyclohexenylpentan-3-one (**225**), with the Co(thd)_2_/Et_3_SiH/O_2_ system produced 1,5-bis(1-(triethylsilylperoxy)cyclohexyl)pentan-3-one (**226**), which underwent cyclization in the presence of *p*-toluenesulfonic acid to give the spiro-fused 7,8,10,11-tetraoxatrispiro[5.2.2.5.2.2]henicosane **227** (Scheme 64) [252].

#### 3.5. Synthesis of 1,2-dioxanes by the Kobayashi method

**Scheme 65:**
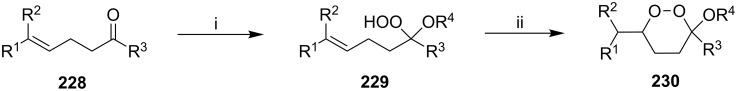
Peroxidation of the carbonyl group in unsaturated ketones **228** followed by cyclization of hydroperoxy acetals **229**.

The synthesis is based on the peroxidation of the carbonyl group of unsaturated ketones **228** with the urea–hydrogen peroxide complex followed by a Michael cyclization of the hydroperoxy acetals **229** under basic conditions. This method is suitable for the efficient synthesis of functionalized 1,2-dioxanes **230** in moderate to high yields (Scheme 65, Table 17). In early studies, scandium(III) triflate was used as the catalyst for the hydroperoxidation of ketones with the H_2_O_2_–H_2_NCONH_2_ complex. More recently, it was shown that in some cases, cheaper catalysts such as *p*-toluenesulfonic and 10-camphorsulfonic acid can be used for this purpose (Table 17).

**Table 17:** Examples of 1,2-dioxanes synthesized by the Kobayashi method.

Unsaturated ketone **228**	Reaction conditions	1,2-Dioxane **230**	Yield, %	Reference

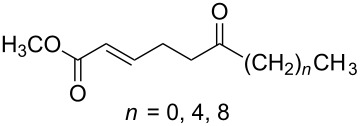	1) H_2_O_2_^.^H_2_NCONH_2_, Sc(OTf)_3_, MeOH 2) Et_2_NH, CF_3_CH_2_OH, 0 °C, 2 d	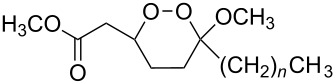	1) 67–83 2) 60–72	[322–323]
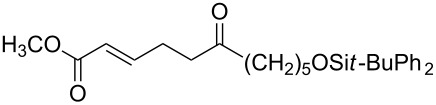	1) H_2_O_2_^.^H_2_NCONH_2_, Sc(OTf)_3_, MeOH 2) Et_2_NH, CF_3_CH_2_OH 3) HF, pyridine, THF	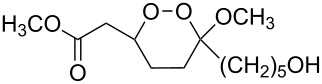	1) 52 2) 87 3) 100	[324]
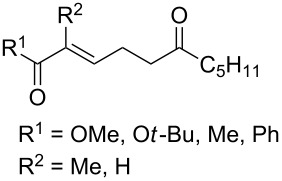	1) H_2_O_2_^.^H_2_NCONH_2_, Sc(OTf)_3_, MeOH 2) Et_2_NH, CF_3_CH_2_OH	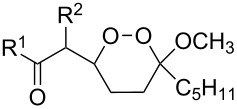	8–38	[104–106]
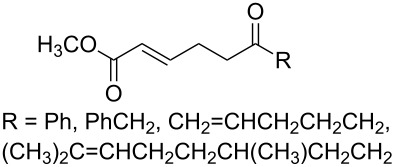	1) H_2_O_2_^.^H_2_NCONH_2_, Sc(OTf)_3_, MeOH 2) Et_2_NH, CF_3_CH_2_OH	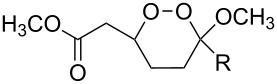	28–58	[104–106]
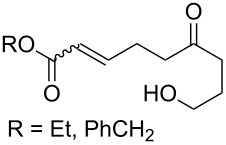 Intermediate product **229** has the following structure: 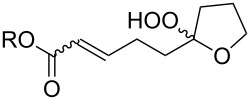	1) H_2_O_2_^.^H_2_NCONH_2_, *p-*TsOH, MeOH, rt, 20 h 2) Et_2_NH, CF_3_CH_2_OH, rt, 24 h	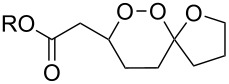	1) 54–82 2) 52	[325–327]
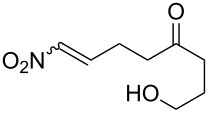	H_2_O_2_–H_2_NCONH_2_, *p-*TsOH, 1,2-dimethoxyethane, rt, 11 h	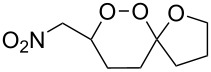	35	[325–327]
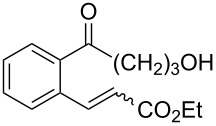 Intermediate product **229** has the following structure: 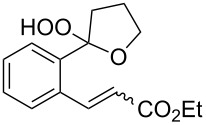	1) H_2_O_2_^.^H_2_NCONH_2_, 10-camphorsulfonic acid, 1,2-dimethoxyethane, rt, 18 h 2) Et_2_NH, CF_3_CH_2_OH, rt, 2 h	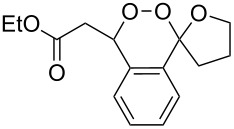	1) 86 2) 54	[325–327]
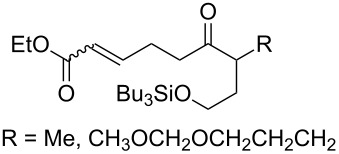	1) H_2_O_2_^.^H_2_NCONH_2_, *p-*TsOH, EtOH, rt, 10 h 2) Et_2_NH, CF_3_CH_2_OH, rt, 12 h	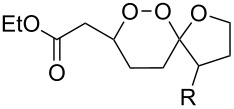	1) 72–80 2) 40–52	[328]
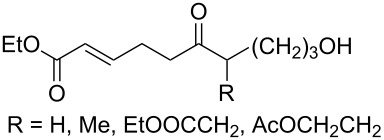	1) H_2_O_2_^.^H_2_NCONH_2_, *p-*TsOH, EtOH, rt, 12 h 2) Et_2_NH, CF_3_CH_2_OH, rt	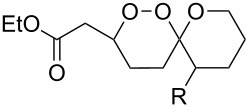	1) 70–93 2) 42–65	[328]

**Scheme 66:**
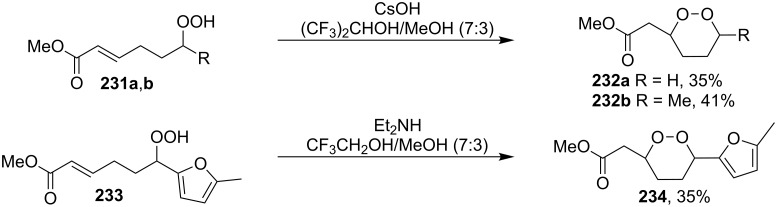
CsOH and Et_2_NH-catalyzed cyclization.

It was found that cesium hydroxide can be used as a base for the cyclization to give **232** and **234**. Compared to Scheme 65, the method is suitable for the cyclization of hydroperoxides **231** and **233**, which are no ketone derivatives (Scheme 66) [264]. Et_3_N in MeOH can also be used as catalyst for this type of cyclization [263].

**Scheme 67:**
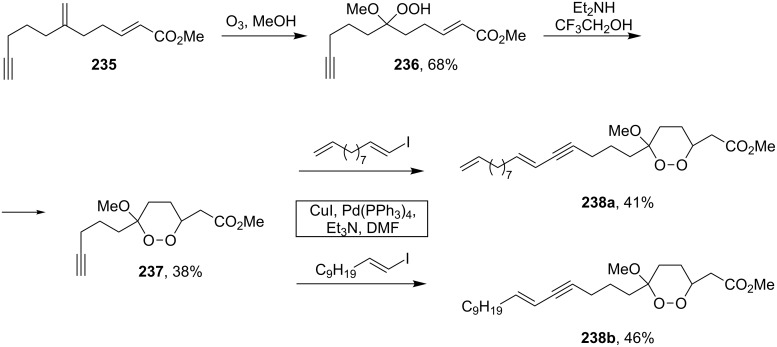
Preparation of peroxyplakoric acid methyl ethers A and D.

The synthesis of peroxyplakoric acid methyl ethers A and D **238a** and **238b**, which are natural peroxides isolated from marine sponges exhibiting fungicidal and antitumor activities [329–330] is an interesting example of the synthesis of complex structures. The polyunsaturated compound (*E*)-methyl 6-methyleneundec-2-en-10-ynoate (**235**) was subjected to ozonolysis to obtain methoxyhydroperoxide, (*E*)-methyl 6-hydroperoxy-6-methoxyundec-2-en-10-ynoate (**236**), whose cyclization afforded methyl 2-(6-methoxy-6-(pent-4-inyl)-1,2-dioxan-3-yl)acetate (**237**), in which the triple bond is easily modified by palladium-catalyzed cross-coupling reactions to form the target 1,2-dioxanes **238a**,**b** (Scheme 67).

**Scheme 68:**
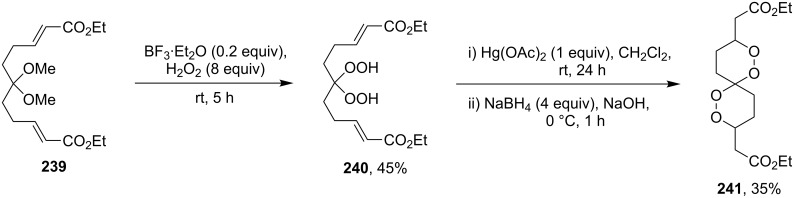
Hg(OAc)_2_ in 1,2-dioxane synthesis.

Initially, an attempt was made to synthesize diethyl 2,2'-(1,2,7,8-tetraoxaspiro[5.5]undecane-3,9-diyl)diacetate (**241**) by cyclization of (2*E*,9*E*)-diethyl 6,6-dihydroperoxyundeca-2,9-dienedioate bis(hydroperoxide) (**240**) (the bishydroperoxidation product of (2*E*,9*E*)-diethyl 6,6-dimethoxyundeca-2,9-dienedioate (**239**)) with Et_2_NH in CF_3_CH_2_OH. However, these attempts failed. Spiroperoxide **241** was prepared in satisfactory yield by reaction of **240** with the use of mercury (II) acetate (Scheme 68) [331]. The intermediate mercury-containing peroxide produced by the cyclization of bis(hydroperoxide) **240** was reduced with NaBH_4_ in an alkaline medium [331].

#### 3.6. Synthesis of 1,2-dioxanes from 1,4-dicarbonyl compounds

**Scheme 69:**
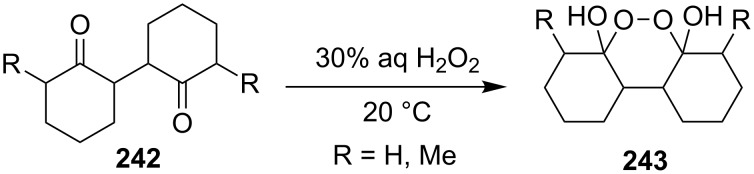
Reaction of 1,4-diketones **242** with hydrogen peroxide.

The reaction of 1,4-diketones **242** (cyclohexanone derivatives) with hydrogen peroxide in a neutral medium produced 3,6-dihydroxydioxanes **243** albeit without reported yields (Scheme 69). The resulting compounds exhibit a broad spectrum of antiparasitic activities against causative agents of malaria, trypanosomiasis, and leishmaniasis [208–212].

#### 3.7. Methods for the synthesis of 1,2-dioxanes from hydroperoxides

**Scheme 70:**
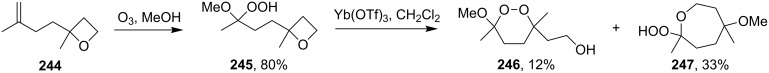
Inramolecular cyclization with oxetane-ring opening.

Compounds containing a С=С group and an oxygen-containing ring are convenient starting materials for the synthesis of cyclic peroxides [250–252,332]. For example, the ozonolysis of the double bond in the oxetane-containing compound, 2-methyl-2-(3-methylbut-3-enyl)oxetane (**244**) afforded 2-(3-hydroperoxy-3-methoxybutyl)-2-methyloxetane (**245**), which underwent recyclization in the presence of ytterbium triflate to give 2-(6-methoxy-3,6-dimethyl-1,2-dioxan-3-yl)ethanol (**246**) along with the seven-membered compound 2-hydroperoxy-5-methoxy-2,5-dimethyloxepane (**247**) (Scheme 70) [250].

**Scheme 71:**
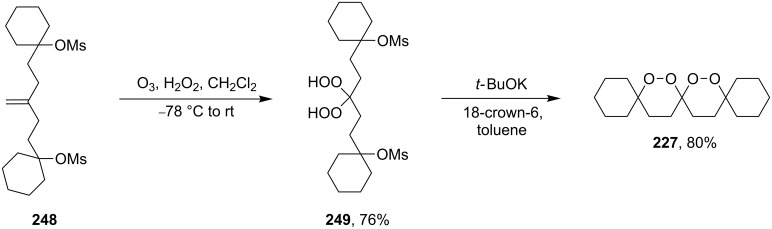
Inramolecular cyclization with MsO fragment substitution.

Spirodioxane **227**, whose synthesis by the Isayama–Mukaiyama method was described above (Scheme 64), could also be synthesized via the ozonolysis of alkene **248** in the presence of hydrogen peroxide followed by the cyclization of bis(hydroperoxide) **249** with potassium *tert*-butoxide (Scheme 71) [252].

**Scheme 72:**
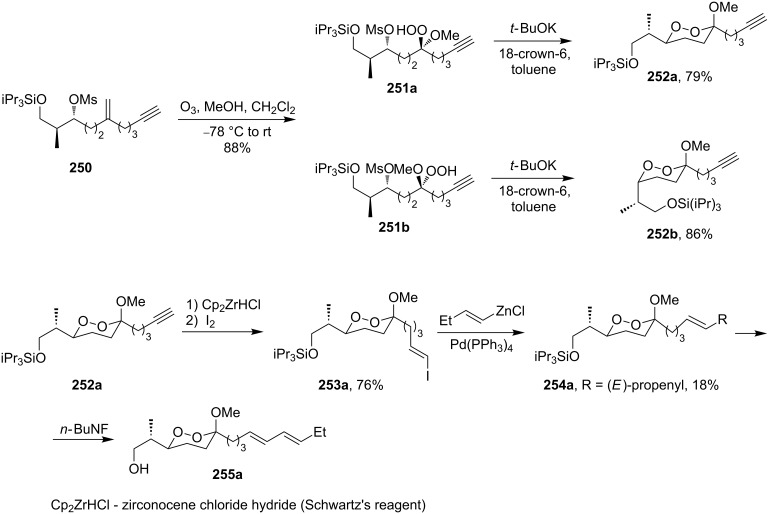
Synthesis of 1,2-dioxane **255a**, a structurally similar compound to natural peroxyplakoric acids.

An approach to the cyclization based on an intramolecular nucleophilic substitution was used also for the synthesis of diastereomers of dioxanes **252a**,**b** containing triple bonds. Hydroperoxides **251a**,**b** that were synthesized by the ozonolysis of **250** were treated with potassium *tert*-butoxide. One of the diastereomers, **252a**, was then modified first via the stereoselective hydrozirconation and iodination to **253a** and then by the Negishi cross coupling to produce silylated product **254a**, which was desilylated to obtain alcohol **255a** (Scheme 72). 1,2-Dioxane **255a** is structurally similar to natural peroxyplakoric acids having fungicidal and antimalarial activities [332].

#### 3.8. Use of halonium ions in the cyclization

This approach to the synthesis of 1,2-dioxane rings is based on the intramolecular cyclization of hydroperoxides containing a C=C group. In the first step, the addition of a halonium ion to the double bond results in the formation of a carbocation, which is subjected to the intramolecular attack of the hydroperoxide group.

**Scheme 73:**
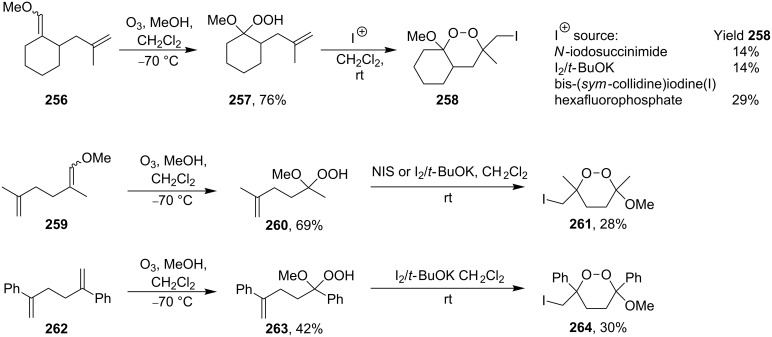
Synthesis of 1,2-dioxanes based on the intramolecular cyclization of hydroperoxides containing C=C groups.

The treatment of unsaturated monoperoxyketals **257**, **260**, and **263** (prepared by ozonolysis of **256**, **259**, and **262** in methanol, respectively) with such donors of halonium ions such as *N*-iodosuccinimide (NIS), I_2_/*t*-BuOK, or bis(sym-collidine)iodonium hexafluorophosphate gave iodine-containing 1,2-dioxanes **258**, **261**, and **264**, in moderated yields (Scheme 73) [333]. It should be noted that attempts to synthesize related peroxides with *N*-bromosuccinimide failed [333].

**Scheme 74:**
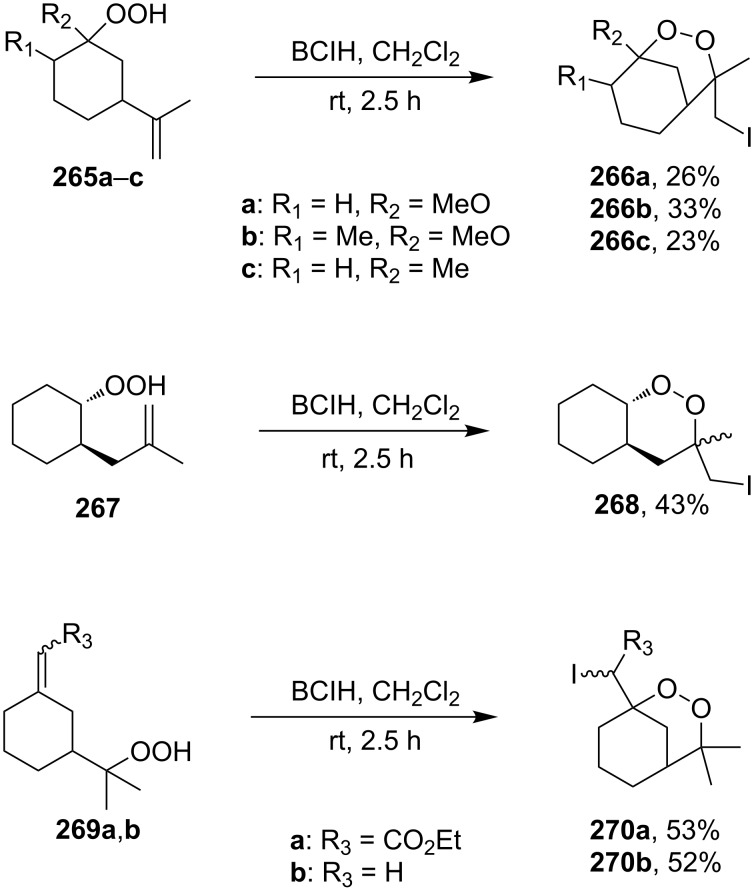
Use of BCIH in the intramolecular cyclization.

In the studies [334–335] iodine-containing 1,2-dioxanes **266a–c**, **268**, and **270a**,**b** were synthesized from the corresponding hydroperoxyalkenes **265a–c**, **267**, and **269a**,**b** with bis(sym-collidine)iodonium hexaflulorophosphate (BCIH) in the cyclization step (Scheme 74).

#### 3.9. Pd(II)-catalyzed cyclization

**Scheme 75:**
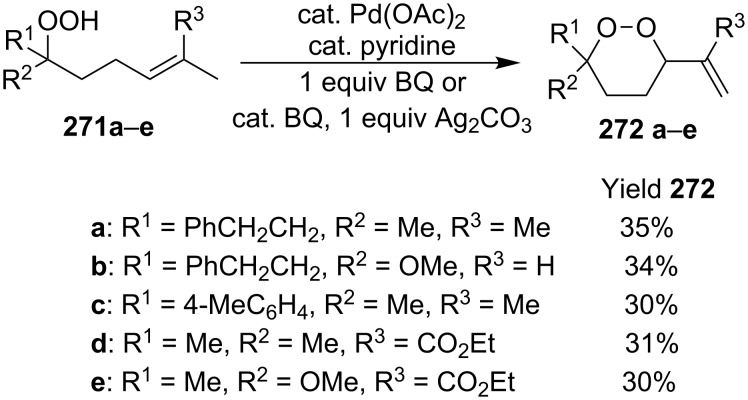
Palladium-catalyzed cyclization of δ-unsaturated hydroperoxides **271a–e**.

The palladium-catalyzed cyclization of δ-unsaturated hydroperoxides **271** represents a new route to 1,2-dioxane cyclic compounds **272** (Scheme 75). The cyclization was performed in toluene, 1,4-dioxane, or 1,2-dichloroethane at 80 °C for 3 h in the presence of *p*-benzoquinone or silver carbonate as the oxidizing agent for Pd(0) that was formed in the catalytic cycle. To the best of our knowledge, this method is the first example of a palladium acetate-catalyzed synthesis of cyclic peroxides [336].

#### 3.10. Acid-mediated cyclizations of peroxides

**Scheme 76:**
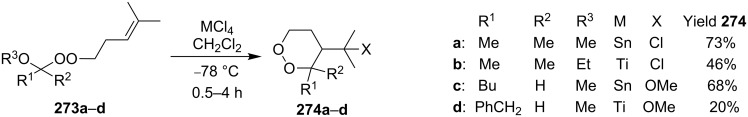
Intramolecular cyclization of unsaturated peroxyacetals **273a–d**.

The intramolecular cyclization of unsaturated peroxyacetals **273a–d** in the presence of TiCl_4_ or SnCl_4_ occurs via formation of peroxycarbenium ions to give methoxy- and chlorine-containing dioxanes **274a–d** as the reaction products (Scheme 76) [257].

**Scheme 77:**
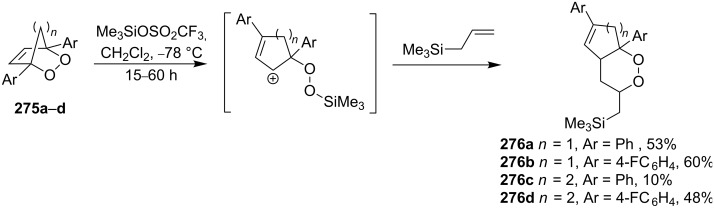
Allyltrimethylsilane in the synthesis of 1,2-dioxanes **276a–d**.

The treatment of endoperoxides **275a–d** with allyltrimethylsilane in the presence of catalytic amounts of trimethylsilyl triflate or SnCl_4_ gave bicyclic 1,2-dioxanes **276a–d** (Scheme 77) [337].

**Scheme 78:**
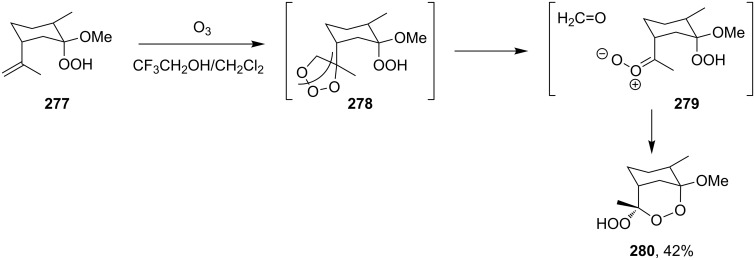
Intramolecular cyclization using the electrophilic center of the peroxycarbenium ion **279**.

The electrophilic center of the peroxycarbenium ion produced by the decomposition of molozonide can be trapped by the hydroperoxide group of the molecule. This type of cyclization was used as the basis for the synthesis of hydroperoxide-containing 1,2-dioxanes. The ozonolysis of 1-hydroperoxy-1-methoxy-2-methyl-5-(prop-1-en-2-yl)cyclohexane (**277**) in a trifluoroethanol/dichloromethane mixture through formation of molozonide **278** and peroxycarbenium ion **279** finally afforded (6*S*)-6-hydroperoxy-1-methoxy-2,6-dimethyl-7,8-dioxabicyclo[3.3.1]nonane (**280**) (Scheme 78) [334]. The intramolecular cyclization of intermediate **279** is only possible if the hydroperoxide group is in a particular spatial arrangement [334].

**Scheme 79:**
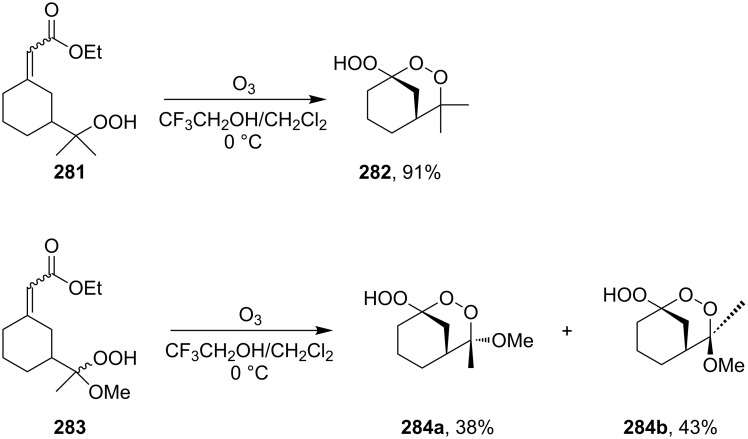
Synthesis of bicyclic 1,2-dioxanes.

Under these conditions, ethyl 2-(3-(2-hydroperoxypropan-2-yl)cyclohexylidene)acetate hydroperoxide (**281**) and ethyl 2-(3-(1-hydroperoxy-1-methoxyethyl)cyclohexylidene)acetate hydroperoxide (**283**) react to form dioxanes, (1*S*,5*S*)-1-hydroperoxy-4,4-dimethyl-2,3-dioxabicyclo[3.3.1]nonane (**282**), (1*S*,4*S*,5*S*)-1-hydroperoxy-4-methoxy-4-methyl-2,3-dioxabicyclo[3.3.1]nonane (**284a**), and (1*S*,4*R*,5*S*)-1-hydroperoxy-4-methoxy-4-methyl-2,3-dioxabicyclo[3.3.1]nonane (**284b**) (Scheme 79) [338].

**Scheme 80:**
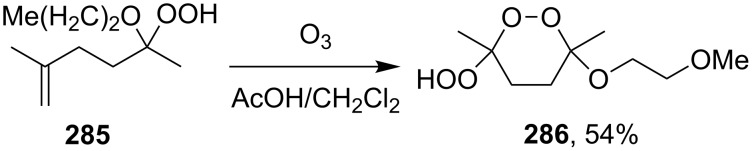
Preparation of 1,2-dioxane **286**.

Under similar conditions, the reaction of 5-hydroperoxy-5-(2-methoxyethoxy)-2-methylhex-1-ene (**285**) in AcOH/CH_2_Cl_2_ produced 3-hydroperoxy-6-(2-methoxyethoxy)-3,6-dimethyl-1,2-dioxane (**286**) (Scheme 80) [270].

#### 3.11. Other methods for the synthesis of 1,2-dioxanes

**Scheme 81:**
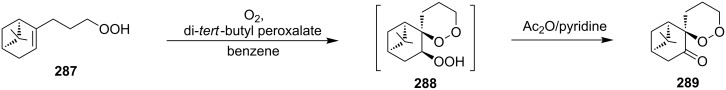
Di(*tert*-butyl)peroxalate-initiated radical cyclization of unsaturated hydroperoxide **287**.

The di(*tert*-butyl)peroxalate-initiated radical cyclization of unsaturated 2-(3-hydroperoxypropyl)-6,6-dimethylbicyclo[3.1.1]hept-2-ene hydroperoxide (**287**) in the presence of oxygen gave 1,2-dioxane (**289**). The reaction proceeds through formation of compound **288** containing a hydroperoxide group, which is transformed into a carbonyl group by treatment with Ac_2_O/pyridine (Scheme 81) [232]. The yield of **289** was 14% based on **287**.

**Scheme 82:**
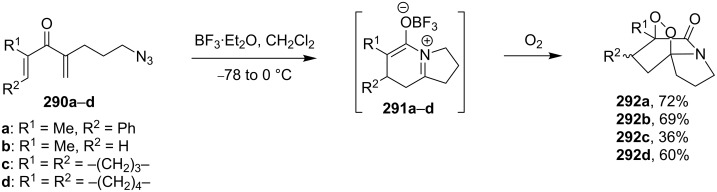
Oxidation of 1,4-betaines **291a–d**.

The original cyclization occurs during the oxidation of 1,4-betaines **291a–d** prepared from dienones **290a–d** containing an azide group in the side chain. The reaction yields peroxide-bridged indolizinediones **292a–d** (Scheme 82) [339].

#### 3.12. Structural modifications, in which 1,2-dioxane ring remains intact

This section deals with syntheses of compounds exhibiting high antimalarial activity that is comparable with or higher than that of artemisinin.

**Scheme 83:**
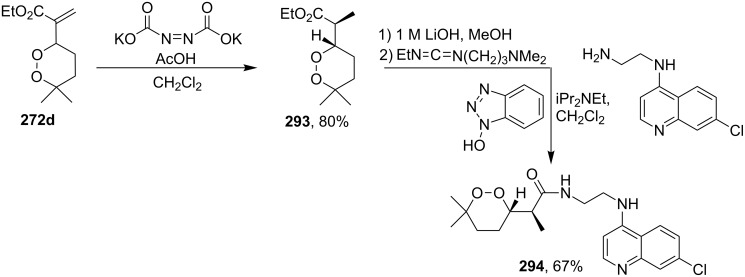
Synthesis of aminoquinoline-containing 1,2-dioxane **294**.

*N*-(2-(7-Chloroquinolin-4-ylamino)ethyl)-2-((*S*)-6,6-dimethyl-1,2-dioxan-3-yl)propanamide (**294**) containing the aminoquinoline moiety that is characteristic for antiparasitic compounds was synthesized by the following series of steps: reduction of the double bond in the presence of the peroxide group (transformation of ethyl 2-(6,6-dimethyl-1,2-dioxan-3-yl)acrylate (**272d**) into ethyl 2-((*S*)-6,6-dimethyl-1,2-dioxan-3-yl)propanoate (**293**)), alkaline hydrolysis, and amidation (Scheme 83) [336].

**Scheme 84:**
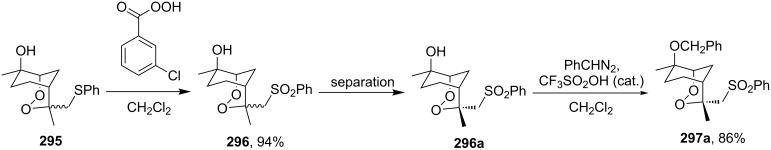
Synthesis of the sulfonyl-containing 1,2-dioxane.

The synthesis of the sulfonyl-containing 1,2-dioxane 2-(benzyloxy)-2,6-dimethyl-6-(phenylsulfonylmethyl)-7,8-dioxabicyclo[3.3.1]nonane )**297a**), included the following steps: oxidation of the sulfide group in 2,6-dimethyl-6-(phenylthiomethyl)-7,8-dioxabicyclo[3.3.1]nonan-2-ol (**295**) to form 2,6-dimethyl-6-(phenylsulfonylmethyl)-7,8-dioxabicyclo[3.3.1]nonan-2-ol (**296**) followed by the isolation of the isomer (6*R*)-2,6-dimethyl-6-(phenylsulfonylmethyl)-7,8-dioxabicyclo[3.3.1]nonan-2-ol (**296a**) and benzylation of the latter to obtain the target peroxide **297a** (Scheme 84) [107].

**Scheme 85:**
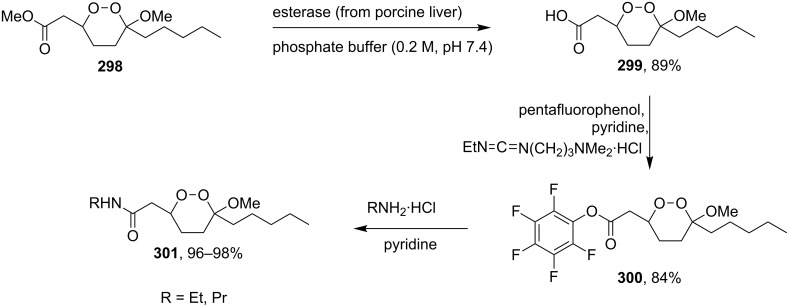
Synthesis of the amido-containing 1,2-dioxane **301**.

Methyl 2-(6-methoxy-6-pentyl-1,2-dioxan-3-yl)acetate (**298**) was enzymatically hydrolyzed to 2-(6-methoxy-6-pentyl-1,2-dioxan-3-yl)acetic acid (**299**). The next step in the synthesis of target compound **301** involved the two-step amidation via the intermediate formation of perfluorophenyl 2-(6-methoxy-6-pentyl-1,2-dioxan-3-yl)acetate (**300**) (Scheme 85) [110].

The enzymatic hydrolysis step was necessary because attempts to hydrolize ester **298** under alkaline conditions (LiOH in dimethyl sulfoxide) failed and led to peroxide ring-opening [110].

### 4. Synthesis of 1,2-dioxenes

#### 4.1. Reaction of 1,3-dienes with singlet oxygen

**Scheme 86:**
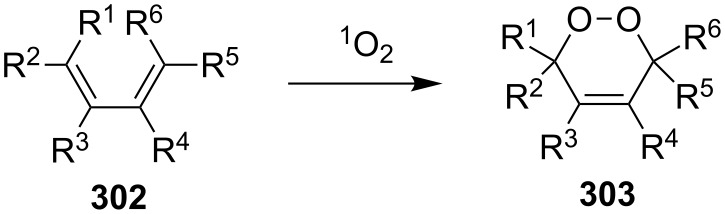
Reaction of singlet oxygen with the 1,3-diene system **302**.

The reaction of singlet oxygen with the 1,3-diene system can proceed by the following pathways: the [4 + 2]-cycloaddition, the singlet-oxygen–ene reaction, and the [2 + 2]-cycloaddition to form dioxetanes. The reaction pathway depends on the nature of the solvent, and on electronic and steric factors. However, the [4 + 2]-cycloaddition (**302** + ^1^O_2_) occurs in most cases, and this reaction is frequently used for the synthesis of the 1,2-dioxene system **303** (Scheme 86). Table 18 gives examples of 1,2-dioxenes synthesized by the reaction of singlet oxygen with 1,3-diene systems.

**Table 18:** Examples of the use of ^1^O_2_ in the synthesis of 1,2-dioxenes.

Alkene **302**	Reaction conditions	1,2-Dioxene **303**	Yield, %	Reference

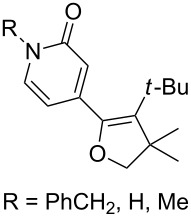	O_2_, *h*ν, tetraphenylporphyrin, CH_2_Cl_2_, −78 °C, 1 h	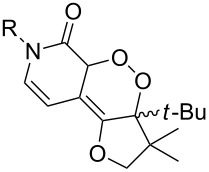	100	[340]
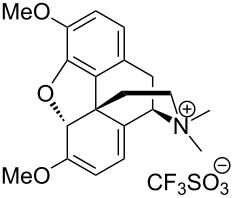	O_2_, *h*ν, tetraphenylporphyrin, CH_2_Cl_2_, −78 °C, 2 h	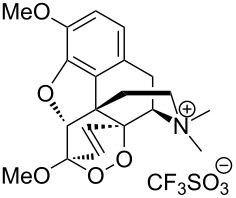	85	[341]
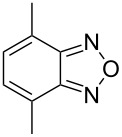	O_2_, hν, fullerene C_60_, CDCl_3_, 0 °C, 2 h	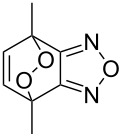	93	[342]
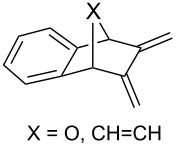	O_2_, *h*ν, tetraphenylporphyrin, CCl_4_	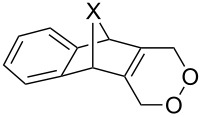	75	[343]
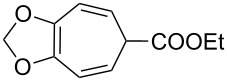	O_2_, *h*ν, tetraphenylporphyrin, CCl_4_, rt, 30 min	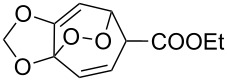	90	[344]
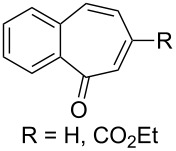	O_2_, *h*ν, tetraphenylporphyrin, CCl_4_, rt, 18–20 h	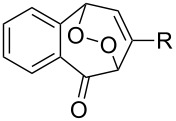	74 95	[345]
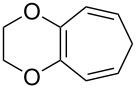	O_2_, *h*ν, tetraphenylporphyrin, CCl_4_, 10 °C, 1.5 h	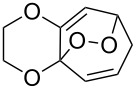	73	[346]
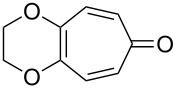	O_2_, *h*ν, tetraphenylporphyrin, CHCl_3_, 10 °C, 45 min	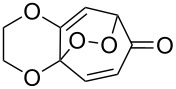	94	[346]
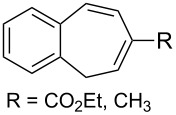	O_2_, *h*ν, tetraphenylporphyrin, CCl_4_, rt, 24 h to 9 d	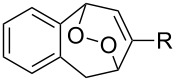	94 36	[347]
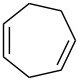	O_2_, *h*ν, tetraphenylporphyrin, CH_2_Cl_2_, −10 °C, 6 h	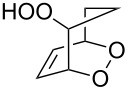	54	[348]
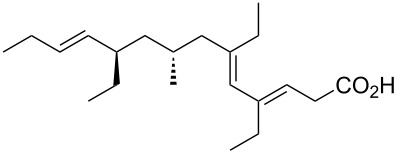	1) O_2_, *h*ν, Rose Bengal, MeOH/CH_2_Cl_2_ (1/19), 0 °C, 8 h 2) CH_2_N_2_	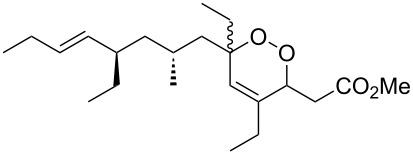	40	[182–183]
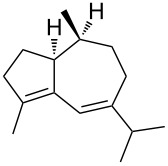	1) O_2_, *h*ν, methylene blue, CH_2_Cl_2_, 15 °C, 30 min 2) PPh_3_, acetone, rt, 40 min	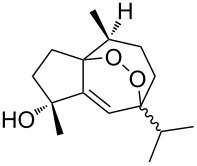	50	[349]
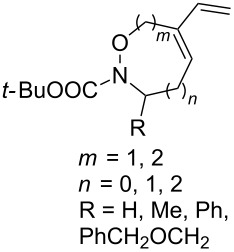	O_2_, *h*ν, Rose Bengal, MeCN, 0 °C, 6–16 h	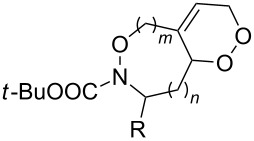	54–82	[350]
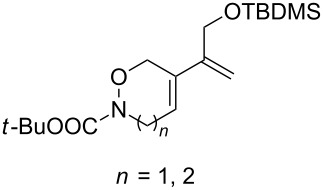	O_2_, *h*ν, Rose Bengal, MeOH/CH_2_Cl_2_ (1/19), 0 °C, 6 h	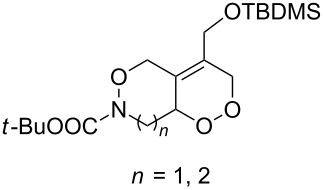	42 66	[351]
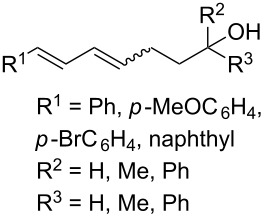	O_2_, *h*ν, Rose Bengal, CH_2_Cl_2_, 6 h	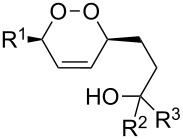	23–70	[352]
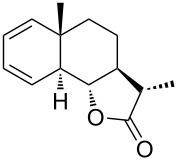	O_2_, *h*ν, tetraphenylporphyrin, benzene, 18 h	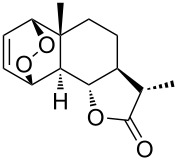	28	[353]
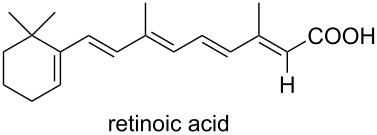	O_2_, *h*ν, Rose Bengal, EtOH, 10 °C, 70 min	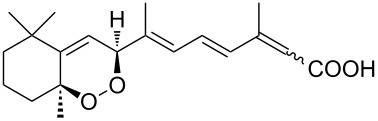	56	[186–190]
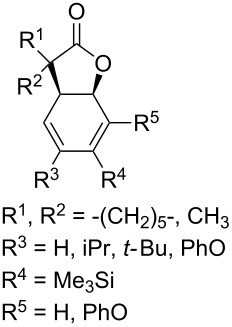	O_2_, *h*ν, tetraphenylporphyrin, CH_2_Cl_2_	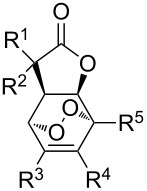	13–95	[191–195]
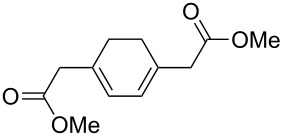	O_2_, *h*ν, Rose Bengal, CH_2_Cl_2_	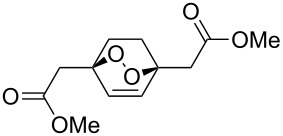	89	[354]
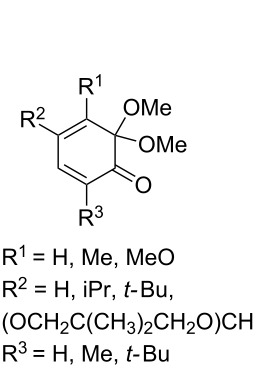	O_2_, *h*ν, tetraphenylporphyrin, CHCl_3_ or Rose Bengal, MeOH, −15 to 0 °C, 1–4 h	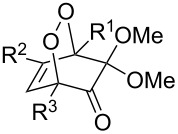	70–92	[355]
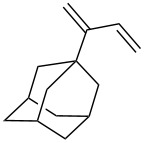	O_2_, *h*ν, Rose Bengal, CH_2_Cl_2_, 6 h	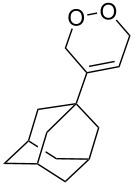	91	[356]
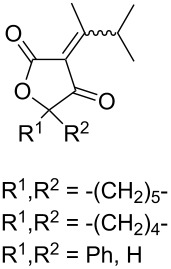	air, sunlight, CHCl_3_, rt, 5 d or O_2_, *h*ν, CH_2_Cl_2_, CuSO_4_, TsOH, 1 h	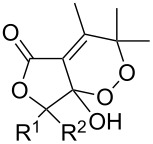	53–80	[357–358]
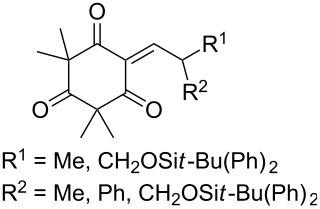	air, sunlight, CHCl_3_, rt, 3 d	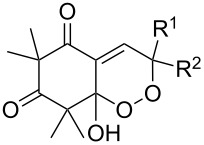	60–85	[113–116] [359–360]
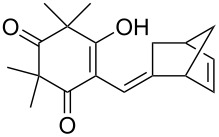	air, sunlight, CHCl_3_, rt, 3 d	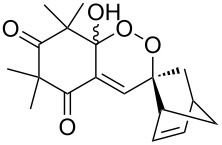	23	[119]
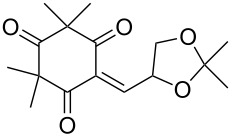	air, sunlight, CHCl_3_, rt, 6 d	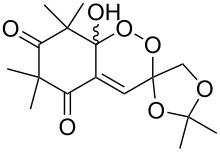	15	[119]
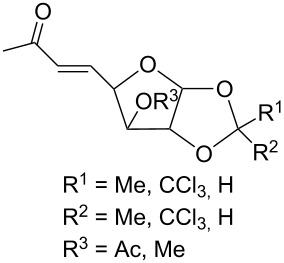	air, sunlight, CCl_4_, rt, 160 h	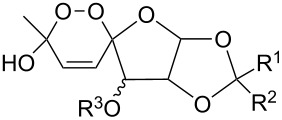	55–80	[361]

(+)-Premnalane A is a natural compound of plant origin exhibiting pronounced antimicrobial activity. The synthesis of this compound includes the following steps: oxidation of the furan ring of compound **304**, the singlet-oxygen–ene reaction of the double bond-containing bicyclic compound **305**, and acid-induced ketalization (Scheme 87) [362].

**Scheme 87:**
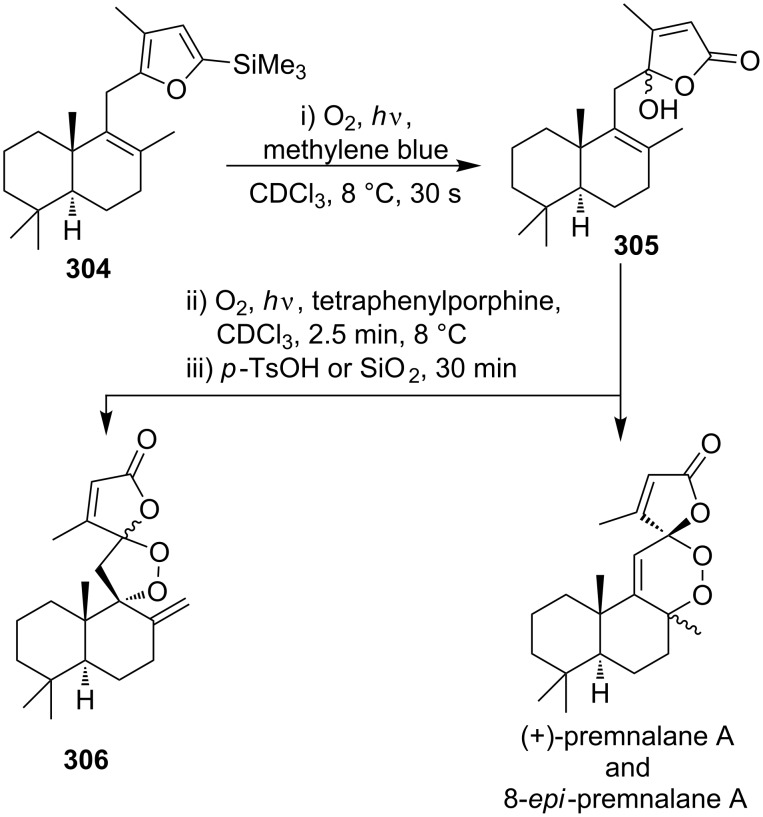
Synthesis of (+)-premnalane А and 8-epi-premnalane A.

This synthesis produced a 1:1 mixture of diastereomeric (+)-premnalane А and 8-epi-premnalane A in 24% combined yield and diastereomeric 1,2-dioxolanes **306** in 49% yield. Pure (+)-premnalane A was isolated by column chromatography.

#### 4.2. Structural modifications, in which 1,2-dioxene ring remains intact

**Scheme 88:**
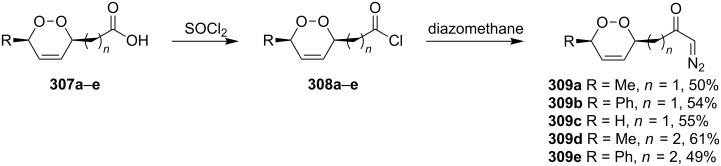
Synthesis of the diazo group containing 1,2-dioxenes **309a–e**.

Diazo-containing 1,2-dioxenes **309a–e** were synthesized starting from the corresponding acids **307a–e**, which were transformed into acid chlorides **308a–e** and then subjected to diazotization (Scheme 88) [363]. The 1,2-dioxenes **309a–e** were used for the intramolecular insertion of carbenes, that were produced by decomposition of the diazo group, into the –О–О–bond [363].

**Figure 4:**
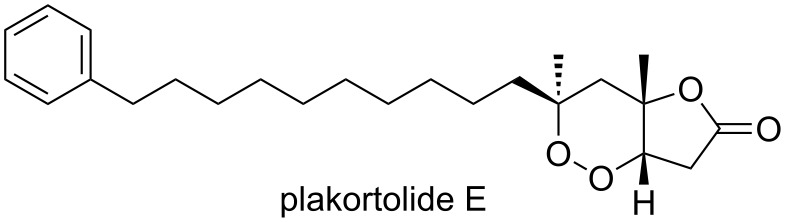
Plakortolide Е.

6-Epiplakortolide Е is a bicyclic peroxylactone that was isolated in low yield (0.0003%) from the marine sponge *Plakortis* sp. The structurally related plakortolide Е (Figure 4) exhibits high cytotoxicity against cancer cells and shows also activity against *Toxoplasma gondii*, which is the causative agent of toxoplasmosis [184–185].

**Scheme 89:**
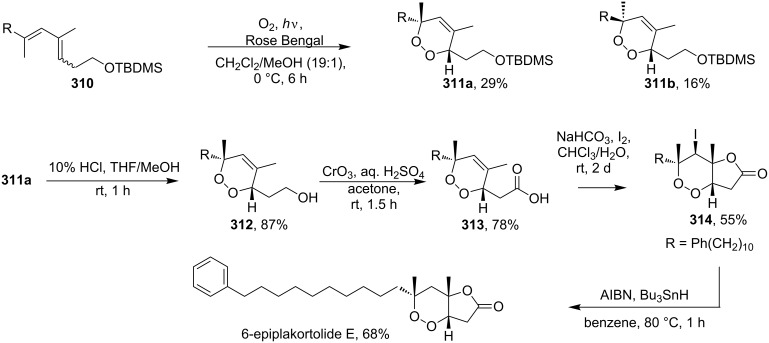
Synthesis of 6-epiplakortolide Е.

6-Epiplakortolide Е was synthesized by the multistep synthesis involving the oxidation of diene **310** with singlet oxygen to give two isomeric 1,2-dioxenes **311a**,**b**, the isolation of dioxene **311a**, its silyl deprotection to form alcohol **312**, the oxidation of the latter to 1,2-dioxenic acid **313**, the I**^+^**-induced lactonization to produce **314**, and the deiodination to obtain the target product (Scheme 89) [184–185]. It should be noted that the cyclic peroxide compound **314** remains intact under the reductive conditions in the presence of tributylstannane; this step occurs in good yield (68%) [184–185].

**Scheme 90:**
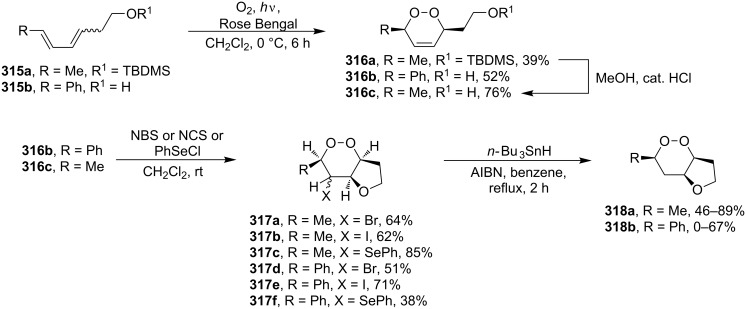
Application of Bu_3_SnH for the preparation of tetrahydrofuran-containing bicyclic peroxides **318a**,**b**.

More recently, a similar approach was used for the preparation of tetrahydrofuran-containing bicyclic peroxides **318a**,**b**. It involves the synthesis of 1,2-dioxenes **316** from dienes **315**, the cation-initiated cyclization to give bicyclic compounds **317**, and the reduction with Bu_3_SnH. *N*-Bromo- and iodosuccinimides (NBS and NIS, respectively) were used as donors of halogenide ions. Additionallay, the cyclization was successfully performed with the use of phenylselenyl chloride as the donor of PhSe^+^ cation (Scheme 90) [364].

**Scheme 91:**
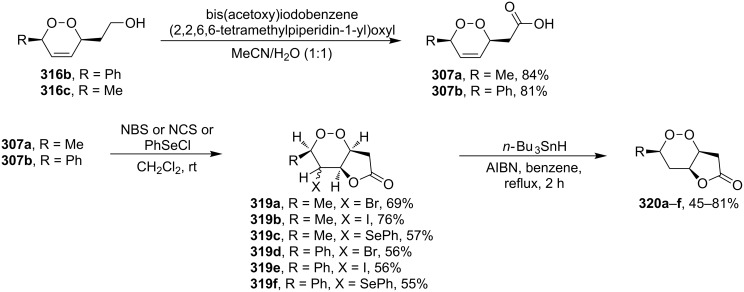
Application of Bu_3_SnH for the preparation of lactone-containing bicyclic peroxides **320a–f**.

Acids **307a** and **307b** were synthesized by oxidation of the corresponding alcohols with the bis(acetoxy)iodobenzene/2,2,6,6-tetramethyl-1-piperidinyl oxyl (BAIB/TEMPO) system. The cyclization to bicyclic peroxides **319a–f** containing the lactone ring was performed with the use of N-bromo- and iodosuccinimides and PhSeCl (Scheme 91) [364]. As in the above-considered case, the peroxide ring remains unchanged upon the reduction of the С–X bond in compounds **319a–f** with Bu_3_SnH [364].

**Scheme 92:**
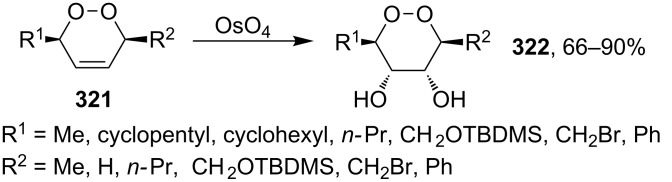
Dihydroxylation of the double bond in the 1,2-dioxene ring **321** with OsO_4_.

The double bond in the 1,2-dioxene ring of **321** was subjected to dihydroxylation with osmium tetroxide (Scheme 92) [354,365]. The reaction was performed in aqueous *tert*-butanol, acetone, or acetonitrile at room temperature. Several methods were used for the oxidation. For example, the commercially available AD-mix, a mixture consisting of K_2_OsO_2_(OH)_4_ (catalytic amounts, a source of OsO_4_) and K_3_Fe(CN)_6_ (oxidizer), was employed for this purpose. In this reaction, K_2_OsO_4_ (0.5 mol %) combined with oxidizers (K_3_Fe(CN)_6_, *N*-methylmorpholine *N*-oxide, citric acid, or KClO_3_) was also used [354,365].

**Scheme 93:**
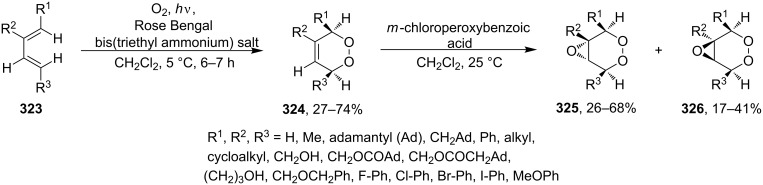
Epoxidation of 1,2-dioxenes **324**.

The epoxidation of 1,2-dioxenes **324** produced by the addition of singlet oxygen to dienes **323** was performed by treatment with *m*-chlorobenzoic acid (Scheme 93). It was shown that epoxidized dioxanes **325** and **326**, as well as dioxenes **324**, have inhibiting activity against the causative agents of candidiasis infections *Candida albicans*, *Candida krusei*, and *Candida tropicalis*, that are in some cases comparable with the activity of the currently used amphotericin B, ketonazole, and nystatin [218–228]. In addition, these compounds exhibit pronounced antimalarial activity, although lower than that of artemisinin [366–367].

**Scheme 94:**
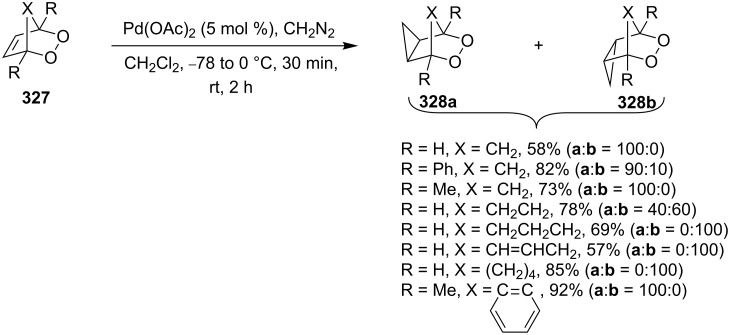
Cyclopropanation of the double bond in endoperoxides **327**.

The cyclopropanation of the double bond in endoperoxides **327** was performed by the reaction with diazomethane in the presence of Pd(OAc)_2_ to produce **328a**,**b** (Scheme 94) [368].

**Scheme 95:**
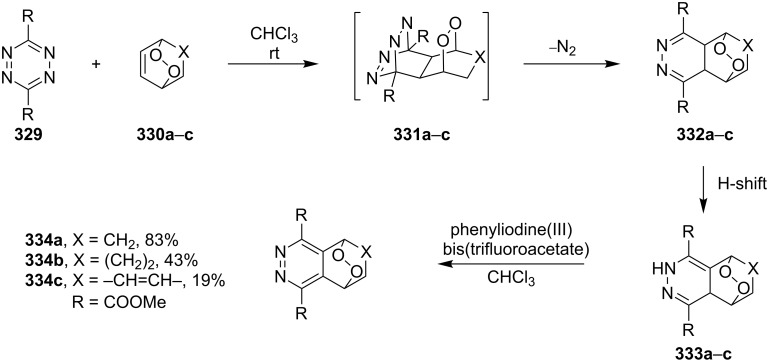
Preparation of pyridazine-containing bicyclic endoperoxides **334a–c**.

Pyridazine-containing bicyclic endoperoxides **334a–c** were synthesized by the inverse-electron-demand Diels–Alder cycloaddition of dimethyl 1,2,4,5-tetrazine-3,6-dicarboxylate (**329**) to 1,2-dioxenes **330** followed by the elimination of dinitrogen from **331a–c** to give **332a–c**, the isomerization to **333a–c**, and the oxidation (Scheme 95) [369].

#### 5. Synthesis of 1,2,4-trioxanes

This part is devoted to methods for the synthesis of the 1,2,4-trioxane ring by the singlet-oxygen ene reaction with unsaturated alcohols, the photooxidation of enol ethers and vinyl sulfides, the [4+2]-cycloaddition of singlet oxygen to the pyran system, the Isayama-Mukaiyama peroxysilylation of unsaturated alcohols, reactions with hydrogen peroxide, and the intramolecular Kobayashi cyclization.

#### 5.1. Use of singlet oxygen in the synthesis of 1,2,4-trioxane

**Scheme 96:**
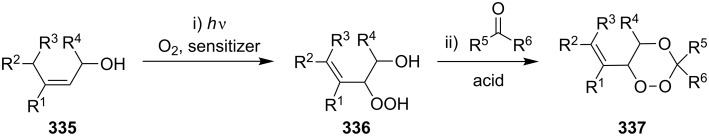
Synthesis of 1,2,4-trioxanes **337** by the hydroperoxidation of unsaturated alcohols **335** with ^1^O_2_ and the condensation of the hydroxy hydroperoxides **336** with carbonyl compounds.

One of the widely used approaches to the synthesis of the 1,2,4-trioxane ring **337** is based on the hydroperoxidation of unsaturated alcohols **335** with singlet oxygen (the singlet-oxygen ene reaction) and the acid-catalyzed condensation of the resulting vicinal hydroxy hydroperoxides **336** with ketones or aldehydes (acetals, orthoesters) (Scheme 96, Table 19).

The method was described for the first time by Singh in 1990 [370]. Due to a wide structural series of prepared 1,2,4-trioxane systems and the use of readily available inexpensive reagents, this is an efficient method for their synthesis.

**Table 19:** Examples of 1,2,4-trioxanes synthesized by the singlet oxygen ene reaction.

Alkene **335**	Carbonyl compound	Reaction conditions^a^	Product **337**	Yield i) **336**, % ii) **337**, %	Reference

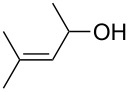	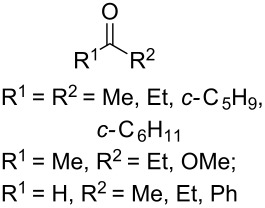	1) O_2_, *h*ν, TPP 2) BF_3_·Et_2_O, CH_2_Cl_2_	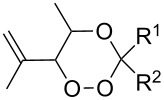	i) 88–94 ii) 63–78	[371]
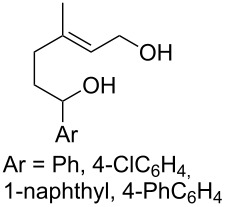	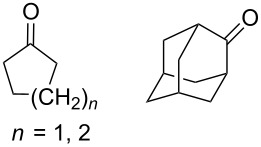	1) O_2_, *h*ν, methylene blue, MeCN, 0 °C, 4–6 h 2) TsOH, CH_2_Cl_2_, rt, 1 h	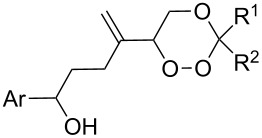	i) 30–45 ii) 50–74	[372]
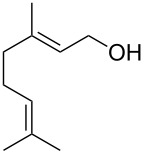	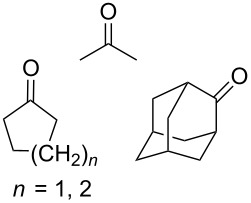	1) O_2_, *h*ν, methylene blue, MeCN, −10 °C 2) acid 3) NaBH_4_, MeOH	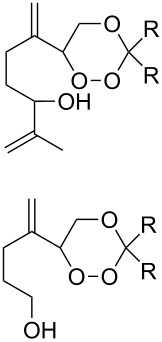	The yield was not determined	[373]
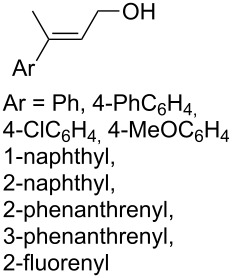	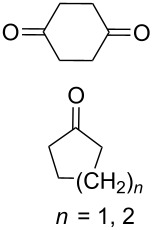	1) O_2_, *h*ν, methylene blue, MeCN, 0 °C, 4–6 h 2) HCl, 5°C, 18 h	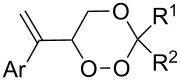	22–51	[374–379]
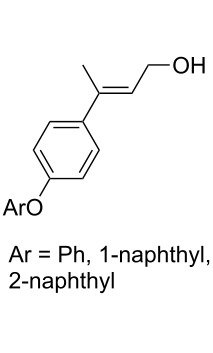	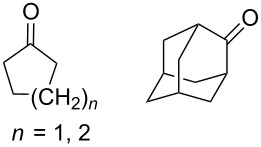	1) O_2_, *h*ν, methylene blue, MeCN, 0 °C, 4–6 h 2) HCl, rt, 1 h	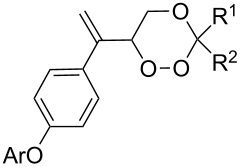	45–70	[380]
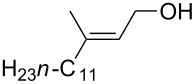	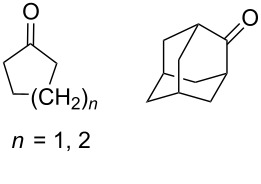	1) O_2_, *h*ν, methylene blue, MeCN, 0 °C, 6 h 2) TsOH, CH_2_Cl_2_, rt, 1 h	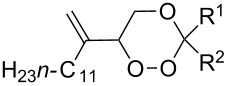	i) 43 ii) 65–76	[381]
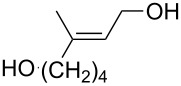	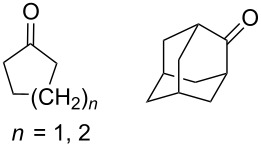	1) O_2_, *h*ν, methylene blue, MeCN, 0 °C, 6 h 2) TsOH, CH_2_Cl_2_, rt, 1 h	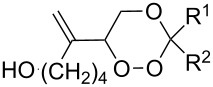	i) 37 ii) 46–59	[381]
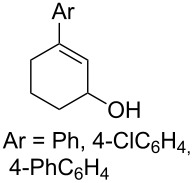	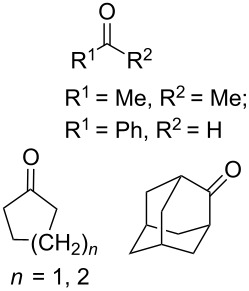	1) O_2_, *h*ν, methylene blue, MeCN, 0 °C, 18 h 2) HCl, CH_2_Cl_2_, 0 °C, 3–6 h	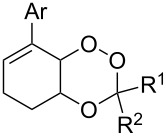	i) 22–35 ii) 12–37	[382]
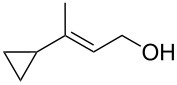	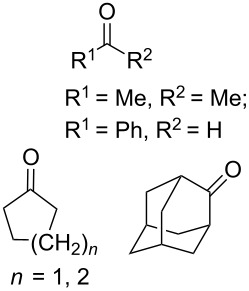	1) O_2_, *h*ν, methylene blue, MeCN, 0 °C, 6 h 2) TsOH, CH_2_Cl_2_, rt, 2 h	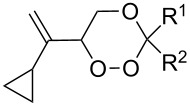	i) 50 ii) 31–75	[383]
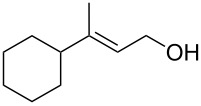	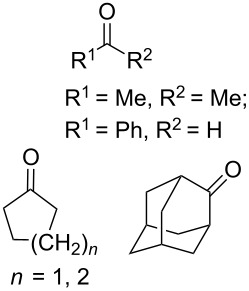	1) O_2_, *h*ν, methylene blue, MeCN, 0 °C, 6 h; 2) TsOH, CH_2_Cl_2_, rt, 2 h	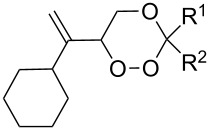	i) 35 ii) 57–83	[383]
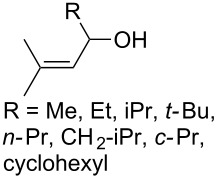	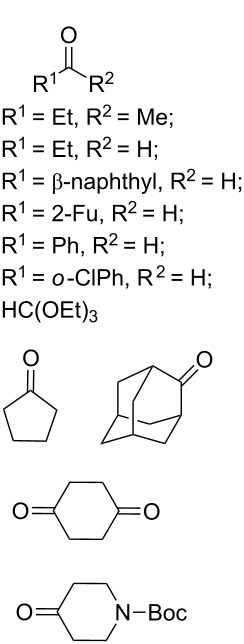	1) O_2_, *h*ν, TPP 2) BF_3_·Et_2_O, Et_2_O, 0 °C	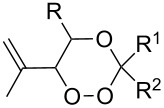	i) 54–97 ii) 8–78	[384–387]
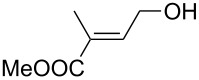	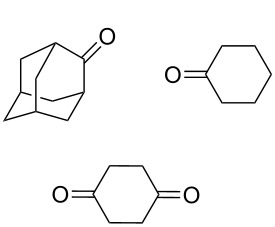	1) O_2_, *h*ν, TTP, polystyrene 2) BF_3_·Et_2_O, CH_2_Cl_2_	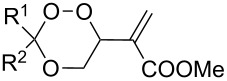	20–86	[385,387–388]
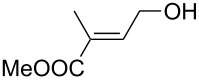	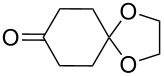	1) O_2_, *h*ν, TTP or TPP PS-DVB-based 2) BF_3_·Et_2_O, CH_2_Cl_2_	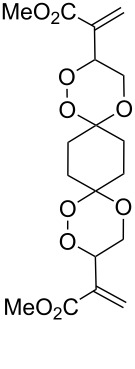	20	[389]
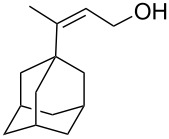	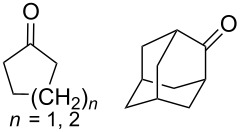	1) O_2_, *h*ν, methylene blue, MeCN, 0 °C, 4–5 h 2) HCl, CH_2_Cl_2_, rt, 1 h	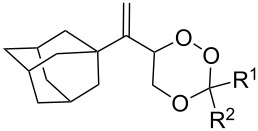	38–54	[390]
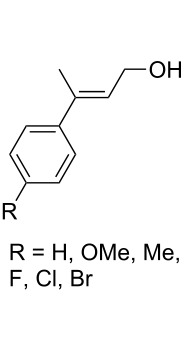	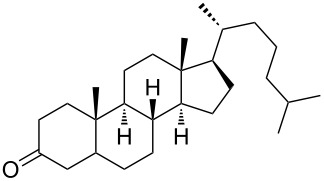	1) O_2_, *h*ν, methylene blue, MeCN, 0 °C, 5 h 2) HCl, MeCN, rt, 3 h	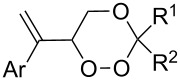	23-69	[125–127]
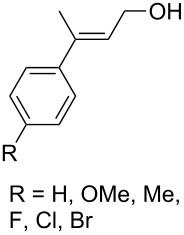	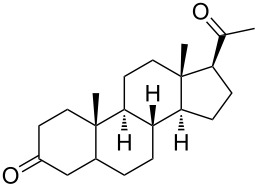	1) O_2_, *h*ν, methylene blue, MeCN, 0 °C, 5 h 2) HCl, MeCN, rt, 3 h	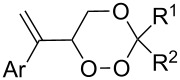	40–87	[125–127]
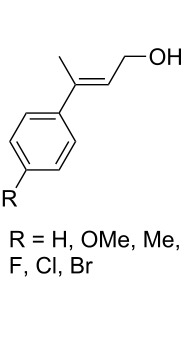	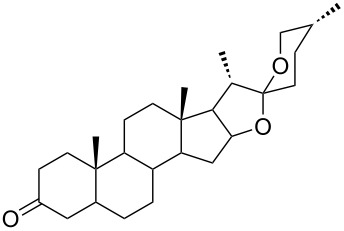	1) O_2_, *h*ν, methylene blue, MeCN, 0 °C, 5 h 2) HCl, MeCN, rt, 3 h	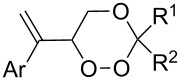	35–55	[125–127]
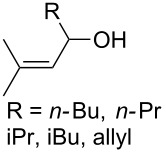	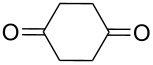	1) O_2_, *h*ν, TTP or TPP PS-DVB-based 2) BF_3_·Et_2_O, CH_2_Cl_2_	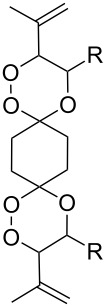	4–19	[389]
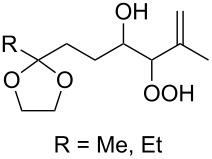	Intramolecular process	1) O_2_, *h*ν, TTP or TPP PS-DVB-based 2) BF_3_·Et_2_O, CH_2_Cl_2_	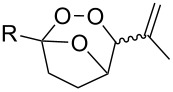	i) 84–91 ii) 12–19	[391]
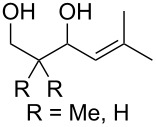	R^1^C(OMe)_3_ R^1^ = Me, Et, *n*-Pr, Ph	1) O_2_, *h*ν, TPP, CCl_4_, 10 °C 2) PPTS, CH_2_Cl_2_	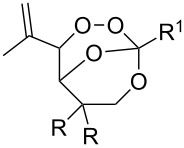	i) 94–99 ii) 21–30	[391]
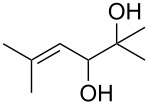	MeC(OMe)_3_	1) O_2_, *h*ν, TPP, CCl_4_, 10 °C 2) PPTS, CH_2_Cl_2_	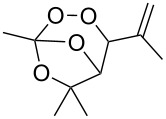	i) 83 ii) 19	[391]
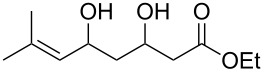	RC(OMe)_3_ R = Me, Et, *n*-Pr	1) O_2_, *h*ν, TPP, CCl_4_, rt 2) TsOH, CH_2_Cl_2_, rt	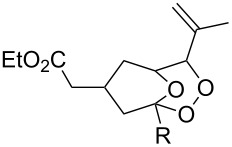	i) 83 ii) 18–55	[392]

^a^TPP is tetraphenylporphyrin; TTP is tetratolylporphyrin; PPTS is pyridinium *para*-toluenesulfonate.

**Scheme 97:**
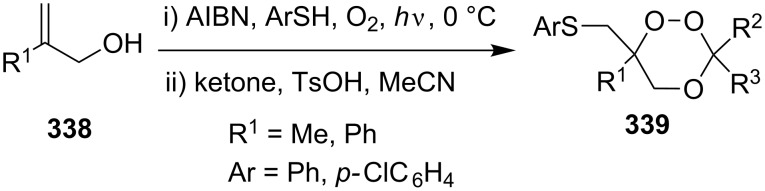
Synthesis of sulfur-containing 1,2,4-trioxanes **339**.

A similar approach based on the co-oxidation of hydroxyalkenes **338** and thiols (TOCO-reaction, thiol–olefin co-oxygenation) was applied to the synthesis of sulfur-containing 1,2,4-trioxanes **339** (Scheme 97).

Azobisisobutyronitrile (AIBN) was used as the initiator of the radical reaction. In the second step (condensation), cyclopentanone, cyclohexanone, *tert*-butylcyclohexanone, 1,4-cyclohexanedione, cyclododecanone, and adamantanone were employed. 1,2,4-Trioxanes **339** were prepared in 25–68% yields in two steps [120,393].

**Scheme 98:**
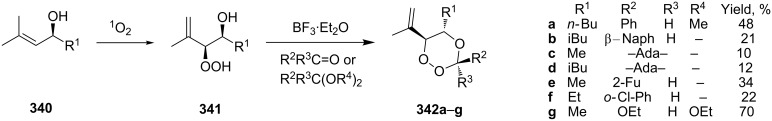
BF_3_·Et_2_O-catalyzed synthesis of the 1,2,4-trioxanes **342a**–**g**.

The formation of peroxyketals **342a–g** from vicinal hydroxyhydroperoxides **341** (oxidation products of unsaturated alcohols **340**) in the presence of boron trifluoride is a convenient approach to the synthesis of the 1,2,4-trioxane ring (Scheme 98) [385].

**Scheme 99:**
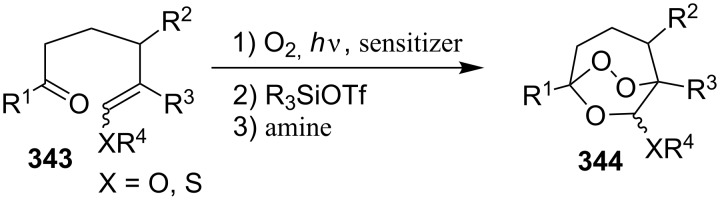
Photooxidation of enol ethers or vinyl sulfides **343**.

The approach to the synthesis of 1,2,4-trioxanes proposed by Jefford and co-workers in 1993 [394] is based on the photooxidation of enol ethers or vinyl sulfides **343** with oxygen followed by the rearrangement of the resulting 1,2-dioxetanes in the presence of trialkylsilyl triflates. The resulting bicyclic compound **344** is structurally similar to artemisinin. Another version of this synthesis is based on the use of the ozone/triphenylphosphite in the oxidation step 1) (Scheme 99, Table 20).

**Table 20:** Examples of 1,2,4-trioxanes synthesized by oxidation of enol ethers or vinyl sulfides.

Enol ether or vinyl sulfide **343**	Reaction conditions	Product **344**	Yield, %	Reference

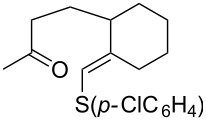	1) O_2_, *h*ν, methylene blue, CH_2_Cl_2_, −78 °C 2) TBDMSOTf, CH_2_Cl_2,_ −78 °C 3) Et_3_N, CH_2_Cl_2_, −78 °C to −15 °C	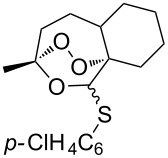	47 (12-α) 47 (12-β)	[395]
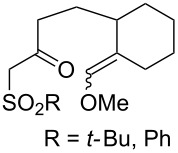	1) O_3_, (PhO)_3_P, CH_2_Cl_2_, −78 °C 2) Et_3_SiOTf, CH_2_Cl_2_, −78 °C 3) Et_3_N, CH_2_Cl_2_, −78 °C to rt	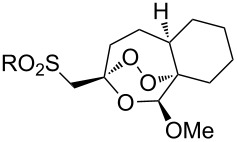	30–38	[395]
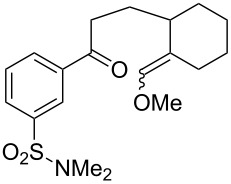	1) O_3_, (PhO)_3_P, CH_2_Cl_2_, −78 °C 2) Me_3_SiOTf, CH_2_Cl_2_, −78 °C 3) Et_3_N, CH_2_Cl_2_, −78 °C to rt	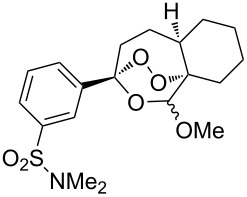	7	[395]
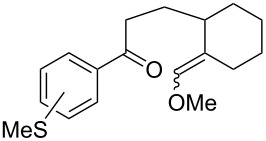	1) O_3_, (PhO)_3_P, CH_2_Cl_2_, −78 °C 2) Me_3_SiOTf, CH_2_Cl_2_, −78 °C 3) Et_3_N, CH_2_Cl_2_, −78 °C to rt	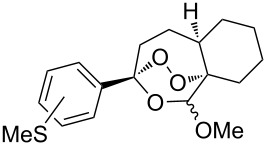	3 (12-α) 11 (12-β)	[395]
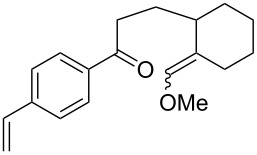	1) Air, *h*ν, methylene blue, CH_2_Cl_2_, −90 °C 2) Me_3_SiOTf, CH_2_Cl_2_, −90 °C, 1 h 3) 1- ethylpiperidine, CH_2_Cl_2_, −78 °C to rt	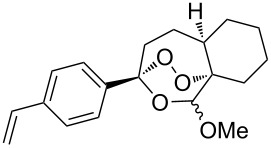	36 (12-α) 15 (12-β)	[396]
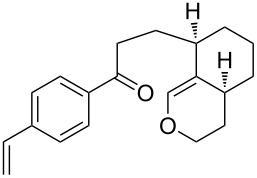	1) Air, *h*ν, methylene blue, CH_2_Cl_2_, −78 °C, 1 h 2) Me_3_SiOTf, CH_2_Cl_2_, −78 °C, 2 h 3) 1-ethylpiperidine, CH_2_Cl_2_, −78 °C to rt	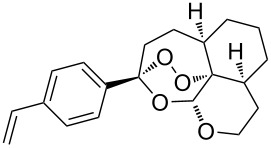	33	[397]

**Scheme 100:**
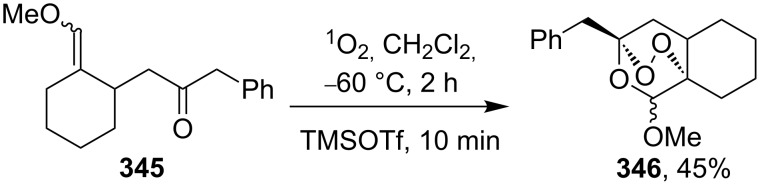
Synthesis of tricyclic peroxide **346**.

This method was applied to the synthesis of tricyclic peroxide **346** (containing one carbon atom less in the mono-oxygen ring compared to structures **344**) from the enol ether, 1-(2-(methoxymethylene)cyclohexyl)-3-phenylpropan-2-one (**345**) (Scheme 100) [207].

**Scheme 101:**
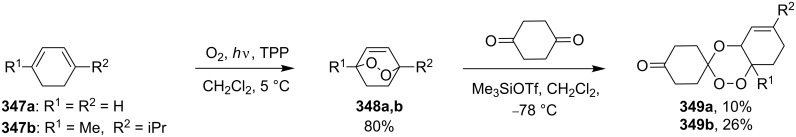
Reaction of endoperoxides **348a**,**b** derived from cyclohexadienes **347a**,**b** with 1,4-cyclohexanedione.

The reaction of endoperoxides **348a**,**b** derived from cyclohexadienes **347a**,**b** with 1,4-cyclohexanedione produced trioxanes **349a**,**b** containing a keto group which is useful for further transformations (Scheme 101) [398].

**Scheme 102:**
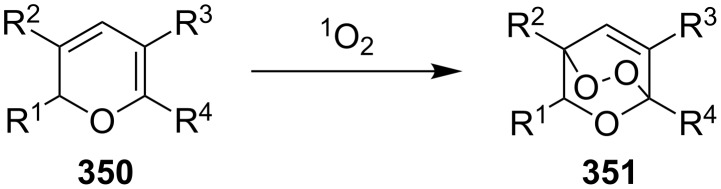
[4 + 2]-Cycloaddition of singlet oxygen to 2*Н*-pyrans **350**.

Unsaturated bicyclic trioxanes **351** are [4 + 2]-cycloaddition products of singlet oxygen to the pyran moiety in **350** (Scheme 102, Table 21).

**Table 21:** Examples of 1,2,4-trioxanes synthesized by oxidation of 2*Н*-pyrans.

Pyran **350**	Reaction conditions	1,2,4-Trioxane **351**	Yield, %	Reference

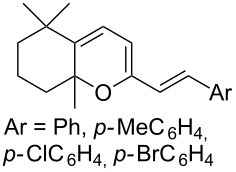	O_2_, *h*ν, benzene	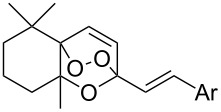	85–90	[399]
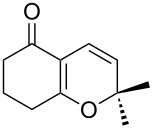	Air, Rose Bengal, *h*ν, THF, −78 °C	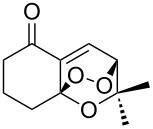	94	[400]
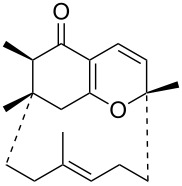	Air, Rose Bengal, *h*ν, CH_2_Cl_2_, −78 °C	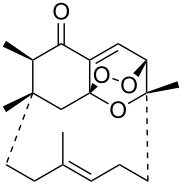	65	[401]

It was shown that in this reaction the starting pyran can serve as the sensitizer for the formation of singlet oxygen [402].

#### 5.2. Synthesis of 1,2,4-trioxanes by the Isayama–Mukaiyama method

**Scheme 103:**
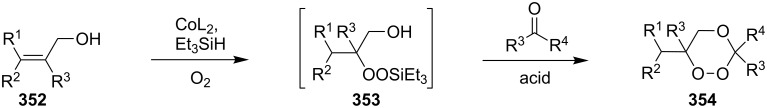
Synthesis of 1,2,4-trioxanes **354** using peroxysilylation stage.

The Isayama–Mukaiyama peroxysilylation of unsaturated alcohols **352** is a new route to hydroxy silyl peroxides **353**, whose condensation with ketones in an acidic medium affords 1,2,4-trioxanes **354** (Scheme 103, Table 22).

**Table 22:** Examples of 1,2,4-trioxanes synthesized through the Isayama–Mukaiyama peroxysilylation.

Unsaturated alcohol **352**	Carbonyl compound	Reaction conditions^a^	1,2,4-Trioxane **354**	Yield, %	Reference

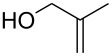	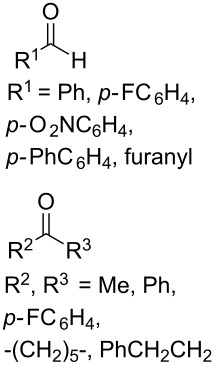	1) Co(acac)_2_, Et_3_SiH, O_2_, rt 2) TsOH	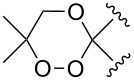	1) 60 2) 40–90	[403]
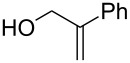	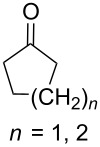	1) Co(acac)_2_, Et_3_SiH, O_2_, rt 2) TsOH	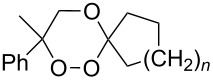	42 54	[403]
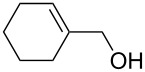	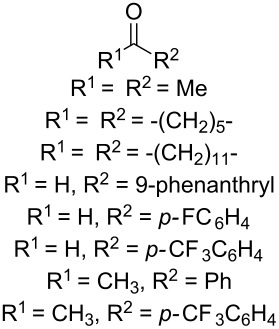	1) Co(thd)_2_, Et_3_SiH, O_2_, rt 2) TsOH	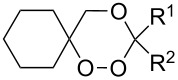	40–85	[404]
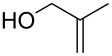	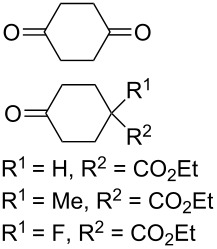	1) Co(acac)_2_, Et_3_SiH, O_2_, EtOH, 4 h 2) TsOH, CHCl_3_, 45 min to 3.5 h	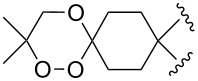	1) 36 2) 57–100	[153–158]

^a^(Co(II)(thd)_2_) is bis(2,2,6,6-tetramethyl-3,5-heptanedionato)cobalt (II).

#### 5.3. Use of epoxides as starting reagents in the synthesis of 1,2,4-trioxanes

**Scheme 104:**
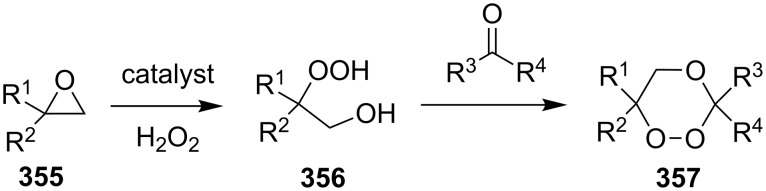
Epoxide-ring opening in **355** with H_2_O_2_ followed by the condensation of hydroxy hydroperoxides **356** with ketones.

An important approach to the synthesis of 1,2,4-trioxanes **357** is based on the epoxide-ring opening in **355** with hydrogen peroxide in the presence of a catalyst followed by the condensation of the vicinal hydroxy hydroperoxides **356** with ketones (Scheme 104, Table 23). The drawbacks of this method are generally low yields of **356** in the step of the epoxide–ring opening and difficulties of their isolation from the reaction mixture.

**Table 23:** Examples of 1,2,4-trioxanes **357** synthesized based on epoxides **355**.

Epoxide **355**	Carbonyl compound	Reaction conditions	1,2,4-Trioxane **357**	Yield i) **356** ii) **357**, %	Ref.

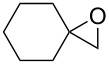	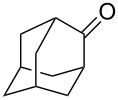	1) MoO_2_(acac)_2_, H_2_O_2_ , Et_2_O, MgSO_4_ 2) TsOH, CH_2_Cl_2_, rt	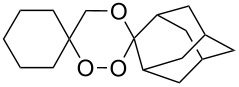	i) 59 ii) 95	[405]
	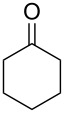		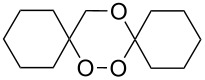	i) 59 ii) 69	[405]
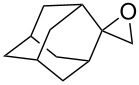	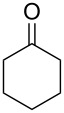	1) MoO_2_(acac)_2_, H_2_O_2_, THF, MgSO_4_ 2) 10-camphor- sulfonic acid, CH_2_Cl_2_, rt	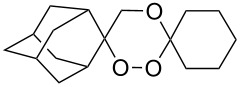	i) 29 ii) 46	[405]
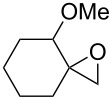	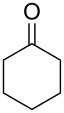	1) H_2_O_2_, Et_2_O, 0 °C, 4 h 2) H_2_SO_4_, CH_2_Cl_2_, rt, 4 d	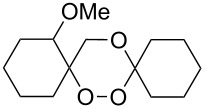	i) 8 ii) 28	[406]
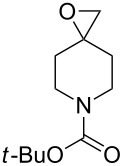	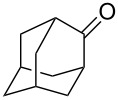	1) MoO_2_(acac)_2_, H_2_O_2_, Et_2_O, MgSO_4_, rt, 22 h 2) TsOH, CH_2_Cl_2_, rt, 5 h	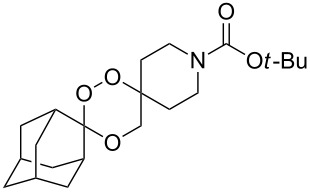	i) 98 ii) 92	[407]
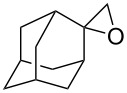	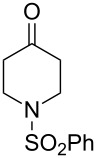	1) MoO_2_(acac)_2_, H_2_O_2_, Et_2_O 2) 10-camphor- sulfonic acid, CH_2_Cl_2_	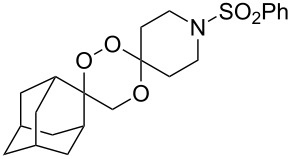	i) 25 ii) 39	[407]
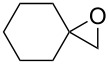	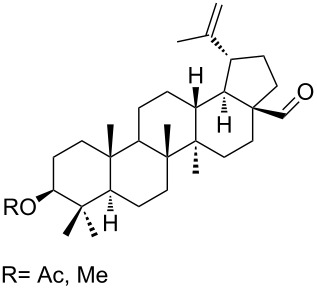	1) MoO_2_(acac)_2_, H_2_O_2_ , Et_2_O, MgSO_4_ 2) BF_3_·Et_2_O, CH_2_Cl_2_, −78 °C to 0 °C, 5 h	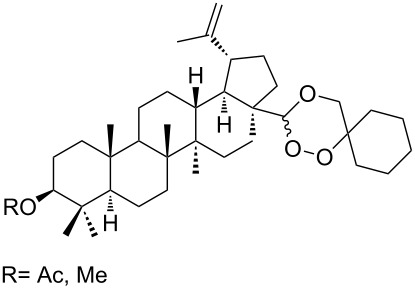	i) - ii) 27–35	[175] [176]

**Scheme 105:**
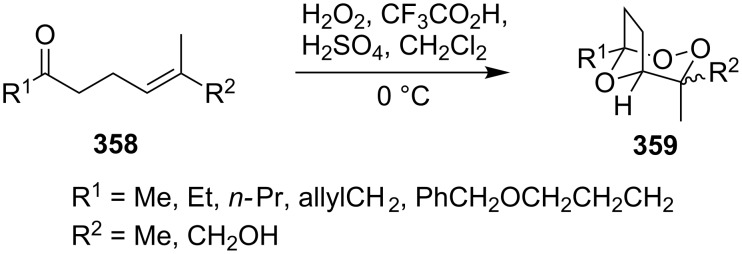
Peroxidation of unsaturated ketones **358** with the H_2_O_2_/CF_3_COOH/H_2_SO_4_ system.

The reaction of unsaturated ketones **358** with H_2_O_2_/CF_3_COOH/H_2_SO_4_ in dichloromethane produced 1,2,4-trioxanes **359** in 25–95% yields (Scheme 105). It is assumed that in the first step, the hydroperoxidation of the keto group in **358** and the epoxidation of the double bond occur followed by the acid-induced intramolecular cyclization to form bicyclic compound **359** [408].

#### 5.4. Synthesis of 1,2,4-trioxanes by the Kobayashi method

**Scheme 106:**
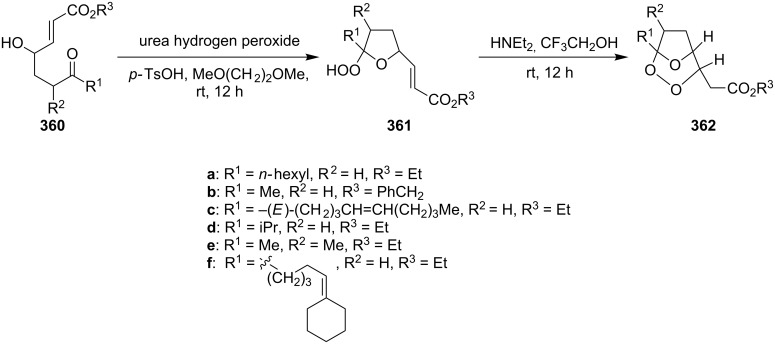
Synthesis of 1,2,4-trioxanes **362** through Et_2_NH-catalyzed intramolecular cyclization.

A convenient method for the synthesis of bicyclic trioxanes **362** was developed based on the hydroperoxidation of polyfunctional compounds **360** with the urea–hydrogen peroxide complex followed by the base-mediated intramolecular cyclization of **361** (Scheme 106). The yield of hydroperoxides **361** was 86–90%. In the second step, the intramolecular cyclization was performed in the presence of a catalytic amount of diethylamine. The yields of trioxanes **362** are in the range of 10–35% [409–410].

#### 5.5. Structural modifications, in which 1,2,4-trioxane ring remains intact

**Scheme 107:**
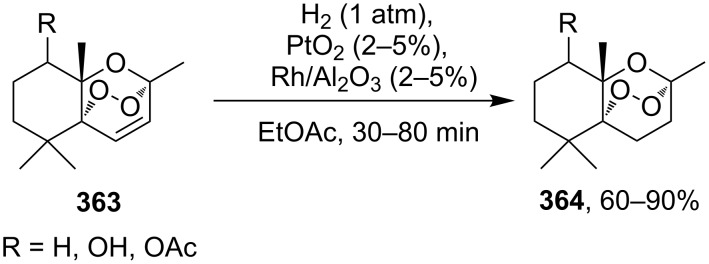
Reduction of the double bond in tricyclic peroxides **363**.

The possibility of the reduction of the double bond in tricyclic peroxides **363** by hydrogen with the use of the mixed platinum–rhodium catalyst to form products, in which the 1,2,4-trioxane moiety remains intact, was exemplified by the synthesis of peroxides **364** (Scheme 107) [411].

**Scheme 108:**
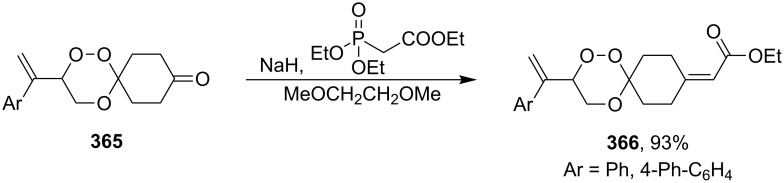
Horner–Wadsworth–Emmons reaction in the presence of peroxide group.

1,2,4-Trioxane esters **366** were synthesized in high yield from 1,2,4-trioxane ketones **365** by the Horner–Wadsworth–Emmons reaction in the presence of sodium hydride as the base (Scheme 108) [375]. Compounds **366** exhibit antimalarial activity comparable with that of artemisinin.

**Scheme 109:**
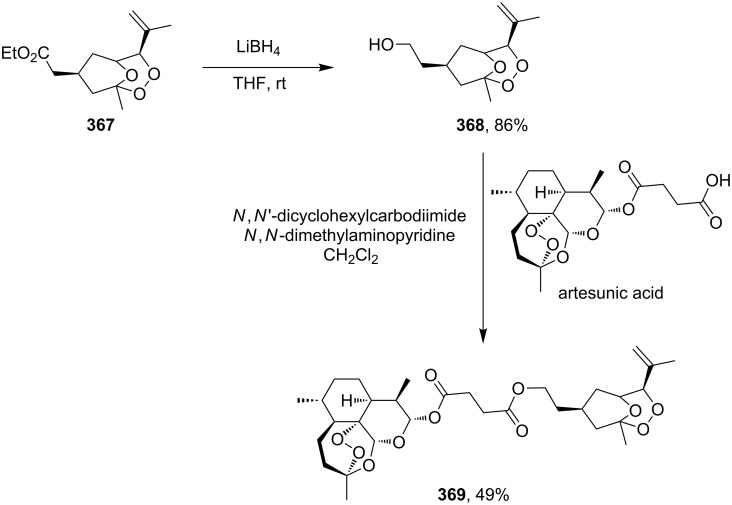
Reduction of ester group by LiBH_4_ in the presence of 1,2,4-trioxane moiety.

Peroxide dyad **369** consisting of 1,2,4-trioxane moieties of different types was synthesized by the esterification of artesunic acid with 2-((3*S*,6*R*)-1-methyl-6-(prop-1-en-2-yl)-7,8,9-trioxabicyclo[3.3.1]nonan-3-yl)ethanol (**368**) (obtained by the reduction of ethyl 2-((3*S*,6*R*)-1-methyl-6-(prop-1-en-2-yl)-7,8,9-trioxabicyclo[3.3.1]nonan-3-yl)acetate (**367**)) in the presence of *N*,*N*’-dicyclohexylcarbodiimide (DCC) (Scheme 109) [392]. The particular structural feature of compound **369** is that it contains a natural peroxide moiety (artesunic acid) combined with the synthetic 1,2,4-trioxane moiety.

**Scheme 110:**
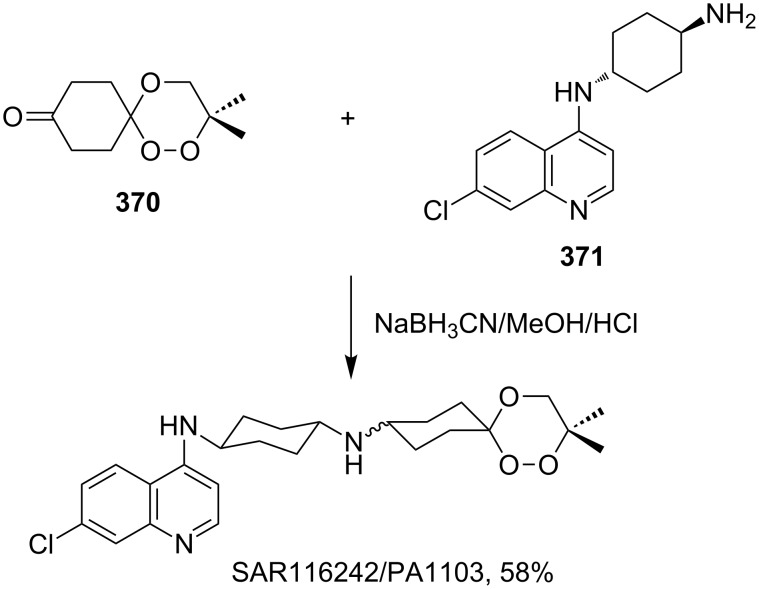
Reductive amination of keto-containing 1,2,4-trioxane **370**.

Trioxaquines are hybrid compounds containing the 1,2,4-trioxane and aminoquinoline moieties. They attracted interest because of a dual mode of action on Plasmodium. One of these compounds, PA1103/SAR116242, was selected as a drug candidate. The final step of its synthesis involves the reductive amination of keto-containing 1,2,4-trioxane **370** with *N*^1^-(7-chloroquin-4-yl)cyclohexane-1,4-diamine (**371**) (Scheme 110) [86].

**Scheme 111:**
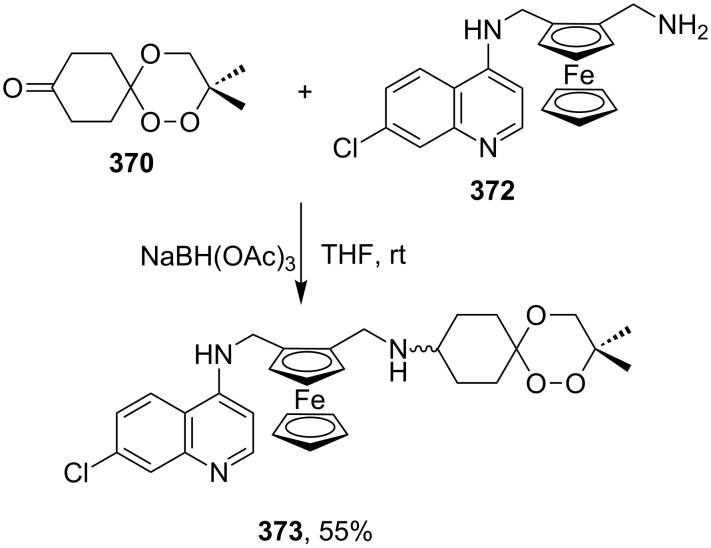
Reductive amination of keto-containing 1,2,4-trioxane and a Fe-containing moiety.

Trioxaferroquines, ferrocene-containing compounds, belong to a new type of hybrid molecules exhibiting high antimalarial activity. The last step of the synthesis of one of these compounds (**373**) based on the reductive amination of ketone **370** with amine **372** is shown in Scheme 111. The unusual fact is that compound **373** bearing the peroxide bond and a Fe-containing moiety is stable [412].

#### 6. Synthesis fo 1,2,4,5-tetraoxanes

The most widely used approaches to the synthesis of 1,2,4,5-tetraoxanes are based on the reaction of ketones and aldehydes with hydrogen peroxide or *gem*-bishydroperoxides catalyzed by protic or aprotic acids, MeReO_3_, Re_2_O_7_, and iodine. These methods were used for the synthesis of a wide range of symmetrical and unsymmetrical 1,2,4,5-tetraoxanes.

#### 6.1. Acid-catalyzed cyclocondensation of ketones and aldehydes with hydrogen peroxide

**Scheme 112:**
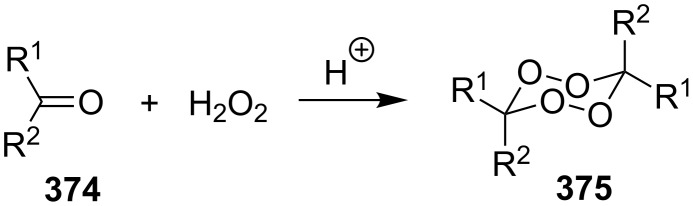
Acid-catalyzed reactions of Н_2_О_2_ with ketones and aldehydes **374**.

This cyclocondensation is the simplest route to some symmetrical (containing identical substituents in positions 3 and 6) 1,2,4,5-tetraoxanes **375** starting from ketones **374** (Scheme 112, Table 24). The acid-catalyzed reactions of hydrogen peroxide with dialkyl ketones, cycloalkanones, and substituted medium-size cycloalkanones produce symmetrical 1,2,4,5-tetraoxanes in moderate to high yields. The drawback of this method is the high sensitivity of the yields of the target peroxides to the structure of the starting carbonyl compounds.

**Table 24:** Examples of symmetrical 1,2,4,5-tetraoxanes **375** synthesized by the acid-catalyzed cyclocondensation of ketones and aldehydes with Н_2_О_2_.

Tetraoxane **375**; R^1^, R^2^	Reaction conditions	Yield, %	Reference

–CH(Et)(CH_2_)_4_–	H_2_O_2_, MeCN, H_2_SO_4_, −20 °С, 48 h	24	[413]
–CH(Pr)(CH_2_)_4_–		7	
–CH_2_CH(Me)(CH_2_)_3_–		32	
–CH(Me)CH(Me)(CH_2_)_3_–		4	
–CH(Me)CH_2_CH(Me)(CH_2_)_2_–		20	
–CH(Me)(CH_2_)_2_CH(Me)CH_2_–		26	
–(CH_2_)_2_C(Me)_2_(CH_2_)_2_–		29	
–CH(iPr)(CH_2_)_2_CH(Me)CH_2_–		20	
–CH(Me)(CH_2_)_2_CH(iPr)CH_2_–		26	
–CH(Me)CH_2_C(Me)_2_(CH_2_)_2_–		34	
–CH_2_C(Me)_2_CH_2_C(Me)_2_CH_2_–		68	
–C(Me)_2_(CH_2_)_4_–		26	[414]
–CH(Me)(CH_2_)_3_CH(Me)–	H_2_O_2_, EtOH/H_2_O, H_2_SO_4_, 0 °С	18	
–(CH_2_)_5_–		90	
–(CH_2_)_5_–	H_2_O_2_, (CF_3_)_2_CHOH	≈100	[415]
H, –(CH_2_)_3_CHO	H_2_O_2_, EtOH/H_2_O, H_2_SO_4_, −10°C, 1 h	80	[416]

#### 6.2. Use of the bis(trimethylsilyl)peroxide/trimethylsilyltrifluoromethanesulfonate system in the cyclocondensation of carbonyl compounds

**Scheme 113:**
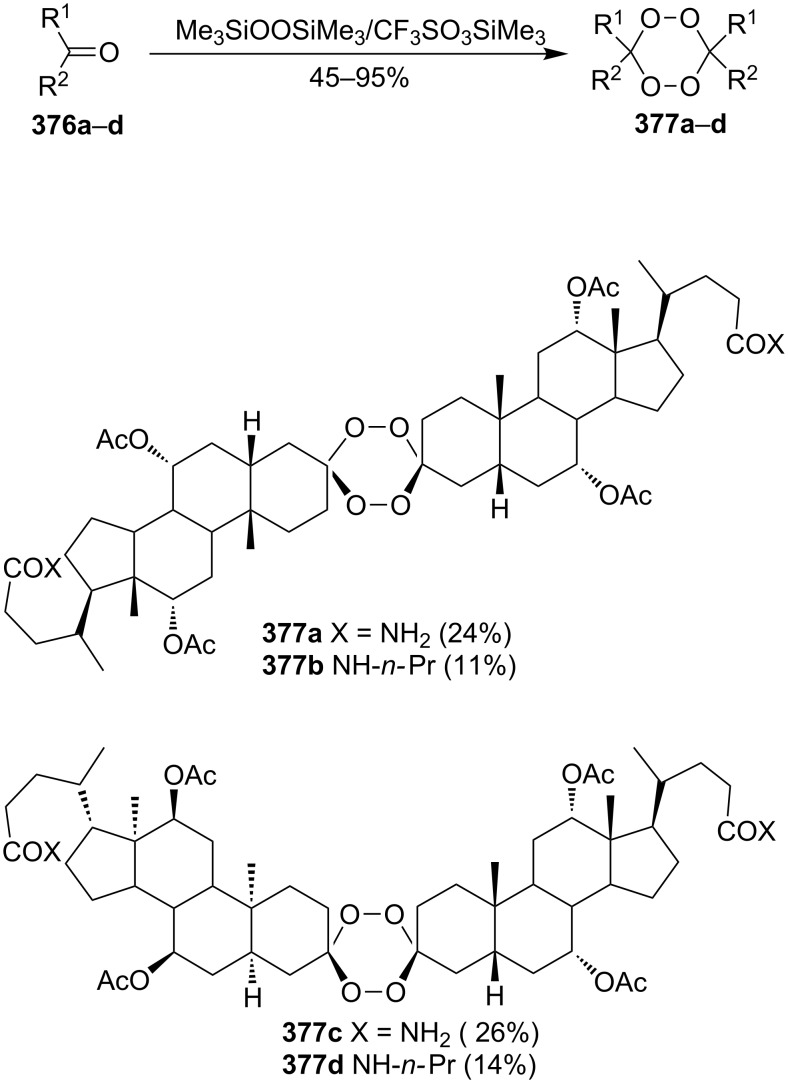
Cyclocondensation of carbonyl compounds **376a–d** using Me_3_SiOOSiMe_3_/CF_3_SO_3_SiMe_3_.

The cyclocondensation of carbonyl compounds **376a–d** with Me_3_SiOOSiMe_3_/CF_3_SO_3_SiMe_3_ afforded steroidal tetraoxanes **377a–d** (Scheme 113) [417–418]. The cyclocondensation of ketones **376** was performed at 0 °С in acetonitrile using a 1.5-fold molar excess of Me_3_SiOOSiMe_3_ and CF_3_SO_3_SiMe_3_ with respect to ketone **376** [417–418].

#### 6.3. MeReO_3_-catalyzed peroxidation of ketones

**Scheme 114:**
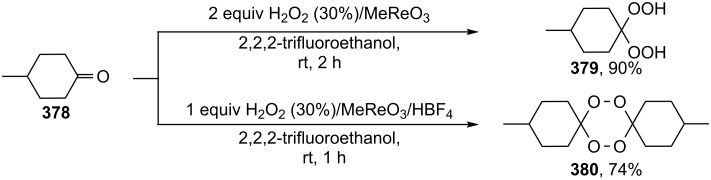
Peroxidation of 4-methylcyclohexanone (**378**).

1,1-Dihydroperoxy-4-methylcyclohexane (**379**) and symmetrical tetraoxane **380** were selectively synthesized in high yields from 4-methylcyclohexanone (**378**) with the use of the 30% Н_2_О_2_/MeReO_3_/fluorinated alcohol system (Scheme 114) [419].

**Scheme 115:**
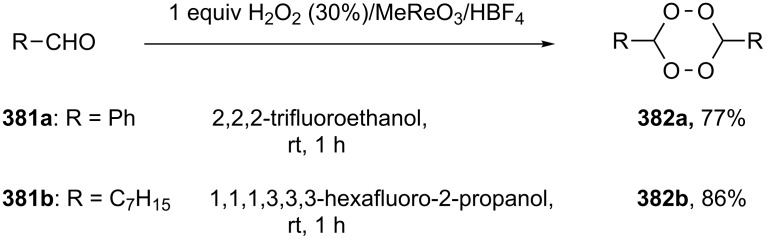
Synthesis of symmetrical tetraoxanes **382a**,**b** from aldehydes **381a**,**b**.

The use of fluorinated alcohols as the solvent results in an increase in the selectivity of the synthesis. Under similar conditions, symmetrical 3,6-diphenyl- and 3,6-di-(*n*-heptyl)-1,2,4,5-tetraoxanes **382a**,**b** were synthesized from benzaldehyde (**381a**) and *n*-octanal (**381b**), respectively (Scheme 115) [419].

**Scheme 116:**
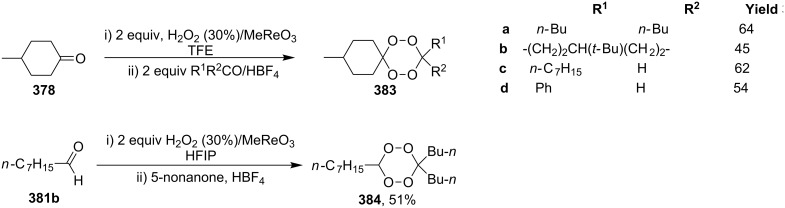
Synthesis of unsymmetrical tetraoxanes using of MeReO_3_.

Unsymmetrical tetraoxanes **383a–d** were prepared from 4-methylcyclohexanone (**378**) by the reaction with ketones (R^1^COR^2^) using of 1 equiv of HBF_4_, 2 equiv of Н_2_О_2_, and 0.1 mol % MeReO_3_ in CF_3_CH_2_OH (TFE) at room temperature. The unsymmetrical tetraoxane, 3,3-dibutyl-6-heptyl-1,2,4,5-tetraoxane (**384**), was synthesized from octanal (**381b**) with the use of CH_3_CHOHCF_3_ (HFIP) (Scheme 116) [419].

**Scheme 117:**
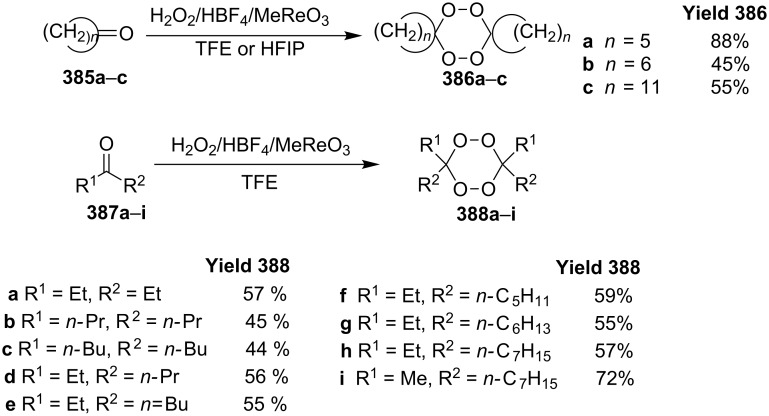
Synthesis of symmetrical tetraoxanes using of MeReO_3_.

**Scheme 118:**
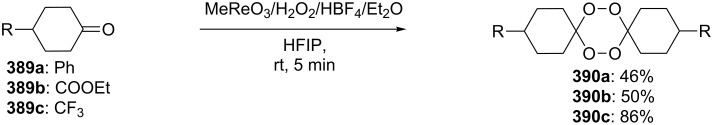
Synthesis of symmetrical tetraoxanes using of MeReO_3_.

This method was applied to the synthesis of 3,3,6,6-tetraalkyl-1,2,4,5-tetraoxanes **386a–c** and **388a–i** from cyclic **385a–c** and acyclic ketones **387a–i** (Scheme 117) [420], as well as of dispiro-1,2,4,5-tetraoxanes **390a–c** from 4-substituted cyclohexanones **389a–c** (Scheme 118) [421].

**Scheme 119:**
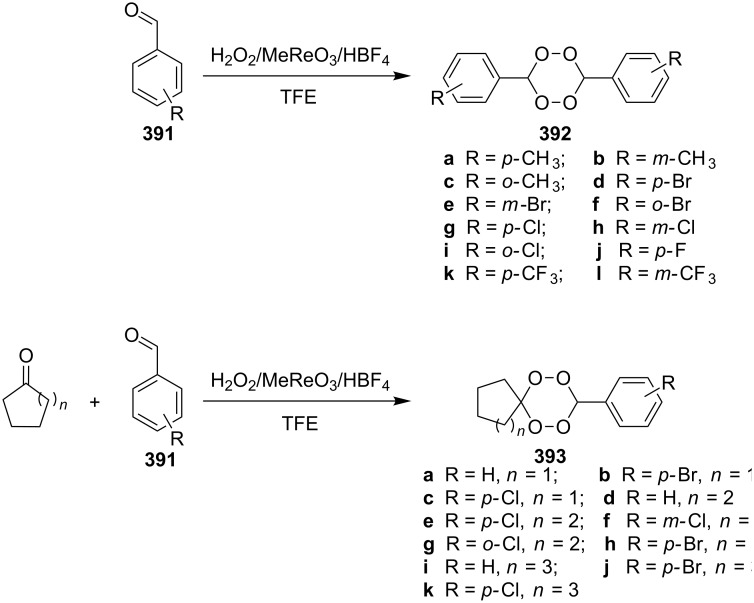
MeReO_3_ in the synthesis of symmetrical tetraoxanes with the use of aldehydes.

The use of the 30% Н_2_О_2_/MeReO_3_ (МТО)/fluorinated alcohol system enabled the synthesis of symmetrical compounds **392** from aldehydes **391** and unsymmetrical tetraoxanes **393** containing aryl (peroxide-destabilizing) substituents from aldehydes **391** (and cycloalkanones) (Scheme 119) [422].

**Scheme 120:**
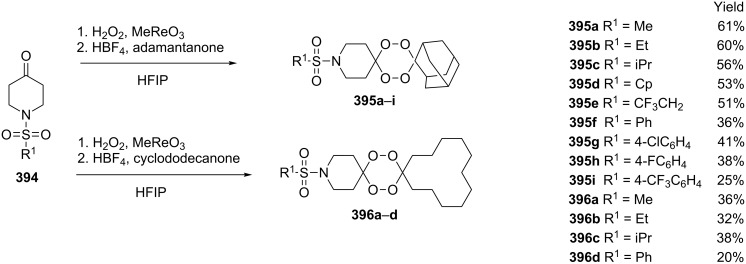
Preparation of unsymmmetrical 1,2,4,5-tetraoxanes with high antimalarial activity.

Unsymmmetrical 1,2,4,5-tetraoxanes containing adamantane (**395a–i**) and cyclodecane moieties (**396a–d**) exhibiting high antimalarial activity (Scheme 120) were prepared from sulfonylpiperidones **394** [138].

#### 6.4. Re_2_O_7_-Catalyzed cyclocondensation of *gem-*bishydroperoxides with ketones

**Scheme 121:**
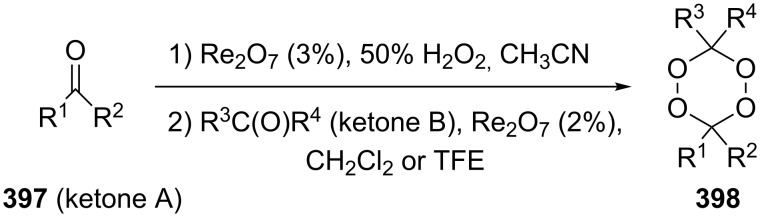
Re_2_O_7_-Catalyzed synthesis of tetraoxanes **398**.

Re_2_O_7_ is an efficient catalyst for the addition of hydroperoxide groups to ketones and aldehydes. Due to these properties, Re_2_O_7_ can be used in the one-pot synthesis of unsymmetrical 1,2,4,5-tetraoxanes **398** from ketones **397** in good yields (Scheme 121, Table 25) [423].

**Table 25:** Re_2_O_7_-Catalyzed synthesis of tetraoxanes **398**.

Ketone A, **397**	Ketone B	Reaction conditions	Tetraoxane **398**	Yield, %

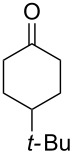	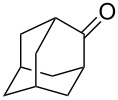	1) Н_2_О_2_ (2 equiv), 0.5 h, 0 °С 2) Ketone B (2 equiv), CH_2_Cl_2_, 1 h, rt	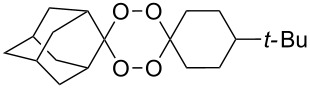	67
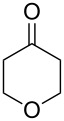	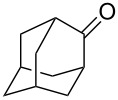	1) Н_2_О_2_ (4 equiv), 0.5 h, rt 2) Ketone B (4 equiv), 2,2,2-trifluoroethanol, Re_2_O_7_, 0.5 h, rt	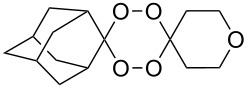	69
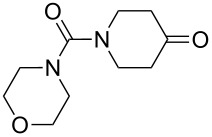	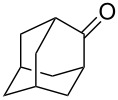	1) Н_2_О_2_ (4 equiv), 6 h, rt 2) Ketone B (4 equiv), 2,2,2-trifluoroethanol, Re_2_O_7_, 2 h, rt	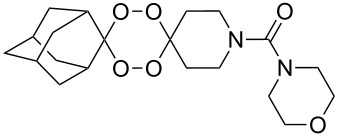	49

#### 6.5. Protic acid-catalyzed cyclocondensation of *gem-*bishydroperoxides with ketones

**Scheme 122:**
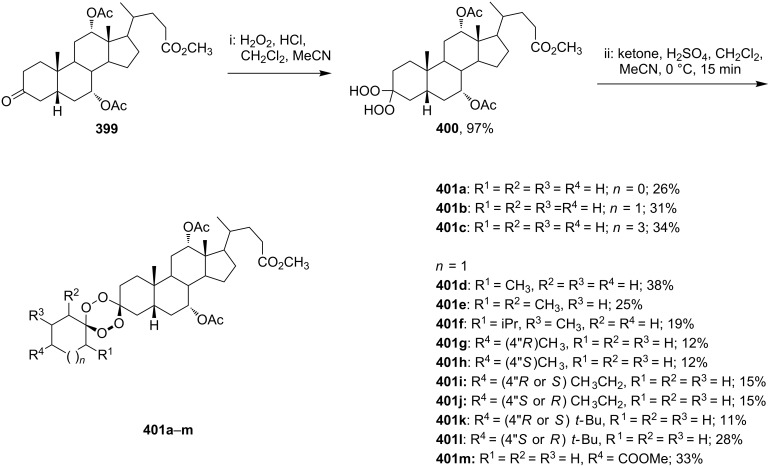
H_2_SO_4_-Catalyzed synthesis of steroidal tetraoxanes **401**.

Unsymmetrical steroidal tetraoxanes **401** were synthesized by the hydroperoxidation of methyl 3-oxo-7α,12α-diacetoxy-5β-cholan-24-oate (**399**) in the presence of HCl followed by the condensation of bishydroperoxide **400** with the corresponding ketone in the presence of H_2_SO_4_ (Scheme 122) [128,132,141–142].

Structurally more simple ketones, for example, acetone, are also involved in the cyclocondensation with bishydroperoxide **400** [141].

**Scheme 123:**
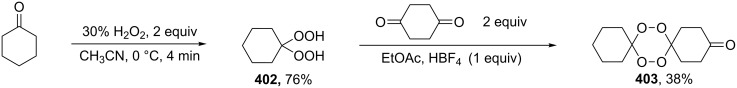
HBF_4_-Catalyzed condensation of bishydroperoxide **402** with 1,4-cyclohexanedione.

The synthesis of keto-containing tetraoxane **403** was also performed in two steps [144]. Thus the intermediate 1,1-dihydroperoxycyclohexane **402** was prepared from cyclohexanone in a neutral medium, and its condensation with 1,4-cyclohexanedione was carried out in the presence of HBF_4_ (Scheme 123).

#### 6.6. Cyclocondensation of bishydroperoxides with acetals and enol ethers

**Scheme 124:**
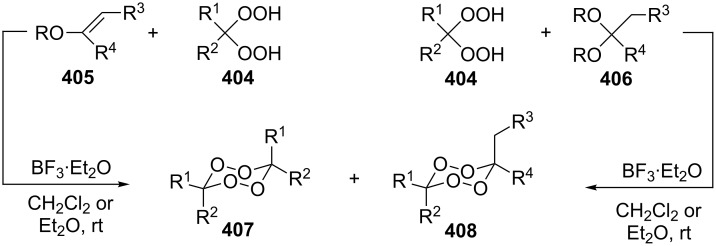
BF_3_·Et_2_O-Catalyzed reaction of *gem-*bishydroperoxides **404** with enol ethers **405** and acetals **406**.

The method for the synthesis of 1,2,4,5-tetraoxanes **407** and **408** is based on the boron trifluoride etherate-catalyzed reaction of *gem-*bishydroperoxides **404** with enol ethers **405** and acetals **406** under mild conditions. More than two dozens of tetraoxanes were synthesized in yields from 45 to 95% according to this method (Scheme 124). The advantage of this method is the use of readily available starting compounds, such as acetals, enol ethers, and boron trifluoride etherate [424–425].

**Scheme 125:**
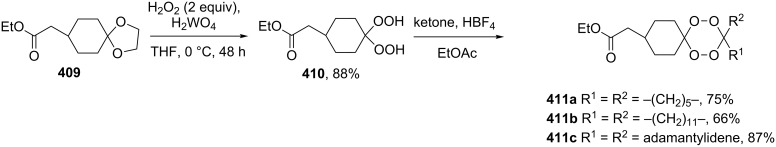
HBF_4_-Catalyzed cyclocondensation of bishydroperoxide **410** with ketones.

The bishydroperoxidation of 1,3-dioxolane **409** was carried out in the presence of H_2_WO_4_. The following HBF_4_-catalyzed condensation of bishydroperoxide **410** with ketones gave 1,2,4,5-tetraoxanes **411a–c** containing the ester group (Scheme 125) [144].

#### 6.7. Iodine-catalyzed one-pot synthesis of symmetrical and unsymmetrical tetraoxanes

**Scheme 126:**
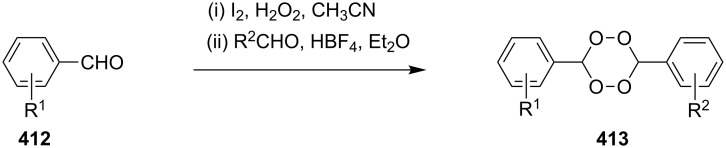
Synthesis of symmetrical and unsymmetrical tetraoxanes **413** from benzaldehydes **412**.

The reaction of substituted benzaldehyde **412** with hydrogen peroxide in the presence of the Lewis acid I_2_ produced geminal bishydroperoxide, whose condensation with the starting or another substituted benzaldehyde gave tetraoxane **413** (Scheme 126, Table 26) [426–427].

**Table 26:** Iodine-catalyzed one-pot synthesis of tetraoxanes **413**.

Tetraoxane **413**		Yield, %	Tetraoxane **413**		Yield, %
	
R^1^	R^2^		R^1^	R^2^	

*o-*Me	*o-*Me	42	*p-(t-*Bu)	*p-(n-*Pr)	32
*o-*Me	*m-*Me	33	*p-(t-*Bu)	*p-*(iPr)	38
*p-*Me	*p-*Me	54	*p-(n-*Pr)	*p-*(iPr)	28
*p-*Me	*p-*(iPr)	33	*p-(t-*Bu)	*p-*OMe	22
*p-*Me	*p-(t-*Bu)	46	*p-*(iPr)	*p-*OMe	24
*p-*Me	*p-*OMe	25	*p-*Et	*p-*Me	41
*p-*Me	*p-*CO_2_Me	37	*p-*Et	*m-*Me	39
*p-*Me	*o-*Me	25	*p-*Et	*p-*(iPr)	37
*p-*Me	*m-*Me	38	*p-*Et	*p-(t-*Bu)	25
*p-*Me	*p-(n-*Pr)	37	*p-(n-*Pr)	*p-*OMe	24
*p-*Me	H	43	*p-*Cl	*p-*Cl	25
*p-*Me	*p-*CHO	31	*p-*Br	*p-*Br	22
*p-(n-*Bu)	*p-(n-*Bu)	40	*p-*F	*p-*F	29
*p-(t-*Bu)	*p-(t-*Bu)	53	*p-*OMe	*p-*OMe	27
*m-*Me	*m-*Me	51	*p-*Et	*p-*Et	44
*m-*Me	H	30	*p-(n-*Pr)	*p-(n-*Pr)	38
*m-*Me	*p-*OMe	29	*p-* (iPr)	*p-*(iPr)	41

The iodine-catalyzed one-pot synthesis of symmetrical and unsymmetrical tetraoxanes from substituted benzaldehydes has some advantages over other methods. Thus, it can be performed with the use of mild reagents (which do not decompose peroxide) and it does not need an excess of hydrogen peroxide and substituted benzaldehyde.

#### 6.8. Acid-catalyzed condensation of β-diketones with hydrogen peroxide

**Scheme 127:**
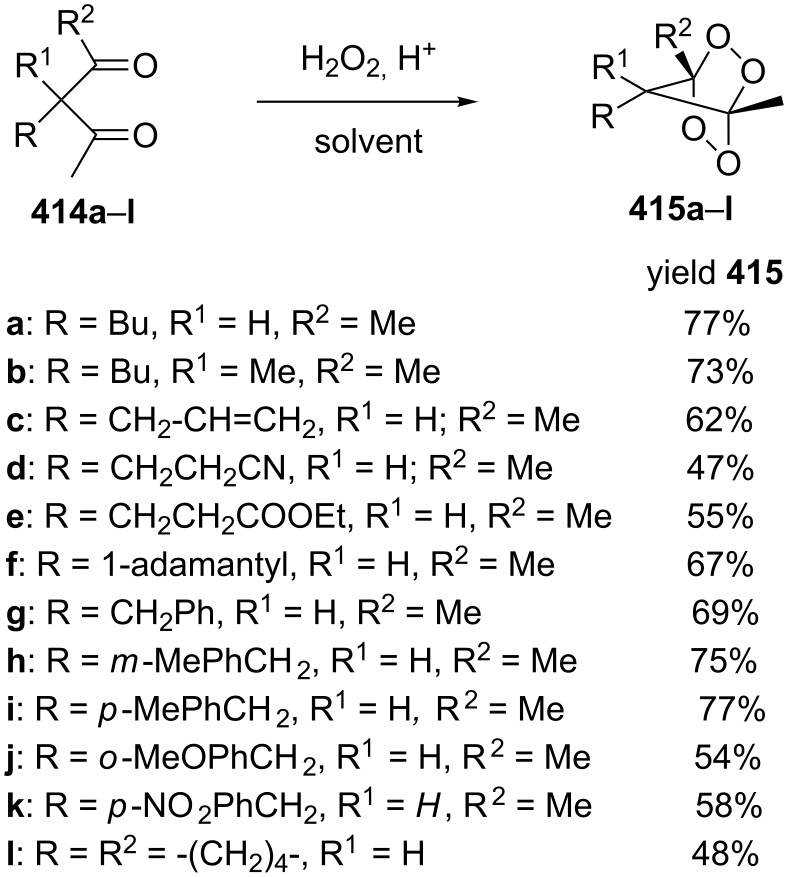
Synthesis of bridged 1,2,4,5-tetraoxanes **415a–l** from β-diketones **414a–l** and H_2_O_2_.

The acid-catalyzed condensation of β-diketones **414a–l** with hydrogen peroxide is a simple and facile method for the synthesis of bridged 1,2,4,5-tetraoxanes **415a–l**. This method enables the synthesis of these compounds on the multigram scale in 47–77% yields (Scheme 127). The high concentration of a strong acid, such as H_2_SO_4_, HBF_4_, or HClO_4_ (2 g of the acid per 5 mL of the solvent) is the key factor determining the yield and selectivity of the synthesis of 1,2,4,5-tetraoxanes. Under these conditions, the targeted compounds are produced selectively even in the presence of an excess of hydrogen peroxide [428]. Unlike many compounds with the O–O bond, which are rearranged in acidic media, the resulting cyclic peroxides are fairly stable under these reaction conditions.

It was found that phosphomolybdic acid and phosphotungstic acid efficiently catalyze the addition of H_2_O_2_ to β-diketones resulting in the selective formation of bridged 1,2,4,5-tetraoxanes. The use of these catalysts made it possible to obtain bridged tetraoxanes from easily oxidizable benzoylacetone derivatives and α-unsubstituted β-diketones [429].

#### 6.9. Synthesis of symmetrical 1,2,4,5-tetraoxanes by the ozonolysis of unsaturated compounds

**Scheme 128:**
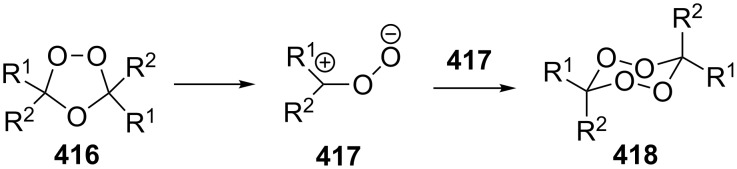
Dimerization of zwitterions **417**.

The dimerization of zwitterions **417** produced by decomposition of ozonides **416** affords symmetrical tetraoxanes **418** (Scheme 128).

**Scheme 129:**
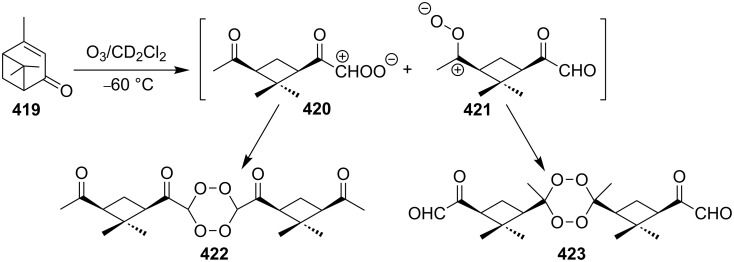
Ozonolysis of verbenone **419**.

For example, the ozonolysis of verbenone **419** via the formation of zwitterioninc structures **420** and **421** gives a mixture of two symmetrical 1,2,4,5-tetraoxanes **422** and **423** (Scheme 129) [430]. Peroxides **422** and **423** are unstable due to the presence of carbonyl groups adjacent to the O–O group, and they almost completely decompose as the temperature is raised.

**Scheme 130:**
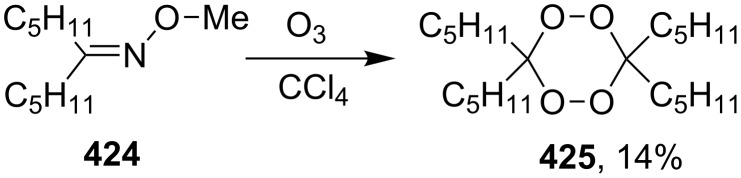
Ozonolysis of O-methyl oxime **424**.

3,3,6,6-Tetrapentyl-1,2,4,5-tetraoxane (**425**) was synthesized in a similar way by the ozonolysis of undecan-6-one O-methyl oxime (**424**) (Scheme 130) [431–432]. It should be noted that this approach is not widely used because of a limited number of appropriate structures and low yields of the target products.

#### 6.10. Other methods for the synthesis of 1,2,4,5-tetraoxanes

**Scheme 131:**
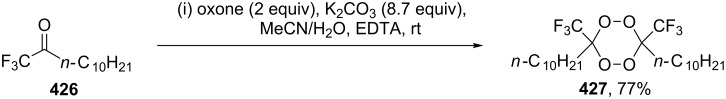
Peroxidation of 1,1,1-trifluorododecan-2-one **426** with oxone.

The peroxidation of 1,1,1-trifluorododecan-2-one (**426**) with oxone afforded the symmetrical tetraoxane, 3,6-didecyl-3,6-bis(trifluoromethyl)-1,2,4,5-tetraoxane (**427**) (Scheme 131) [433].

**Scheme 132:**
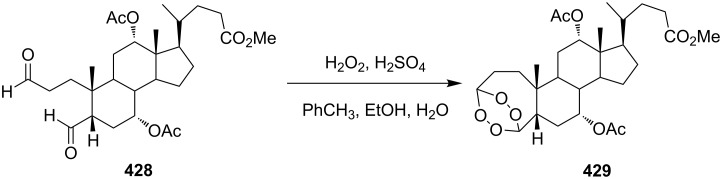
Intramolecular cyclization of dialdehyde **428** with H_2_O_2_.

The synthesis of unsymmetrical steroidal tetraoxane **429** in 19% yield was performed by the intramolecular cyclization of dialdehyde **428** with hydrogen peroxide under acidic conditions (Scheme 132) [434].

**Scheme 133:**
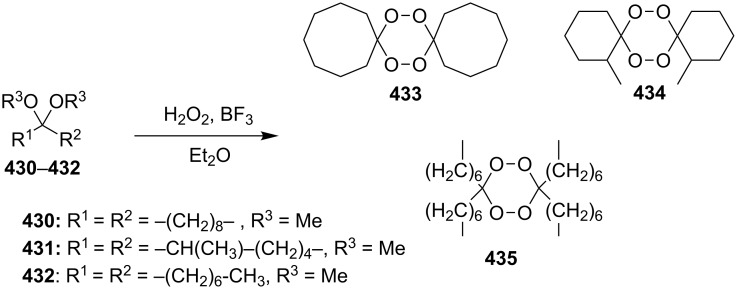
Tetraoxanes **433**–**435** as by-products in peroxidation of ketals **430–432**.

In the synthesis of geminal bishydroperoxides by BF_3_·Et_2_O or BF_3_·MeOH-catalyzed reactions of ketals **430–432** with hydrogen peroxide in Et_2_O tetraoxanes **433–435** (Scheme 133) are obtained as by-products in 12%, 6%, and 19% yields, respectively [435].

**Scheme 134:**
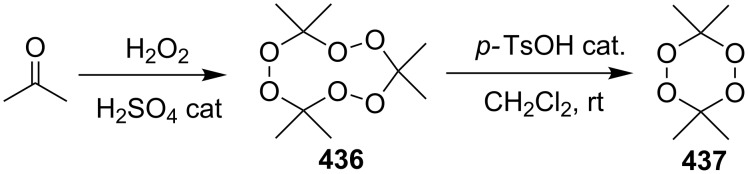
Transformation of triperoxide **436** in diperoxide **437**.

Scheme 134 shows the synthesis of 3,3,6,6-tetramethyl-1,2,4,5-tetraoxane (**437**) in 90% yield by the transformation of the intermediate 3,3,6,6,9,9-hexamethyl-1,2,4,5,7,8-hexaoxononane (**436**) in acetone [436]. This method is suitable for the preparation of the target product in amounts of only several hundred milligrams.

#### 6.11. Structural modifications, in which 1,2,4,5-tetraoxane ring remains intact

In the last two decades, 1,2,4,5-tetraoxanes were considered as the most promising compounds for the design of antiparasitic drugs. This is due, first, to the high activity of their derivatives and, second, to a wide scope of structural modifications, in which the tetraoxane ring remains intact.

**Scheme 135:**
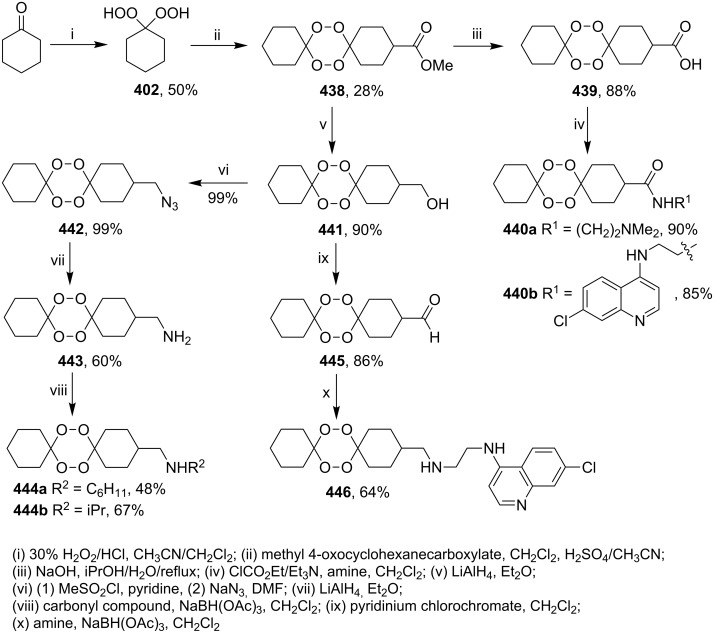
Preparation and structural modifications of tetraoxanes.

Amides **440a**,**b** and amines **444a**,**b**, and **446** active against various strains of *P.falciparum* were synthesized from methyl 7,8,15,16-tetraoxadispiro[5.2.5.2]hexadecane-3-carboxylate (**438**) containing the ester group (Scheme 135) [135,437]. To prepare aminoquinoline derivatives **440a**,**b**, ester **438** was hydrolyzed to 7,8,15,16-tetraoxadispiro[5.2.5.2]hexadecane-3-carboxylic acid (**439**) followed by the amidation of the latter. The synthesis of products **444a**,**b** and **446** was performed with a wide range of classical reagents for organic synthesis with the intermediate formation of compounds containing such groups as hydroxy **441**, azide **442**, amino **443**, and aldehyde **445**.

An interesting feature of the synthesis according to Scheme 135 is the use of such strong reducing agents as LiAlH_4_ and NaBH(OAc)_3_, with the products retaining the peroxide ring.

**Scheme 136:**
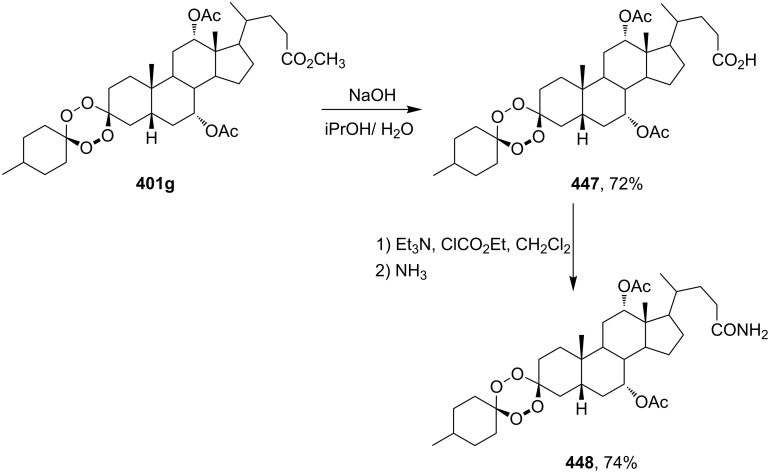
Structural modifications of steroidal tetraoxanes.

Steroidal tetraoxane **448**, which is approximately six times more active that Artelinic acid and 2.4 times as active as arteether against *P. falciparum*, was also synthesized by the alkaline hydrolysis of ester **401g** followed by the amidation of acid **447** (Scheme 136) [128].

**Scheme 137:**
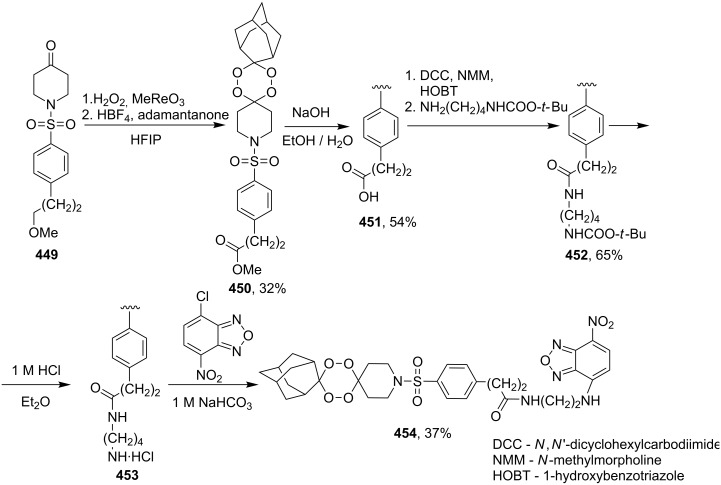
Synthesis of 1,2,4,5-tetraoxane **454** containing the fluorescent moiety.

Compounds containing a fluorescent moiety are of interest in terms of the mechanism of antiparasitic action of peroxides. For example, 1,2,4,5-tetraoxane **454** containing the 4-chloro-7-methylbenzo[*c*][1,2,5]oxadiazole moiety was synthesized according to Scheme 137. In the first step, ketone **449** was transformed in tetraoxane **450**, whose ester group was subjected to the alkaline hydrolysis to form acid **451** followed by the amidation to give **452** and the hydrolysis to obtain hydrochloride **453**. Then the reaction of the latter with 4-chloro-7-nitrobenzo[*c*][1,2,5]oxadiazole afforded the target compound **454** [138].

**Scheme 138:**
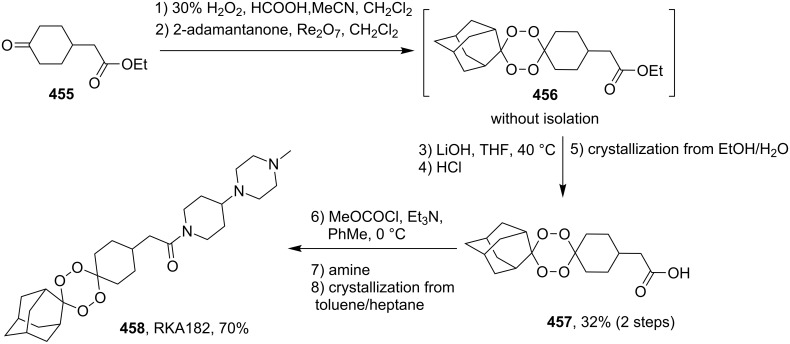
Synthesis of tetraoxane **458** (RKA182).

The synthesis of tetraoxane **458** (RKA182) exhibiting the in vitro and in vivo activity comparable with that of artemisinin was performed on the kilogram scale according to Scheme 138. This compound is a promising malaria drug candidate [82–83]. The key steps in this synthesis are the preparation of adamantane-containing tetraoxane **456** from ethyl 2-(4-oxocyclohexyl)acetate (**455**), the hydrolysis of **456**, and the purification to obtain acid **457**. The amidation of the latter affords target product **458**.

## Conclusion

The review summarizes and generalizes studies on the synthesis of five- and six-membered cyclic peroxides published last decade (since 2000 to present). Most of the currently established methods for the synthesis of these compounds are based on the use of such key oxidizing agents as oxygen, ozone, and hydrogen peroxide. The Isayama–Mukaiyama and Kobayashi methods are widely used in the synthesis of 1,2-dioxolanes, 1,2-dioxanes, and 1,2,4-trioxanes. The reactions with the participation of peroxycarbenium ions play an important role in the synthesis of peroxides.

The Griesbaum coozonolysis of ketones and O-alkyl oximes is the most flexible and efficient method for the synthesis of unsymmetrical 1,2,4-trioxolanes. The [4 + 2]-cycloaddition of oxygen to a 1,3-diene system is, in fact, the only route to 1,2-dioxenes.

Methods for the synthesis of 1,2,4,5-tetraoxanes are based on reactions of ketones, aldehydes, and their dialkyloxy derivatives with hydrogen peroxide or *gem*-bishydroperoxides catalyzed by protic and aprotic acids, such as MeReO_3_, Re_2_O_7_, and iodine.

Modifications of functional groups to form peroxide ring-retaining products are applicable to the synthesis of cyclic peroxides of various structural types. This approach can be used to prepare complex peroxides exhibiting antiparasitic and antitumor activities.

Carbonyl compound are generally employed as the starting reagents in the synthesis of cyclic peroxides. These methods can be used for the selective peroxidation of monocarbonyl compounds. In the case of dicarbonyl compounds, there are a limited number of efficient procedures for the synthesis of cyclic peroxides.

## References

[1] McCullough K J, Nojima M (2001). Curr Org Chem.

[2] Korshin E E, Bachi M D, Rappoport Z (2006). Synthesis of cyclic peroxides. The Chemistry of Peroxides.

[3] Tang Y, Dong Y, Vennerstrom J L (2004). Med Res Rev.

[4] O’Neill P M, Posner G H (2004). J Med Chem.

[5] Slack R D, Jacobine A M, Posner G H (2012). Med Chem Commun.

[6] Masuyama A, Wu J-M, Nojima M, Kim H-S, Wataya Y (2005). Mini-Rev Med Chem.

[7] Van Ornum S G, Champeau R M, Pariza R (2006). Chem Rev.

[8] Jefford C W (2007). Drug Discovery Today.

[9] Jefford C W (2012). Curr Top Med Chem.

[10] Dembitsky V M (2008). Eur J Med Chem.

[11] Siddiq A, Dembitsky V (2008). Anti-Cancer Agents Med Chem.

[12] Dembitsky V M, Levitsky D O (2006). Nat Prod Commun.

[13] Dembitsky V M (2006). Lipids.

[14] Dembitsky V M (2005). Lipids.

[15] Dembitsky V M (2004). Chem Biodiversity.

[16] Opsenica D M, Šolaja B A (2009). J Serb Chem Soc.

[17] Muraleedharan K M, Avery M A (2009). Drug Discovery Today.

[18] Kim B J, Sasaki T (2006). Org Prep Proced Int.

[19] Baby J, Sujatha S (2011). J Nat Prod (Gorakhpur, India).

[20] Hepworth J D, Heron B M (1996). Prog Heterocycl Chem.

[21] Kabal'nova N N, Khursan S L, Shereshovets V V, Tolstikov G A (1999). Kinet Catal.

[22] Terent'ev A O, Platonov M M, Levitsky D O, Dembitsky V M (2011). Russ Chem Rev.

[23] Baader W J, Bastos E L (2008). Sci Synth.

[24] Baader W J, Bastos E L (2008). Sci Synth.

[25] Baader W J, Bastos E L (2008). Sci Synth.

[26] Kumar N, Singh R, Rawat D S (2012). Med Res Rev.

[27] Zmitek K, Zupan M, Iskra J (2007). Org Biomol Chem.

[28] Trullinger T K (2002).

[29] Dai P (2004).

[30] Ramirez A P (2007).

[31] Pena-Quevedo A J (2009).

[32] Yao G (1999).

[33] Morgan N N (2009). Int J Phys Sci.

[34] Odinokov V N, Tolstikov G A (1981). Russ Chem Rev.

[35] Razumovskii S D, Zaikov G E (1980). Russ Chem Rev.

[36] Schmidt-Szalowski K (2000). Przem Chem.

[37] Capon R J (1991). Stud Nat Prod Chem.

[38] Cafferata L F R (1993). Trends Org Chem.

[39] Tolstikov G A, Tolstikov A G, Tolstikova O V (1996). Russ Chem Rev.

[40] McCullough K J (1995). Contemp Org Synth.

[41] Adam W (1979). Acc Chem Res.

[42] Opsenica D M, Šolaja B A (2012). Maced J Chem Chem Eng.

[43] Sawwan N, Greer A (2007). Chem Rev.

[44] Clennan E L, Foote C, Ando E (1992). Organic Peroxides.

[45] Balci M (1981). Chem Rev.

[46] Clennan E L (1991). Tetrahedron.

[47] Doerig C D (2008). Nat Chem Biol.

[48] Vangapandu S, Jain M, Kaur K, Patil P, Patel S R, Jain R (2007). Med Res Rev.

[49] Tu Y (2011). Nat Med (N Y, NY, U S).

[50] Kumar N, Sharma M, Rawat D S (2011). Curr Med Chem.

[51] Liao F (2009). Molecules.

[52] Morris C A, Duparc S, Borghini-Fuhrer I, Jung D, Shin C-S, Fleckenstein L (2011). Malar J.

[53] Robert A, Dechy-Cabaret O, Cazalles J, Meunier B (2002). Acc Chem Res.

[54] Hombhanje F W, Huang Q (2010). Pharmaceuticals.

[55] Kano S (2010). J Infect Chemother.

[56] Li Q, Weina P (2010). Pharmaceuticals.

[57] Wright C W (2010). Nat Prod Rep.

[58] Kaur H, Green M D, Hostetler D M, Fernandez F M, Newton P N (2010). Therapy.

[59] Medhi B, Patyar S, Rao R S, Byrav D S P, Prakash A (2009). Pharmacology.

[60] Eastman R T, Fidock D A (2009). Nat Rev Microbiol.

[61] Ehrhardt S, Meyer C G (2009). Ther Clin Risk Manage.

[62] Gautam A, Ahmed T, Paliwal J, Batra V (2009). Curr Drug Metab.

[63] Bathurst I, Hentschel C (2006). Trends Parasitol.

[64] White N J (2008). Science.

[65] Nakase I, Lai H, Singh N P, Sasaki T (2008). Int J Pharm.

[66] Nosten F, White N J (2007). Am J Trop Med Hyg.

[67] Stocks P A, Bray P G, Barton V E, Al-Helal M, Jones M, Araujo N C, Gibbons P, Ward S A, Hughes R H, Biagini G A (2007). Angew Chem, Int Ed.

[68] Checkley A M, Whitty C J M (2007). Expert Rev Anti-Infect Ther.

[69] Tilley L, Davis T M E, Bray P G (2006). Future Microbiol.

[70] Gelb M H (2007). Curr Opin Chem Biol.

[71] Namdeo A G, Mahadik K R, Kadam S S (2006). Pharm Mag.

[72] Haynes R K (2006). Curr Top Med Chem (Sharjah, United Arab Emirates).

[73] Begue J-P, Bonnet-Delpon D (2005). Drugs Future.

[74] Rosenthal A S, Chen X, Liu J O, West D C, Hergenrother P J, Shapiro T A, Posner G H (2009). J Med Chem.

[75] Nagelschmitz J, Voith B, Wensing G, Roemer A, Fugmann B, Haynes R K, Kotecka B M, Rieckmann K H, Edstein M D (2008). Antimicrob Agents Chemother.

[76] Van der Meersch H (2005). J Pharm Belg.

[77] Vennerstrom J L, Arbe-Barnes S, Brun R, Charman S A, Chiu F C K, Chollet J, Dong Y, Dorn A, Hunziker D, Matile H (2004). Nature.

[78] Valecha N, Looareesuwan S, Martensson A, Abdulla S M, Krudsood S, Tangpukdee N, Mohanty S, Mishra S K, Tyagi P K, Sharma S K (2010). Clin Infect Dis.

[79] Uhlemann A C, Wittlin S, Matile H, Bustamante L Y, Krishna S (2007). Antimicrob Agents Chemother.

[80] Gupta A, Singh Y, Srinivas K S, Jain G, Sreekumar V B, Semwal V P (2010). J Pharm BioAllied Sci.

[81] Dong Y, Wittlin S, Sriraghavan K, Chollet J, Charman S A, Charman W N, Scheurer C, Urwyler H, Santo Tomas J, Snyder C (2010). J Med Chem.

[82] O’Neill P M, Amewu R K, Nixon G L, ElGarah F B, Mungthin M, Chadwick J, Shone A E, Vivas L, Lander H, Barton V (2010). Angew Chem, Int Ed.

[83] Bousejra-El Garah F, Wong M H-L, Amewu R K, Muangnoicharoen S, Maggs J L, Stigliani J-L, Park B K, Chadwick J, Ward S A, O'Neill P M (2011). J Med Chem.

[84] Posner G H, O’Neill P M (2004). Acc Chem Res.

[85] Sonnet P, Mullié C (2011). Exp Parasitol.

[86] Coslédan F, Fraisse L, Pellet A, Guillou G, Mordmüller M, Kremsner P, Moreno A, Mazier D, Maffrand J-P, Meunier B (2008). Proc Natl Acad Sci U S A.

[87] Meunier B, Robert A (2010). Acc Chem Res.

[88] Martyn D C, Ramirez A P, Beattie M J, Cortese J F, Patel V, Rush M A, Woerpel K A, Clardy J (2008). Bioorg Med Chem Lett.

[89] Schiaffo C E, Rottman M, Wittlin S, Dussault P H (2011). ACS Med Chem Lett.

[90] Wang X, Dong Y, Wittlin S, Creek D, Chollet J, Charman S A, Santo Tomas J, Scheurer C, Snyder C, Vennerstrom J L (2007). J Med Chem.

[91] Hartwig C L, Lauterwasser E M W, Mahajan S S, Hoke J M, Cooper R A, Renslo A R (2011). J Med Chem.

[92] Dong Y, Chollet J, Matile H, Charman S A, Chiu F C K, Charman W N, Scorneaux B, Urwyler H, Santo Tomas J, Scheurer C (2005). J Med Chem.

[93] Dong Y, Tan Y, Chollet J, Matile H, Wittlin S, Charman S A, Charman W N, Tomas J S, Scheurer C, Snyder C (2006). Bioorg Med Chem.

[94] Padmanilayam M, Scorneaux B, Dong Y, Chollet J, Matile H, Charman S A, Creek D J, Charman W N, Tomas J S, Scheurer C (2006). Bioorg Med Chem Lett.

[95] Yadav G C, Dorwal H N, Tanwar P, Gahlot U B S (2010). A process for the preparation of 1, 2, 4-trioxolane antimalarials. PCT Int. Appl..

[96] Tang Y, Dong Y, Wittlin S, Charman S A, Chollet J, Chiu F C K, Charman W N, Matile H, Urwyler H, Dorn A (2007). Bioorg Med Chem Lett.

[97] Tang Y, Wittlin S, Charman S A, Chollet J, Chiu F C K, Morizzi J, Johnson L M, Santo Tomas J, Scheurer C, Snyder C (2010). Bioorg Med Chem Lett.

[98] Arora V K, Madan S, Trehan A, Tyagi P (2006). Stable dosage forms of spiro and dispiro 1,2,4-trioxolane antimalarials. PCT Int. Appl..

[99] Vennerstrom J L, Dong Y, Chollet J, Matile H, Wang X, Sriraghavan K, Charman W N (2005). Spiro and dispiro 1,2,4-trioxolane antimalarials. U. S. Pat. Appl..

[100] Cazelles J, Cosledan F, Meunier B, Pellet A (2005). Dual molecules containing peroxy derivative, the synthesis and therapeutic applications thereof. Patent Fr. Demande.

[101] Vennerstrom J L, Dong Y, Chollet J, Matile H, Padmanilayam H, Tang Y, Charman N W (2004). Spiro and dispiro 1,2,4-trioxolane antimalarials. U.S. Pat. Appl..

[102] O’Neill P M, Stocks P A, Pugh M D, Araujo N C, Korshin E E, Bickley J F, Ward S A, Bray P G, Pasini E, Davies J (2004). Angew Chem, Int Ed.

[103] Fattorusso C, Persico M, Calcinai B, Cerrano C, Parapini S, Taramelli D, Novellino E, Romano A, Scala F, Fattorusso E (2010). J Nat Prod.

[104] Kawanishi M, Kotoku N, Itagaki S, Horii T, Kobayashi M (2004). Bioorg Med Chem.

[105] Taglialatela-Scafati O, Fattorusso E, Romano A, Scala F, Barone V, Cimino P, Stendardo E, Catalanotti B, Persico M, Fattorusso C (2010). Org Biomol Chem.

[106] Christoffers J, Werner T, Roessle M (2007). Catal Today.

[107] Bachi M D, Korshin E E, Hoos R, Szpilman A M, Ploypradith P, Xie S, Shapiro T A, Posner G H (2003). J Med Chem.

[108] Fattorusso C, Campiani G, Catalanotti B, Persico M, Basilico N, Parapini S, Taramelli D, Campagnuolo C, Fattorusso E, Romano A (2006). J Med Chem.

[109] Murakami N, Kawanishi M, Itagaki S, Horii T, Kobayashi M (2002). Bioorg Med Chem Lett.

[110] Murakami N, Kawanishi M, Mostaqul H M, Li J, Itagaki S, Horii T, Kobayashi M (2003). Bioorg Med Chem Lett.

[111] Liu H-H, Zhang Q, Jin H-X, Shen X, Wu Y-K (2006). Chin J Chem.

[112] Liu H-H, Jin H-X, Zhang Q, Wu Y-K, Kim H-S, Wataya Y (2005). Chin J Chem.

[113] Givelet C, Bernat V, Danel M, André-Barrès C, Vial H (2007). Eur J Org Chem.

[114] Rudi A, Talpir R, Kashman Y, Benayahu Y, Schleyer M (1993). J Nat Prod.

[115] Ruiz J, Tuccio B, Lauricella R, Maynadier M, Vial H, André-Barrès C (2013). Tetrahedron.

[116] Kamata M, Hagiwara J-i, Hokari T, Suzuki C, Fujino R, Kobayashi S, Kim H-S, Wataya Y (2013). Res Chem Intermed.

[117] Najjar F, Gorrichon L, Baltas M, André-Barrès C, Vial H (2005). Org Biomol Chem.

[118] Kondo K, Matsumoto M, Hatsutani M (1974). Patent Jpn. Kokai Tokkyo Koho.

[119] Bernat V, Saffon N, Maynadier M, Vial H, André-Barrès C (2009). Tetrahedron.

[120] Amewu R, Gibbons P, Mukhtar A, Stachulski A V, Ward S A, Hall C, Rimmer K, Davies J, Vivas L, Bacsa J (2010). Org Biomol Chem.

[121] Chauhan S S, Sharma M, Chauhan P M S (2010). Drug News Perspect.

[122] Fernandez I, Robert A (2011). Org Biomol Chem.

[123] Singh C, Verma V P, Naikade N K, Singh A S, Hassam M, Puri S K (2008). J Med Chem.

[124] Brown G D (2010). Molecules.

[125] Singh C, Sharma U, Saxena G, Puri S (2007). Bioorg Med Chem Lett.

[126] Singh C, Singh A S, Naikade N K, Verma V P, Hassam M, Gupta N, Pandey S (2010). Synthesis.

[127] Hencken C P, Kalinda A S, Gaetano D'Angelo J (2009). Annu Rep Med Chem.

[128] Šolaja B A, Terzić N, Pocsfalvi G, Gerena L, Tinant B, Opsenica D, Milhous W K (2002). J Med Chem.

[129] Kirchhofer C, Vargas M, Braissant O, Dong Y, Wang X, Vennerstrom J L, Keiser J (2011). Acta Trop.

[130] Vennerstrom J L, Fu H N, Ellis W Y, Ager A L, Wood J K, Andersen S L, Gerena L, Milhous W K (1992). J Med Chem.

[131] Hamann H-J, Hecht M, Bunge A, Gogol M, Liebscher J (2011). Tetrahedron Lett.

[132] Opsenica I, Terzić N, Opsenica D, Angelovski G, Lehnig L, Eilbracht P, Tinant B, Juranić Z, Smith K S, Yang Y S (2006). J Med Chem.

[133] Dong Y, McCullough K J, Wittlin S, Chollet J, Vennerstrom J L (2010). Bioorg Med Chem Lett.

[134] Cvijetic I N, Zizak Z P, Stanojkovic T P, Juranic Z D, Terzic N, Opsenica I M, Opsenica D M, Juranic I O, Drakulic B J (2010). Eur J Med Chem.

[135] Opsenica I, Opsenica D, Lanteri C A, Anova L, Milhous W K, Smith K S, Solaja B A (2008). J Med Chem.

[136] Bhattacharjee A K, Carvalho K A, Opsenica D, Solaja B A (2005). J Serb Chem Soc.

[137] Tonmunphean S, Wijitkosoom A, Tantirungrotechai Y (2004). Bioorg Med Chem.

[138] Ellis G L, Amewu R, Sabbani S, Stocks P A, Shone A, Stanford D, Gibbons P, Davies J, Vivas L, Charnaud S (2008). J Med Chem.

[139] Liu H-H, Wu Y-K, Shen X (2003). Chin J Chem.

[140] Dong Y (2002). Mini-Rev Med Chem.

[141] Opsenica D M, Terzić N, Smith P L, Yang Y, Anova L, Smith K S, Solaja B A (2008). Bioorg Med Chem.

[142] Terzić N, Opsenica D, Milić D, Tinan B, Smith K S, Milhous W K, Šolaja B A (2007). J Med Chem.

[143] Pis Diez R, Jubert A H (2000). J Mol Struct: THEOCHEM.

[144] Amewu R, Stachulski A V, Ward S A, Berry N G, Bray P G, Davies J, Labat G, Vivas L, O’Neill P M (2006). Org Biomol Chem.

[145] Dong Y, Matile H, Chollet J, Kaminsky R, Wood J K, Vennerstrom J L (1999). J Med Chem.

[146] Kumura N, Furukawa H, Kobayashi M, Onyango A N, Izumi M, Nakajima S, Kim H-S, Wataya Y, Baba N (2009). Biosci, Biotechnol, Biochem.

[147] Ingram K, Schiaffo C E, Sittiwong W, Benner E, Dussault P H, Keiser J (2012). J Antimicrob Chemother.

[148] Dong Y, Chollet J, Vargas M, Mansour N R, Bickle Q, Alnouti Y, Huang J, Keiser J, Vennerstrom J L (2010). Bioorg Med Chem Lett.

[149] Mohamed A E-H H, El-Sayed M A, Hegazy M E, Helaly S E, Esmail A M, Mohamed N S (2010). Rec Nat Prod.

[150] Yang Z-S, Wu W-M, Li Y, Wu Y-L (2005). Helv Chim Acta.

[151] Keiser J, Utzinger J (2010). Adv Parasitol.

[152] Dembitsky V, Shkrob I, Hanus L O (2008). Biomed Pap.

[153] Laurent S A-L, Boissier J, Coslédan F, Gornitzka H, Robert A, Meunier B (2008). Eur J Org Chem.

[154] Jung M, Lee K, Kim H, Park M (2004). Curr Med Chem.

[155] Xiao S-h, Mei J-y, Jiao P-y (2011). Parasitol Res.

[156] Boissier J, Coslédan F, Robert A, Meunier B (2009). Antimicrob Agents Chemother.

[157] Pradines V, Portela J, Boissier J, Cosledan F, Meunier B, Robert A (2011). Antimicrob Agents Chemother.

[158] Maurya R, Soni A, Anand D, Ravi M, Raju K S R, Taneja I, Naikade N K, Puri S K, Wahajuddin K, Anojiya S (2013). Med Chem Lett.

[159] Ingram K, Yaremenko I A, Krylov I, Hofer L, Terent'ev A O, Keiser J (2012). J Med Chem.

[160] Keiser J, Brun R, Fried B, Utzinger J (2006). Antimicrob Agents Chemother.

[161] Vennervald B J, Polman K (2009). Parasite Immunol.

[162] Yajima A, Gabrielli A F, Montresor A, Engels D (2011). Trans R Soc Trop Med Hyg.

[163] Keiser J, Utzinger J, Tanner M, Dong Y, Vennerstrom J L (2006). J Antimicrob Chemother.

[164] Cooper P J, Ayre G, Martin C, Rizzo J A, Ponte E V, Cruz A A (2008). Allergy (Oxford, U K).

[165] Vandemark L M, Jia T-W, Zhou X-N (2010). Adv Parasitol.

[166] Keiser J, Utzinger J (2007). Trends Parasitol.

[167] Brooker S, Akhwale W, Pullan R, Estambale B, Clarke S E, Snow R W, Hotez P J (2007). Am J Trop Med Hyg.

[168] Lv S, Zhang Y, Steinmann P, Zhou X-N, Utzinger J (2010). Adv Parasitol.

[169] Xiao S-H, Keiser J, Chollet J, Utzinger J, Dong Y, Endriss Y, Vennerstrom J L, Tanner M (2007). Antimicrob Agents Chemother.

[170] Cooper P J, Nutman T B (2002). Lung Biol Health Dis.

[171] Brooker S (2010). Int J Parasitol.

[172] Keiser J, Utzinger J (2007). Curr Opin Infect Dis.

[173] Feldmeier H, Chitsulo L (1999). Arzneim Forsch.

[174] Thun M J, Henley S J, Gansler T (2004). Novartis Found Symp.

[175] Niesen A, Barthel A, Kluge R, Köwitzsch A, Ströhl D, Schwarz S, Csuk R (2009). Arch Pharm Chem Life Sci.

[176] Sawant S S, Youssef D T A, Sylvester P W, Wali V, El Sayed K A (2007). Nat Prod Commun.

[177] Csuk R, Niesen-Barthel A, Barthel A, Kluge R, Ströhl D (2010). Eur J Med Chem.

[178] Rudi A, Afanii R, Gravalos L G, Aknin M, Gaydou E, Vacelet J, Kashman Y (2003). J Nat Prod.

[179] Szpilman A M, Korshin E E, Rozenberg H, Bachi M D (2005). J Org Chem.

[180] Berrue F, Thomas O P, Funel-Le Bon C, Reyes F, Amade P (2005). Tetrahedron.

[181] Rudi A, Kashman Y (1993). J Nat Prod.

[182] Yao G, Steliou K (2002). Org Lett.

[183] Jung M, Ham J, Song J (2002). Org Lett.

[184] Wijeratne E M K, Liu M X, Kantipudi N B, Brochini C B, Gunatilaka A A L, Canfield L M (2006). Bioorg Med Chem.

[185] Aldeco-Pérez E, Rudler H, Parlier A, Alvarez C, Apan M T, Herson P, Toscano A (2006). Tetrahedron Lett.

[186] Dembitsky V M, Gloriozova T A, Poroikov V V (2007). Mini-Rev Med Chem.

[187] Hang T T N, Hu'o'ng L M, Ha T T H, Ha L M (2009). Tap Chi Duoc Hoc.

[188] Ma Y-M, Feng C-L (2008). Youji Huaxue.

[189] Dai J, Liu Y, Zhou Y-D, Nagle D G (2007). J Nat Prod.

[190] Rubush D M, Morges M A, Rose B J, Thamm D H, Rovis T (2012). J Am Chem Soc.

[191] Dembitsky V M, Gloriozova T A, Poroikov V V (2005). Mini-Rev Med Chem.

[192] Matsugo S, Kayamori N, Konishi T (1986). Niigata Yakka Daigaku Kenkyu Hokoku.

[193] Ryden A-M, Kayser O (2007). Top Heterocycl Chem.

[194] Roshchina V V (2006). Uspekhi Sovrem Biol.

[195] Bradley D (2000). Drug Discovery Today.

[196] Jiménez M S, Garznm S P, Rodríguez A D (2003). J Nat Prod.

[197] Casteel D A (1999). Nat Prod Rep.

[198] Casteel D A (1992). Nat Prod Rep.

[199] Lee K R (1991). Saengyak Hakhoechi.

[200] Kasozi D M, Rahlfs S, Becker K (2011). Drug Discovery Infect Dis.

[201] Denisov E T, Solodova S L, Denisova T G (2010). Russ Chem Rev.

[202] Davies-Coleman M T (2005). Stud Nat Prod Chem.

[203] Mancini I, Guella G, Defant A (2008). Mini-Rev Med Chem.

[204] Lupulescu A (1996). Prostaglandins, Leukotrienes Essent Fatty Acids.

[205] Ridley R G, Hudson A T (1998). Curr Opin Infect Dis.

[206] Mohammed R, Peng J, Kelly M, Yousaf M, Winn E, Odde S, Bie Z, Xie A, Doerksen R J, Hamann M T (2010). Aust J Chem.

[207] Camuzat-Dedenis B, Provot O, Cointeaux L, Perroux V, Berrien J-F, Bories C, Loiseau P M, Mayrargue J (2001). Eur J Med Chem.

[208] Howarth J, Wilson D (2003). Bioorg Med Chem Lett.

[209] Van Assche T, Deschacht M, Inocencio da Luz R A, Maes L, Cos P (2011). Free Radical Biol Med.

[210] Lim C-W, Kim Y-K, Jang M S, Park J-I, Park H-Y (2006). J Fish Sci Technol.

[211] Oguri H, Hiruma T, Yamagishi Y, Oikawa H, Ishiyama A, Otoguro K, Yamada H, Omura S (2011). J Am Chem Soc.

[212] Feng Y-J, Davis R A, Sykes M, Avery V M, Camp D, Quinn R J (2010). J Nat Prod.

[213] Macreadie P, Avery T, Greatrex B, Taylor D, Macreadie I (2006). Bioorg Med Chem Lett.

[214] Manzo E, Ciavatta M L, Melck D, Schupp P, de Voogd N, Gavagnin M (2009). J Nat Prod.

[215] Dembitsky V M (2003). Tetrahedron.

[216] Dembitsky V M, Srebnik M (2002). Eurasian Chem–Technol J.

[217] Chen Y, Chilson K, Killday K B, Harmody D, McCarthy P J, Pomponi, S A, Schimoler R, Selitrennikoff C, Wright A E (2003). Cyclic peroxides as novel antifungal agents. U. S. Patent.

[218] Avery T D, Macreadie P I, Greatrex B W, Robinson T V, Taylor D K, Macreadie I G (2007). Bioorg Med Chem.

[219] Dembitsky V M, Srebnik M (2002). Prog Lipid Res.

[220] Fattorusso E, Parapini S, Campagnuolo C, Basilico N, Taglialatela-Scafati O, Taramelli D (2002). J Antimicrob Chemother.

[221] Macreadie I G, Avery T D, Robinson T V, Macreadie P, Barraclough M, Taylor D K, Tiekink E R T (2008). Tetrahedron.

[222] Kishi M, Maeno K, Natsuhara M, Kususe M (1999). Mold removal agent composition. Patent Jpn. Kokai Tokkyo Koho.

[223] Gunasekera S P, Gunasekera M, Gunawardana G P, McCarthy P, Burres N (1990). J Nat Prod.

[224] Phillipson D W, Rinehart K L (1983). J Am Chem Soc.

[225] Rugutt J K, Rugutt K J (2002). Nat Prod Lett.

[226] Modi I A, Ghosh P K, Bhardwaj D, Desai N M, Khamar B M (2008). P38 inhibitors. PCT Int. Appl..

[227] Kyle D E, Milhous W, Opsenica D M, Pocsfalvi G, Solaja B (2003). Mixed steroidal 1,2,4,5-tetraoxane compounds and methods of making and using thereof. PCT Int. Appl..

[228] Chen L-W, Cheng M-J, Peng C-F, Chen I-S (2010). Chem Biodiversity.

[229] Stratakis M, Orfanopoulos M (2000). Tetrahedron.

[230] Clennan E L, Pace A (2005). Tetrahedron.

[231] Frimer A A, Afri M, Baumel S D, Gilinsky-Sharon P, Rosenthal Z, Gottlieb H E (2000). J Org Chem.

[232] Cointeaux L, Berrien J-F, Mayrargue J (2002). Tetrahedron Lett.

[233] Adam W, Peters K, Peters E-M, Schambony S (2000). J Am Chem Soc.

[234] Barnier J-P, Morisson V, Blanco L (2001). Synth Commun.

[235] Kirihara M, Kakuda H, Ichinose M, Ochiai Y, Takizawa S, Mokuya A, Okubo K, Hatano A, Shiro M (2005). Tetrahedron.

[236] Kulinkovich O G, Astashko D A, Tyvorskii V I, Ilyina N A (2001). Synthesis.

[237] Wimalasena K, Wickman H B, Mahindaratne M P D (2001). Eur J Org Chem.

[238] Ouhamou N, Six Y (2003). Org Biomol Chem.

[239] Madelaine C, Buzas A K, Kowalska-Six J A, Six Y, Crousse B (2009). Tetrahedron Lett.

[240] Zhao Q, Wong H N C (2007). Tetrahedron.

[241] Ikeda H, Akiyama K, Takahashi Y, Nakamura T, Ishizaki S, Shiratori Y, Ohaku H, Goodman J L, Houmam A, Wayner D D M (2003). J Am Chem Soc.

[242] Ikeda H, Hoshi Y, Miyashi T (2001). Tetrahedron Lett.

[243] Abe M, Kawanami S, Masuyama A, Hayashi T (2006). J Org Chem.

[244] Gbur R K, Little R D (2012). J Org Chem.

[245] Tsubusaki T, Nishino H (2009). Tetrahedron.

[246] Isayama S, Mukaiyama T (1989). Chem Lett.

[247] Isayama S (1990). Bull Chem Soc Jpn.

[248] Tokuyasu T, Kunikawa S, Masuyama A, Nojima M (2002). Org Lett.

[249] Tokuyasu T, Kunikawa S, McCullough K J, Masuyama A, Nojima M (2005). J Org Chem.

[250] Dai P, Dussault P H (2005). Org Lett.

[251] Barnych B, Vatèle J-M (2012). Tetrahedron.

[252] Ghorai P, Dussault P H, Hu C (2008). Org Lett.

[253] Kumar D N, Sudhakar N, Rao B V, Kishore K H, Murty U S (2006). Tetrahedron Lett.

[254] Hamann H-J, Wlosnewski A, Greco T, Liebscher J (2006). Eur J Org Chem.

[255] Stewart S G, Ghisalberti E L, Skelton B W, Heath C H (2010). Org Biomol Chem.

[256] Dussault P H, Lee H-J, Liu X (2000). J Chem Soc, Perkin Trans 1.

[257] Dussault P H, Lee I Q, Lee H-J, Lee R J, Niu Q J, Schultz J A, Zope U R (2000). J Org Chem.

[258] Zhao Q, Vargas M, Dong Y, Zhou L, Wang X, Sriraghavan K, Keiser J, Vennerstrom J L (2010). J Med Chem.

[259] Ramirez A, Woerpel K A (2005). Org Lett.

[260] Dai P, Trullinger T K, Liu X, Dussault P H (2006). J Org Chem.

[261] Lu X, Liu Y, Sun B, Cindric B, Deng L (2008). J Am Chem Soc.

[262] Reisinger C M, Wang X, List B (2008). Angew Chem, Int Ed.

[263] Silva E M P, Pye R J, Brown G D, Harwood L M (2012). Eur J Org Chem.

[264] Guerra F M, Zubia E, Ortega M J, Moreno-Dorado F J, Massanet G M (2010). Tetrahedron.

[265] Singh C, Srivastav N C, Srivastava N, Puri S K (2005). Tetrahedron Lett.

[266] Kishali N, Sahin E, Kara Y (2006). Org Lett.

[267] Dussault P H, Xu C (2004). Tetrahedron Lett.

[268] Criegee R (1975). Angew Chem, Int Ed Engl.

[269] Geletneky C, Berger S (1998). Eur J Org Chem.

[270] Tokuyasu T, Ito T, Masuyama A, Nojima M (2000). Heterocycles.

[271] Nicolaou K C, Rodriguez R M, Mitchell H J, Suzuki H, Fylaktakidou K C, Baudoin O, van Delft F L (2000). Chem–Eur J.

[272] Hon Y-S, Lu L, Chang R-C, Lin S-W, Sun P-P, Lee C-F (2000). Tetrahedron.

[273] Odinokov V N, Akhmetova V R, Savchenko R G, Bazunova M V, Paramonov E A, Khalilov L M (2000). Russ Chem Bull.

[274] Odinokov V N, Akhmetova V R, Bazunova M V, Savchenko R G, Paramonov E A, Khalilov L M (2001). Russ J Org Chem.

[275] Dussault P H, Raible J M (2000). Org Lett.

[276] Jung I C (2001). Eur J Org Chem.

[277] Caronna T, Gabbiadini S, Mele A, Recupero F (2002). Helv Chim Acta.

[278] Kiryukhin D P, Barkalov I M, Ismoilov I L (2001). Russ J Appl Chem.

[279] Chen L, Wiemer D F (2002). J Org Chem.

[280] Rücker G, Manns D, Schenkel E P, Hartmann R, Heinzmann B M (2003). Arch Pharm Pharm Med Chem.

[281] Hitchcock P B, Papadopoulos K, Young D W (2003). Org Biomol Chem.

[282] Wattanasereekul S, Maier M E (2004). Adv Synth Catal.

[283] Schank K, Beck H, Pistorius S (2004). Helv Chim Acta.

[284] Laventine D M, Davies M, Evinson E L, Jenkins P R, Cullis P M, Fawcett J (2005). Tetrahedron Lett.

[285] Laventine D M, Davies M, Evinson E L, Jenkins P R, Cullis P M, Garcia M D (2009). Tetrahedron.

[286] Wang C, Liu J, Ji Y, Zhao J, Li L, Zhang H (2006). Org Lett.

[287] Schwartz C, Raible J, Mott K, Dussault P H (2006). Org Lett.

[288] Schwartz C, Raible J, Mott K, Dussault P H (2006). Tetrahedron.

[289] Schiaffo C E, Dussault P H (2008). J Org Chem.

[290] Percy J M, Roig R, Singh K (2009). Eur J Org Chem.

[291] Shin H S, Lee C, Lee J Y, Huh T S (2000). Eur J Org Chem.

[292] Park S H, Lee J Y, Huh T S (2001). Eur J Org Chem.

[293] Kawamura S-i, Yamakoshi H, Abe M, Masuyama A, Nojima M (2002). Tetrahedron.

[294] Griesbaum K, Frank A, McCullough K J (2006). Eur J Org Chem.

[295] Griesbaum K, Bikem Ö, Huh T S, Dong Y (1995). Liebigs Ann.

[296] Araújo N C P, Barton V, Jones M, Stocks P A, Ward S A, Davies J, Bray P G, Shone A E, Cristiano M L S, O’Neill P M (2009). Bioorg Med Chem Lett.

[297] Tang Y, Dong Y, Karle J M, DiTusa C A, Vennerstrom J L (2004). J Org Chem.

[298] Zhou L, Alker A, Ruf A, Wang X, Chiu F C K, Morizzi J, Charman S A, Charman W N, Scheurer C, Wittlin S (2008). Bioorg Med Chem Lett.

[299] Kamata M, Komatsu K-i, Akaba R (2001). Tetrahedron Lett.

[300] Li Y, Hao H-D, Zhang Q, Wu Y (2009). Org Lett.

[301] Kamata M, Ohta M, Komatsu K-i, Kim H-S, Wataya Y (2002). Tetrahedron Lett.

[302] Parrish J D, Ischay M A, Lu Z, Guo S, Peters N R, Yoon T P (2012). Org Lett.

[303] La Clair J J (2006). Angew Chem, Int Ed.

[304] Griesbeck A G, Cho M (2009). Tetrahedron Lett.

[305] Iwahama T, Sakaguchi S, Ishii Y (2000). Chem Commun.

[306] Kumabe R, Nishino H, Yasutake M, Nguyen V-H, Kurosawa K (2001). Tetrahedron Lett.

[307] Christoffers J, Werner T, Frey W, Baro A (2003). Eur J Org Chem.

[308] Rössle M, Werner T, Frey W, Christoffers J (2005). Eur J Org Chem.

[309] Kumabe R, Nishino H (2004). Tetrahedron Lett.

[310] Asahi K, Nishino H (2005). Tetrahedron.

[311] Asahi K, Nishino H (2008). Tetrahedron.

[312] Asahi K, Nishino H (2008). Eur J Org Chem.

[313] Beckwith A L J, Wagner R D (1979). J Am Chem Soc.

[314] Beckwith A L J, Wagner R D (1980). J Chem Soc, Chem Commun.

[315] Korshin E E, Hoos R, Szpilman A M, Konstantinovski L, Posner G H, Bachi M D (2002). Tetrahedron.

[316] O’Neill P M, Verissimo E, Ward S A, Davies J, Korshin E E, Araujo N, Pugh M D, Cristiano M L S, Stocks P A, Bachi M D (2006). Bioorg Med Chem Lett.

[317] Kim J, Li H B, Rosenthal A S, Sang D, Shapiro T A, Bachi M D, Posner G H (2006). Tetrahedron.

[318] Tokuyasu T, Kunikawa S, Abe M, Masuyama A, Nojima M, Kim H-S, Begum K, Wataya Y (2003). J Org Chem.

[319] Wu J-M, Kunikawa S, Tokuyasu T, Masuyama A, Nojima M, Kim H-S, Wataya Y (2005). Tetrahedron.

[320] Gemma S, Martí F, Gabellieri E, Campiani G, Novellino E, Butini S (2009). Tetrahedron Lett.

[321] Gemma S, Gabellieri E, Coccone S S, Martí F, Taglialatela-Scafati O, Novellino E, Campiani G, Butini S (2010). J Org Chem.

[322] Murakami N, Kawanishi M, Itagaki S, Horii T, Kobayashi M (2001). Tetrahedron Lett.

[323] Murakami N, Kawanishi M, Itagaki S, Horii T, Kobayashi M (2002). Bioorg Med Chem Lett.

[324] Murakami N, Kawanishi M, Itagaki S, Horii T, Kobayashi M (2004). Bioorg Med Chem Lett.

[325] Jin H-X, Liu H-H, Zhang Q, Wu Y (2005). Tetrahedron Lett.

[326] Jin H-X, Zhang Q, Kim H-S, Wataya Y, Liu H-H, Wu Y (2006). Tetrahedron.

[327] Jin H-X, Liu H-H, Zhang Q, Wu Y (2005). J Org Chem.

[328] Li Y, Zhang Q, Wittlin S, Jin H-X, Wu Y (2009). Tetrahedron.

[329] Xu C, Raible J M, Dussault P H (2005). Org Lett.

[330] Fontana A, d'Ippolito G, D'Souza L, Mollo E, Parameswaram P S, Cimino G (2001). J Nat Prod.

[331] Zhang Q, Li Y, Wu Y-K (2007). Chin J Chem.

[332] Xu C, Schwartz C, Raible J, Dussault P H (2009). Tetrahedron.

[333] Tokuyasu T, Masuyama A, Nojima M, McCullough K J (2000). J Org Chem.

[334] Tokuyasu T, Masuyama A, Nojima M, Kim H-S, Wataya Y (2000). Tetrahedron Lett.

[335] Kim H-S, Begum K, Ogura N, Wataya Y, Tokuyasu T, Masuyama A, Nojima M, McCullough K J (2002). J Med Chem.

[336] Harris J R, Waetzig S R, Woerpel K A (2009). Org Lett.

[337] O’Neill P M, Rawe S L, Storr R C, Ward S A, Posner G H (2005). Tetrahedron Lett.

[338] Tokuyasu T, Masuyama A, Nojima M, McCullough K J, Kim H-S, Wataya Y (2001). Tetrahedron.

[339] Rostami A, Wang Y, Arif A M, McDonald R, West F G (2007). Org Lett.

[340] Matsumoto M, Nasu S, Takeda M, Murakami H, Watanabe N (2000). Chem Commun.

[341] López D, Quiñoá E, Riguera R (2000). J Org Chem.

[342] Takabatake T, Miyazawa T, Hasegawa M, Foote C S (2001). Tetrahedron Lett.

[343] Özen R, Kormalı F, Balci M, Atasoy B (2001). Tetrahedron.

[344] Daştan A, Saracoglu N, Balci M (2001). Eur J Org Chem.

[345] Güney M, Daştan A, Balci M (2005). Helv Chim Acta.

[346] Daştan A, Balci M (2006). Tetrahedron.

[347] Güney M, Ceylan Z C, Daştan A, Balci M (2005). Can J Chem.

[348] Gu X, Zhang W, Salomon R G (2007). J Am Chem Soc.

[349] Blay G, Garcia B, Molina E, Pedro J R (2005). Org Lett.

[350] Yang Y-K, Lee S, Tae J (2004). Bull Korean Chem Soc.

[351] Yang Y-K, Choi J-H, Tae J (2005). J Org Chem.

[352] Avery T D, Caiazza D, Culbert J A, Taylor D K, Tiekink E R T (2005). J Org Chem.

[353] Van der Westhuyzen C W, Parkinson C J (2005). S Afr J Chem.

[354] Valente P, Avery T D, Taylor D K, Tiekink E R T (2009). J Org Chem.

[355] Kao T-C, Chuang G J, Liao C-C (2008). Angew Chem, Int Ed.

[356] Robinson T V, Pedersen D S, Taylor D K, Tiekink E R T (2009). J Org Chem.

[357] Schobert R, Siegfried S, Weingärtner J, Nieuwenhuyzen M (2001). J Chem Soc, Perkin Trans 1.

[358] Schobert R, Stehle R, Milius W (2003). J Org Chem.

[359] Gavrilan M, André-Barrès C, Baltas M, Tzedakis T, Gorrichon L (2001). Tetrahedron Lett.

[360] Najjar F, Baltas M, Gorrichon L, Moreno Y, Tzedakis T, Vial H, André-Barrès C (2003). Eur J Org Chem.

[361] Çetin F, Yenil N, Yüceer L (2005). Carbohydr Res.

[362] Margaros I, Montagnon T, Vassilikogiannakis G (2007). Org Lett.

[363] Zvarec O, Avery T D, Taylor D K (2010). J Org Chem.

[364] Zvarec O, Avery T D, Taylor D K, Tiekink E R T (2010). Tetrahedron.

[365] Robinson T V, Taylor D K, Tiekink E R T (2006). J Org Chem.

[366] Taylor D K, Avery T D, Greatrex B W, Tiekink E R T, Macreadie I G, Macreadie P I, Humphries A D, Kalkanidis M, Fox E N, Klonis N (2004). J Med Chem.

[367] del Pilar Crespo M, Avery T D, Hanssen E, Fox E, Robinson T V, Valente P, Taylor D K, Tilley L (2008). Antimicrob Agents Chemother.

[368] Emerzian M A, Davenport W, Song J, Li J, Erden I (2009). Adv Synth Catal.

[369] Özer G, Saraçoğlu N, Balci M (2003). J Org Chem.

[370] Singh C (1990). Tetrahedron Lett.

[371] Griesbeck A G, El-Idreesy T T, Fiege M, Brun R (2002). Org Lett.

[372] Singh C, Gupta N, Puri S K (2003). Bioorg Med Chem Lett.

[373] Singh C, Gupta N, Puri S K (2002). Bioorg Med Chem Lett.

[374] Singh C, Malik H, Puri S K (2004). Bioorg Med Chem Lett.

[375] Singh C, Malik H, Puri S K (2005). Bioorg Med Chem Lett.

[376] Singh C, Malik H (2005). Org Lett.

[377] Singh C, Malik H, Puri S K (2004). Bioorg Med Chem.

[378] Singh C, Kanchan R, Srivastava N C, Puri S K (2006). Bioorg Med Chem Lett.

[379] Singh C, Malik H, Puri S K (2006). J Med Chem.

[380] Singh C, Verma V P, Naikade N K, Singh A S, Hassam M, Puri S K (2010). Bioorg Med Chem Lett.

[381] Singh C, Gupta N, Puri S K (2004). Bioorg Med Chem.

[382] Singh C, Gupta N, Puri S K (2005). Tetrahedron Lett.

[383] Singh C, Srivastava N C, Puri S K (2004). Bioorg Med Chem.

[384] Griesbeck A G, El-Idreesy T T, Höinck L-O, Lex J, Brun R (2005). Bioorg Med Chem Lett.

[385] Bartoschek A, El-Idreesy T T, Griesbeck A G, Höinck L-O, Lex J, Miara C, Neudörfl J M (2005). Synthesis.

[386] Griesbeck A G, El-Idreesy T T, Lex J (2006). Tetrahedron.

[387] Sabbani S, La Pensée L, Bacsa J, Hedenström E, O’Neill P M (2009). Tetrahedron.

[388] Griesbeck A G, Höinck L-O, Neudörfl J M (2010). Beilstein J Org Chem.

[389] Griesbeck A G, Höinck L-O, Lex J, Neudörfl J, Blunk D, El-Idreesy T T (2008). Molecules.

[390] Singh C, Kanchan R, Sharma U, Puri S K (2007). J Med Chem.

[391] Griesbeck A G, Blunk D, El-Idreesy T T, Raabe A (2007). Angew Chem, Int Ed.

[392] Griesbeck A G, Raabe A (2009). Synlett.

[393] O’Neill P M, Mukhtar A, Ward S A, Bickley J F, Davies J, Bachi M D, Stocks P A (2004). Org Lett.

[394] Jefford C W, Velarde J A, Bernardinelli G, Bray D H, Warhurst D C, Milhous W K (1993). Helv Chim Acta.

[395] Posner G H, Maxwell J P, O'Dowd H, Krasavin M, Xie S, Shapiro T A (2000). Bioorg Med Chem.

[396] Posner G H, Jeon H B, Parker M H, Krasavin M, Paik I-H, Shapiro T A (2001). J Med Chem.

[397] Posner G H, Jeon H B, Ploypradith P, Paik I-H, Borstnik K, Xie S, Shapiro T A (2002). J Med Chem.

[398] Dechy-Cabaret O, Benoit-Vical F, Loup C, Robert A, Gornitzka H, Bonhoure A, Vial H, Magnaval J-F, Séguéla J-P, Meunier B (2004). Chem–Eur J.

[399] Singh R, Ishar M P S (2003). Tetrahedron Lett.

[400] Cole K P, Hsung R P (2005). Chem Commun.

[401] Tang Y, Cole K P, Buchanan G S, Li G, Hsung R P (2009). Org Lett.

[402] Borsarelli C D, Mischne M, La Venia A, Morán Vieyra F E (2007). Photochem Photobiol.

[403] O’Neil P M, Pugh M, Davies J, Ward S A, Park B K (2001). Tetrahedron Lett.

[404] O’Neill P M, Hindley S, Pugh M D, Davies J, Bray P G, Park B K, Kapu D S, Ward S A, Stocks P A (2003). Tetrahedron Lett.

[405] Tang Y, Dong Y, Wang X, Sriraghavan K, Wood J K, Vennerstrom J L (2005). J Org Chem.

[406] Erhardt S, Macgregor S A, McCullough K J, Savill K, Taylor B J (2007). Org Lett.

[407] Sabbani S, Stocks P A, Ellis G L, Davies J, Hedenstrom E, Ward S A, O’Neill P M (2008). Bioorg Med Chem Lett.

[408] Ramirez A P, Thomas A M, Woerpel K A (2009). Org Lett.

[409] Zhang Q, Jin H-X, Wu Y (2006). Tetrahedron.

[410] Zhang Q, Wu Y (2007). Tetrahedron.

[411] Riveira M J, La-Venia A, Mischne M P (2010). Tetrahedron Lett.

[412] Bellot F, Coslédan F, Vendier L, Brocard J, Meunier B, Robert A (2010). J Med Chem.

[413] Vennerstrom J L, Dong Y, Andersen S L, Ager A L, Fu H, Miller R E, Wesche D L, Kyle D E, Gerena L, Walters S M (2000). J Med Chem.

[414] McCullough K J, Wood J K, Bhattacharjee A K, Dong Y, Kyle D E, Milhous W K, Vennerstrom J L (2000). J Med Chem.

[415] Berkessel A, Andreae M R M, Schmickler H, Lex J (2002). Angew Chem, Int Ed.

[416] Ayala D A, Romero J M, Jorge N L, Gómez-Vara M E, Jubert A H, Castro E A (2006). Spectrochim Acta, Part A: Mol Biomol Spectrosc.

[417] Opsenica D, Pocsfalvi G, Juranić Z, Tinant B, Declercq J-P, Kyle D E, Milhous W K, Šolaja B A (2000). J Med Chem.

[418] Opsenica D, Angelovski G, Pocsfalvi G, Juranić Z, Žižak Ž, Kyle D, Milhous W K, Šolaja B A (2003). Bioorg Med Chem.

[419] Iskra J, Bonnet-Delpon D, Bégué J P (2003). Tetrahedron Lett.

[420] Žmitek K, Stavber S, Zupan M, Bonnet-Delpon D, Charneau S, Grellier P, Iskra J (2006). Bioorg Med Chem.

[421] Žmitek K, Stavber S, Zupan M, Bonnet-Delpon D, Iskra J (2006). Tetrahedron.

[422] Atheaya H, Khan S I, Mamgain R, Rawat D S (2008). Bioorg Med Chem Lett.

[423] Ghorai P, Dussault P H (2009). Org Lett.

[424] Terent'ev A O, Kutkin A V, Starikova Z A, Antipin M Y, Ogibin Y N, Nikishin G I (2004). Synthesis.

[425] Terent'ev A O, Kutkin A V, Platonov M M, Vorontsov I I, Antipin M Y, Ogibin Y N, Nikishin G I (2004). Russ Chem Bull.

[426] Kumar N, Khan S I, Sharma M, Atheaya H, Rawat D S (2009). Bioorg Med Chem Lett.

[427] Kumar N, Khan S I, Beena R, Rajalakshmi G, Kumaradhas P, Rawat D S (2009). Bioorg Med Chem.

[428] Terent'ev A O, Borisov D A, Chernyshev V V, Nikishin G I (2009). J Org Chem.

[429] Terent'ev A O, Yaremenko I A, Vil’ V A, Moiseev I K, Kon’kov S A, Dembitsky V M, Levitsky D O, Nikishin G I (2013). Org Biomol Chem.

[430] Kukovinets O S, Zvereva T I, Kabalnova N N, Kasradze V G, Salimova E V, Khalitova L R, Abdullin M I, Spirikhin L V (2009). Mendeleev Commun.

[431] Dong Y, Vennerstrom J L (2001). J Heterocycl Chem.

[432] Dong Y, Vennerstrom J L (1998). J Org Chem.

[433] Song C E, Lim J S, Kim S C, Lee K-J, Chi D Y (2000). Chem Commun.

[434] Opsenica D, Pocsfalvi G, Milhous W K, Šolaja B A (2002). J Serb Chem Soc.

[435] Terent’ev A O, Kutkin A V, Platonov M M, Ogibin Y N, Nikishin G I (2003). Tetrahedron Lett.

[436] Dubnikova F, Kosloff R, Almog J, Zeiri Y, Boese R, Itzhaky H, Alt A, Keinan E (2005). J Am Chem Soc.

[437] Opsenica I, Opsenica D, Smith K S, Milhous W K, Šolaja B A (2008). J Med Chem.

